# Integrated Multiomics Unravels Hedgehog (HH) Signaling Characteristics in Pancreatic Cancer (PC) and DCBLD2 Regulates HH Signaling to Drive PC Progression

**DOI:** 10.1155/humu/4806397

**Published:** 2025-12-11

**Authors:** Biao Zhang, Bingqian Huang, Xinya Zhao, Bolin Zhang, Jinming Liu, Chongchan Bao, Zhizhou Wang, Sujit Nair

**Affiliations:** ^1^ Department of General Surgery, The First Affiliated Hospital of Dalian Medical University, Dalian, China, dlmedu.edu.cn; ^2^ Key Laboratory of Clinical Cancer Pharmacology and Toxicology Research of Zhejiang Province, Department of Clinical Pharmacy, Affiliated Hangzhou First People’s Hospital, Westlake University, Hangzhou, China, westlake.edu.cn; ^3^ Department of Pharmacy, First Affiliated Hospital of Dalian Medical University, Dalian, China, dlmedu.edu.cn; ^4^ Department of Visceral, Martin-Luther-University Halle-Wittenberg, University Medical Center Halle, Halle, Germany, uni-halle.de; ^5^ Department of Breast and Thyroid Surgery, Affiliated Hospital of Youjiang Medical University for Nationalities, Baise, China, gxyyfy.cn; ^6^ Key Laboratory of Molecular Pathology in Tumors of Guangxi, Affiliated Hospital of Youjiang Medical University for Nationalities, Baise, China, gxyyfy.cn

**Keywords:** DCBLD2, diagnosis, hedgehog signaling, pancreatic cancer, prognosis, single-cell analysis, treatment

## Abstract

Hedgehog (HH) signaling plays a crucial role in cancer development. However, HH signaling–related molecular characteristics have not been comprehensively evaluated in pancreatic cancer (PC). This study dissected the characteristics of HH signaling in PC using integrated bulk and single‐cell profiling. GSEA indicated that HH signaling is significantly enriched in PC tissue. Consensus clustering was utilized to classify PC samples into two HH signaling–related subtypes: HRGcluster A and HRGcluster B. In contrast with HRGcluster A, HRGcluster B has an earlier clinical stage, better outcome, less active level of HH signaling, higher infiltration level of CD8+ T cells and B cells, and a greater likelihood of benefiting from immunotherapy and gemcitabine chemotherapy. Moreover, an HH signaling–related prognostic model (including ANLN, SERPINB3, LY6D, and DCBLD2) with excellent prediction performance was established and validated. Further analysis indicated that ANLN, SERPINB3, LY6D, and DCBLD2 were significantly upregulated in PC and associated with poor prognosis. Single‐cell analysis revealed that HH signaling is relatively more active in PC cells, and PC cells with DCBLD2 high expression had significantly higher HH signaling scores. In vitro assays further indicated that DCBLD2 knockdown downregulates HH signaling and inhibits the proliferation, migration, and invasion of PC cells. In conclusion, this study reveals that HH signaling characteristics in PC and DCBLD2 regulate HH signaling to drive PC progression, providing new perspectives and theories for the diagnosis and treatment of PC.

## 1. Introduction

Pancreatic cancer (PC) represents a significant challenge in oncology due to its aggressive nature and poor prognosis, with a 5‐year survival rate of <10% [1]. Only 20% of PC patients are diagnosed early enough to undergo curative surgery [2]. Adjuvant chemotherapy and radiotherapy are commonly used postoperatively to reduce the risk of PC recurrence. For patients with locally advanced or metastatic PC, systemic chemotherapy remains the mainstay, aiming to palliate symptoms and extend survival. However, PC shows low sensitivity to chemoradiotherapy. The complex tumor microenvironment (TME) can contribute to drug resistance in PC. For example, the crosstalk between macrophages and PC cells promotes tumor stem cell characteristics, leading to PC recurrence and metastasis [3]. In recent years, targeted therapy and immunotherapy have shown promise in clinical trials, providing new avenues for treatment. However, challenges such as tumor heterogeneity and resistance mechanisms continue to complicate treatment outcomes. Therefore, exploring the pathogenesis of PC and identifying its distinct molecular subtypes can help predict prognosis and develop novel therapeutic approaches.

In recent years, the hedgehog (HH) signaling pathway has emerged as a critical player in PC biology. HH signaling contains HH ligands (SHH, DHH, and IHH), patch receptors (PTCH1 and PTCH2), smoothened (SMO), motor protein KIF7, protein kinase A (PKA), three Gli transcription factors Gli1/2/3, and suppressor of fused (Sufu, a fusion inhibitory factor and negative regulator of HH signal transduction) [4]. HH signaling exhibits aberrant activation in various cancer types, contributing to tumor cell multiplication and invasion and the expansion of cancer stem cells (CSCs) [5]. Furthermore, HH signaling is closely associated with cellular transformation, tumorigenesis, and resistance to various cancer therapies [6]. The oncogenic mechanisms of HH signaling could be categorized into Type I mutation‐driven mechanisms (independent of the HH ligand), Type II (HH ligand‐dependent mechanisms), and Type IIIa/b (paracrine or reverse paracrine mode of ligand‐dependent HH signal transduction) [6]. Inactivation mutations in PTCH1 were found in 85% of sporadic basal cell carcinomas (BCCs), in addition to gain‐of‐function SMO mutations and/or loss‐of‐function Sufu mutations, which were also observed in BCC, medulloblastoma, and rhabdomyosarcoma [7]. In many tumors, including gastric, esophageal, colorectal, and ovarian cancers, overexpression of HH ligands has been detected, and ectopic expression of PTCH1 and Gli has also been found in these tumors [8, 9]. Bausch et al. [10] showed that inhibition of HH signaling can affect the growth and angiogenesis of PC. Lei et al. [11] found that HH signaling can modulate epithelial–mesenchymal transformation and invasion in hypoxia‐induced PC in a ligand‐independent manner. Within the TME, there exists an intricate metabolic milieu where tumor and stroma cells coexist. Immunocytes in TME are highly responsive to their surroundings, capable of dynamically modifying their metabolic profiles to exert regulatory effects when necessary [12]. Steele et al. [13] showed that inhibition of HH signaling could lead to a decrease in cytotoxic T cells and an increase in regulatory T cells (Tregs) in the PC microenvironment. Thus, the inhibition of HH signaling not only directly targets tumor cells but also reshapes the immune landscape within TME, inducing an immune‐activated state. Olive et al. [14] showed that inhibiting HH signaling promotes the delivery of chemotherapy drugs in a mouse model of PC. HH signaling also influences CSCs, which are often resistant to antitumor therapy and contribute to tumor relapse. Recent studies have revealed that CSCs can respond to HH ligands secreted by neighboring stromal cells, tumor cells, or even CSCs themselves. By the regulation of pluripotency genes like Nanog, Sox2, and Bmi1, HH signaling can maintain the stemness characteristics of CSCs [15]. Huang et al. [16] suggested that HH signaling was involved in the self‐renewal and chemotherapy resistance of PC stem cells. Therefore, dysregulation of HH signaling has been implicated in tumor initiation, progression, and metastasis, making it an attractive target for therapeutic intervention.

In this study, we presented a comprehensive analysis integrating bulk and single‐cell RNA sequencing (scRNA‐seq) to elucidate the complex characteristics of HH signaling in PC. Furthermore, through comprehensive bioinformatics analysis and in vitro experiments, we revealed that DCBLD2 promotes PC progression by regulating HH signaling. These results provide new insights and a theoretical basis for the diagnosis and treatment of PC.

## 2. Materials and Methods

### 2.1. Data Acquisition and Preprocessing

Transcriptome and clinical data were collected via The Cancer Genome Atlas (TCGA, https://portal.gdc.cancer.gov) platform (TCGA‐PC dataset, encompassing 185 PC patients) and Gene Expression Omnibus (GEO, https://www.ncbi.nlm.nih.gov/geo) platform (GSE62452 dataset, encompassing 69 PC patients; GSE28735 dataset, encompassing 45 PC patients; and GSE57495 dataset, encompassing 63 PC patients). Furthermore, the data on genetic information in 33 tumors was collected via the TCGA database. To mitigate batch effects between separate PC cohorts, we utilized the “sva” package (Version 3.50.0) [17, 18]. Samples whose survival was less than 30 days were excluded from the prognostic analysis. Hedgehog signaling–related genes (HRGs), consisting of a total of 56 genes (Supporting Information 2: Table S1), were obtained via the Molecular Signatures Database (MSigDB, http://www.gsea-msigdb.org) [19]. Univariate Cox regression was applied to select prognosis‐linked HRGs [20].

### 2.2. Consensus Clustering

Clustering analysis based on the expression of prognosis‐linked HRGs was implemented using the “ConsensusClusterPlus” package (Version 1.70.0) [21, 22]. The optimal number of clusters was determined by assessing the cumulative distribution function (CDF) curve and changes in the area of the CDF. The principal component analysis (PCA) as well as *t*‐distributed stochastic neighbor embedding (t‐SNE), known for their effective dimensionality reduction capabilities, were employed to validate our classification results [23, 24]. The prognosis between different molecular subtypes was contrasted utilizing Kaplan–Meier (KM) curves. The activity of HH signaling between different molecular subtypes was assessed by the ssGSEA algorithm.

### 2.3. Gene Set Variation Analysis (GSVA) and Gene Set Enrichment Analysis (GSEA)

To understand the underlying mechanisms of differences between various subtypes, GSVA and GSEA were implemented [25, 26]. The “c2.cp.kegg.v7.5.1.symbols.gmt” was utilized as the reference gene set.

### 2.4. Immunoassay and Drug Sensitivity Analysis

To seek the immunological characteristics among different molecular subtypes, ssGSEA was implemented to calculate the immunocyte‐infiltrated score for every PC sample. Additionally, the TME characteristics between different molecular subtypes were further evaluated using the CIBERSORT algorithm [27]. Samples with a *p* value < 0.05 indicate an accurate assessment of the proportions of the 22 immune cell subtypes generated by CIBERSORT, which could be utilized for further analysis. Tumor Immune Dysfunction and Exclusion (TIDE) platform (http://tide.dfci.harvard.edu/) was utilized to calculate T cell dysfunction and exclusion, MSI, and TIDE scores in PC patients for predicting immunotherapy response [28]. A lower TIDE score indicates that patients are more likely to benefit from immunotherapy. The “oncoPredict” package (Version 1.2) is commonly employed for predicting drug responses and identifying biomarkers in cancer patients or in vivo, based on cell line screening data [29]. It was utilized in this study to assess variations in drug sensitivity among patients of different molecular subtypes.

### 2.5. Differential Expression Analysis and Enrichment Analysis

To further investigate the dissimilarities among different molecular subtypes, we utilized the “limma” package (Version 3.62.2) to screen differentially expressed genes (DEGs), which were referred to as hedgehog signaling–related differentially expressed genes (HRDEGs). The filtering conditions were Log_2_(fold change (FC)) >1 and the adjusted *p* value < 0.05 [30]. Subsequently, we employed Gene Ontology (GO) and Kyoto Encyclopedia of Genes and Genomes (KEGG) enrichment analyses to seek the biological functions that were associated with HRDEGs. To evaluate the relationship between these HRDEGs and prognosis, clustering analysis was implemented utilizing the “ConsensusClusterPlus” package based on the expression of HRDEGs.

### 2.6. Construction and Validation of Prognostic Model

For assessing the individual prognosis of each sample, we employed LASSO regression as well as Cox regression to develop a prognostic model for PC utilizing HRDEGs [31]. A 5:5 ratio was utilized to arbitrarily split TCGA‐PC samples into the training and internal validation sets, while GSE28735, GSE62452, and GSE57495 cohorts were utilized as the external validation set. Univariable COX regression was used to initially screen prognostic genes, then LASSO regression was used to eliminate overfitting between genes, and multivariable COX regression was used to further screen prognostic genes and construct a model. The risk score was calculated using the predict function. Simultaneously, every patient could be categorized into high‐ or low‐score groups by contrasting its score with the median score in the training set. The prognosis between different score groups was contrasted using KM curves [32]. The time‐dependent receiver operating characteristic (ROC) curves and area under the curve (AUC) were utilized to evaluate the predictive performance of the model [33, 34].

### 2.7. Expression of Model Genes

GEPIA platform (http://gepia.cancer-pku.cn/) is an online platform that allows for gene differential, correlation, and survival analyses using the TCGA and GTEx databases [35]. UALCAN platform (https://ualcan.path.uab.edu/index.html) can perform differential and survival analyses of target genes at the protein level using tumor proteomic data from the CPTAC database [36]. The GEPIA and UALCAN platforms were utilized to analyze the expression of model genes at the RNA and protein levels, respectively. HPA database, known as the Human Protein Atlas (https://www.proteinatlas.org/), is a publicly available resource made to generate specific patterns of protein expression in cancerous and healthy tissues [37]. We obtained immunohistochemistry and immunofluorescence images for model genes via the HPA database.

### 2.8. Single‐Cell Analysis

scRNA‐seq data from three primary PCs were downloaded via the GSE197177 dataset. The “Seurat” package (Version 4.4.0) was utilized to convert single‐cell data into Seurat objects [38]. The following parameters were used for quality control of scRNA‐seq data: min.cells = 3, min.features = 200, nFeature_RNA > 500, nFeature_RNA < 7500, and percent.mt < 20. The “NormalizeData” function was used to normalize the scRNA‐seq. PCA was performed using the first 2000 highly variable genes. The “Harmony” function was used to remove batch effects between samples. The “FindNeighbors” and “FindClusters” functions were used to evaluate similarities between cells and cluster cells, respectively. Dimension reduction and visualization were further performed using UMAP. Firstly, the Human Primary Cell Atlas Data was used to annotate the cell subsets [39]. Then, the cell subpopulations were annotated using the expression of classical marker genes reported in the literature [40, 41]. The two annotation methods were combined to make the final annotation of the cells. Three algorithms, UCell, singscore, and AddModuleScore, were used to calculate the activity of HH signaling in different cell subsets. Copy number variation (CNV) for different cell subpopulations was calculated using inferCNV [42].

### 2.9. Cell Culture and siRNA Transfection

Two PC cell lines, PANC‐1 (RRID: CVCL_0480) and BxPC‐3 (RRID: CVCL_0186), were used in this study. PANC‐1 and BxPC‐3 were cultured with Dulbecco′s modified Eagle medium (DMEM) and RPMI‐1640 medium containing 10% fetal bovine serum (FBS) (Gibco, United States), respectively. The culture environment was 37°C with a 5% CO_2_ concentration. The siRNA transfection was implemented via Transfect‐Mate according to the instructions (GenePharma, Suzhou, China). The siRNA sequences for DCBLD2 were as follows: siRNA#1: 5 ^′^‐CAUACUCUGUUAUAGAUAATT‐3 ^′^; siRNA#2: 5 ^′^‐GGAUGUCAGUUUAUUCCUATT‐3 ^′^.

### 2.10. Real‐Time Quantitative PCR (qRT‐PCR)

Total RNAs of PANC‐1 and BxPC‐3 were extracted by TRIzol reagent. The reverse transcription reagent was implemented to obtain cDNA. Subsequently, qRT‐PCR was performed. The reference standard was GAPDH. The *ΔΔ*Ct technique was utilized to quantify the relative RNA expression. The primer sequences of SERPINB3, LY6D, DCBLD2, and ANLN were derived from GenePharma (Suzhou, China) (Supporting Information 2: Table S2).

### 2.11. CCK‐8 Assay

PANC‐1 and BxPC‐3 were cultured using 96‐well plates. On Days 0, 1, 2, and 3, PANC‐1 and BxPC‐3 cells were incubated with 100 *μ*L DMEM and 1640 medium containing 10% CCK‐8 enhancement reagent at 37°C sheltered from light for 2 h; then, OD values were measured at 450 nm to assess cell viability. The cell viability fold was calculated by dividing the OD value measured each day by the OD value on Day 0.

### 2.12. Scratch Assay

Scratch assay was implemented to examine the migration capacity of PANC‐1 and BxPC‐3 cells. PANC‐1 and BxPC‐3 were cultured in 6‐well plates and scratched with a 200‐*μ*L pipette tip when the cell density approached 100%. The images were then taken at 0 and 1 days, and the scratch area was measured using the ImageJ software. The healing rate of the scratch was calculated by dividing the difference between the area on Day 0 and the area on Day 1 by the area on Day 0.

### 2.13. Transwell Invasion Assay

To detect the invasiveness of PANC‐1 and BxPC‐3 cells, a Transwell invasion assay was performed. Then, 1 × 10^5^ cells were cultured using serum‐free DMEM or IMDM in a Transwell chamber (Corning, NY, United States) with prepositioned Matrigel. DMEM or 1640 medium, including 10% FBS, was placed in the lower chamber. After 48 h, the number of cells that had passed through the Transwell chamber was counted to assess the cells′ ability to invade.

### 2.14. Data Analysis

Data analysis and visualization were conducted using R language software (Version 4.1.2) and GraphPad Prism 9. Differences between the two groups were construed using either the *t*‐test or the Wilcoxon rank sum test. The KM method was utilized for prognostic assessment. *p* values < 0.05 were statistically significant.

## 3. Results

### 3.1. Identification, Prognostic Values, and Pan‐Cancer Analysis of HRGs

HH signaling pathway is presented in Figure 1a. GSEA revealed significant enrichment of the HH pathway in pancreatic tumor tissues, suggesting a close association between the HH pathway and PC (Figure 1b). Univariate Cox regression manifested that 24 genes within the HH pathway were associated with PC prognosis, with 10 genes (WNT9B, WNT2B, WNT10B, WNT4, WNT1, PTCH1, PRKACA, CSNK1D, BMP6, and BMP5) associated with a better prognosis and 14 genes (WNT7B, WNT7A, WNT5A, WNT2, WNT10A, SUFU, RAB23, GSK3B, GLI2, GAS1, CSNK1E, CSNK1A1, BMP8A, and BMP4) associated with a poorer prognosis (Figure 1c).

Figure 1The significance of the hedgehog (HH) signaling pathway in pancreatic cancer (PC) occurrence and prognosis. (a) HH signaling pathway mechanism diagram. (b) GSEA revealed that the HH signaling pathway was significantly enriched in PC tissue. (c) Univariate Cox regression identified that 24 genes within the HH pathway were associated with PC prognosis, with 10 genes associated with a better prognosis and 14 genes associated with a poorer prognosis.

**Figure:**
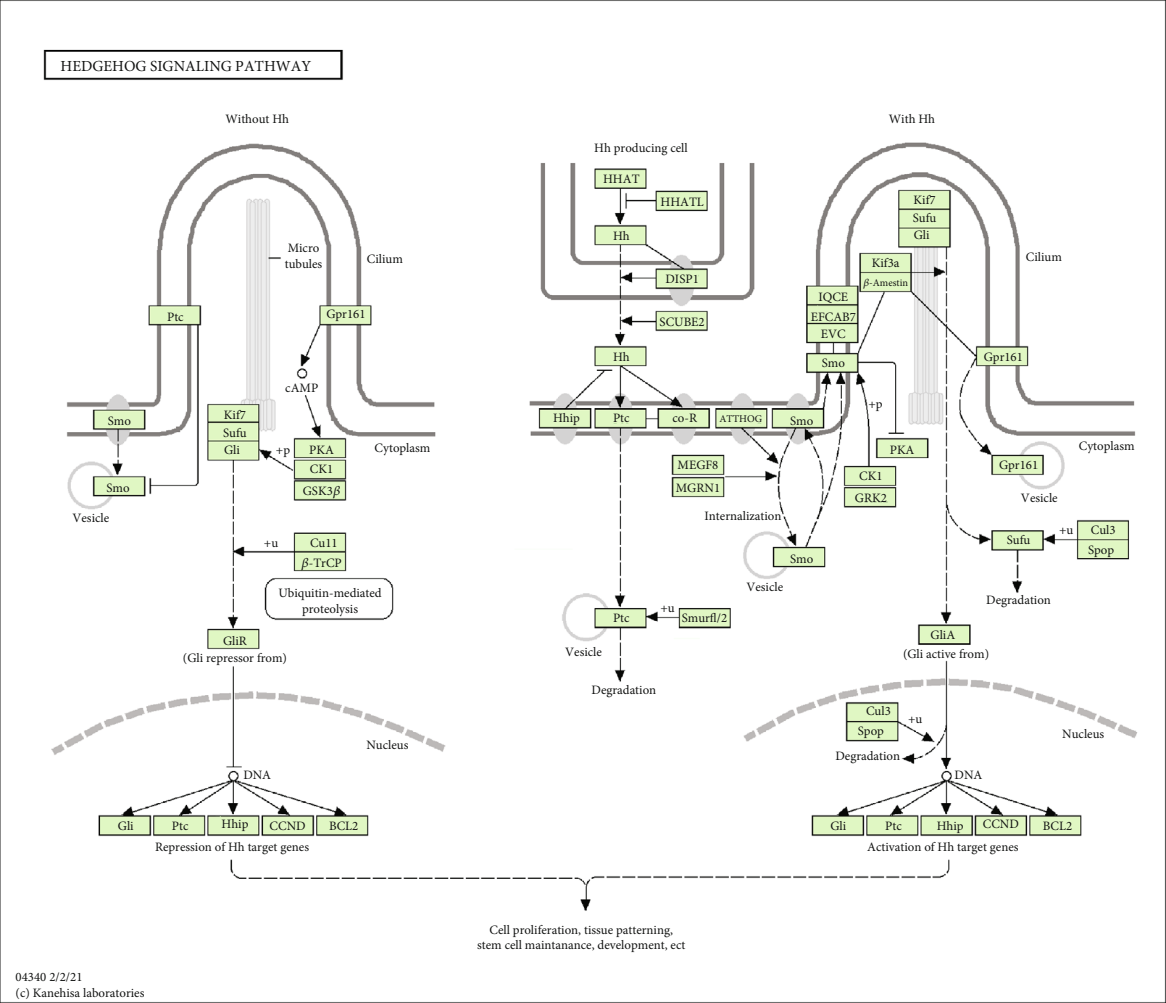
(a)

**Figure:**
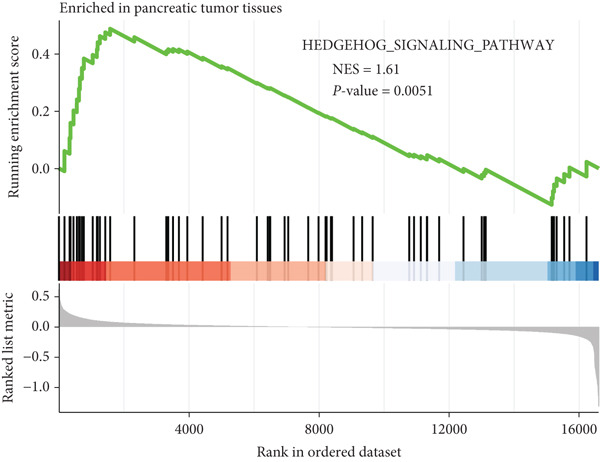
(b)

**Figure:**
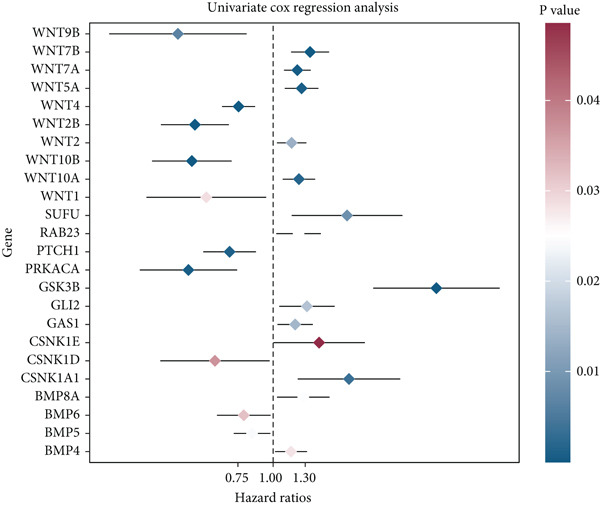
(c)

This study further investigated the genetic variations of these prognostically relevant HRGs in pan‐cancer, containing CNV, methylation, and mutations. We found that CNV of these HRGs was common in 33 types of cancer, with frequencies ranging from 5% to 80%. The CNV types of CSNK1A1, CSNK1D, GSK3B, PRKACA, WNT2, and WNT10B were mainly amplifications, while BMP8A, SUFU, WNT4, and WNT2B were mainly deletions (Figure 2a). Further analysis showed a positive correlation between the CNV of CSNK1A1, CSNK1D, CSNK1E, GSK3B, SUFU, and PRKACA and their expression, while the CNV of the remaining genes showed a negative correlation with expression (Figure 2b). Besides CNV, single‐nucleotide variation (SNV) was also identified as genetic variations influencing gene expression. We found that GLI2 (16%), PTCH1 (14%), and BMP5 (10%) had the highest SNV frequencies (Figure 2c). Among the 33 cancers, colon adenocarcinoma (COAD), skin cutaneous melanoma (SKCM), and uterine corpus endometrial carcinoma (UCEC) showed relatively higher SNV frequencies (Figure 2d). Then, the variations in methylation patterns for tumor tissues as well as normal tissues were investigated. Our findings revealed that, with the exception of GLI2 and CSNK1E, the majority of these prognostic‐related HRGs exhibited elevated levels of methylation within tumor tissues (Figure 2e). This observation indicated an underlying connection between aberrant methylation patterns and the dysregulation of HH signaling within tumorigenesis. Methylation of GLI2, GSK3B, WNT10B, WNT9B, and WNT7B showed a positive correlation with expression, while methylation of CSNK1E, PTCH1, BMP4, WNT5A, and WNT7A showed a negative correlation with expression (Figure 2f).

Figure 2Pan‐cancer analysis of hedgehog signaling–related prognostic genes (HRPGs). (a) Copy number variation (CNV) frequency of 24 HRPGs in 33 kinds of tumors. (b) The correlation between CNV and expression of 24 HRPGs in 33 kinds of tumors (red is positive and blue is negative). (c) Waterfall map of single‐nucleotide mutations (SNV) of 24 HRPGs in pan‐cancer. (d) The SNV frequency of 24 HRPGs in pan‐cancer. (e) Differences in methylation of 24 HRPGs between tumor tissue and normal tissue. Red represents increased methylation levels in the tumor, and blue represents decreased methylation levels in the tumor. (f) The correlation between methylation and expression of 24 HRPGs in pan‐cancer (red is positive and blue is negative).

**Figure:**
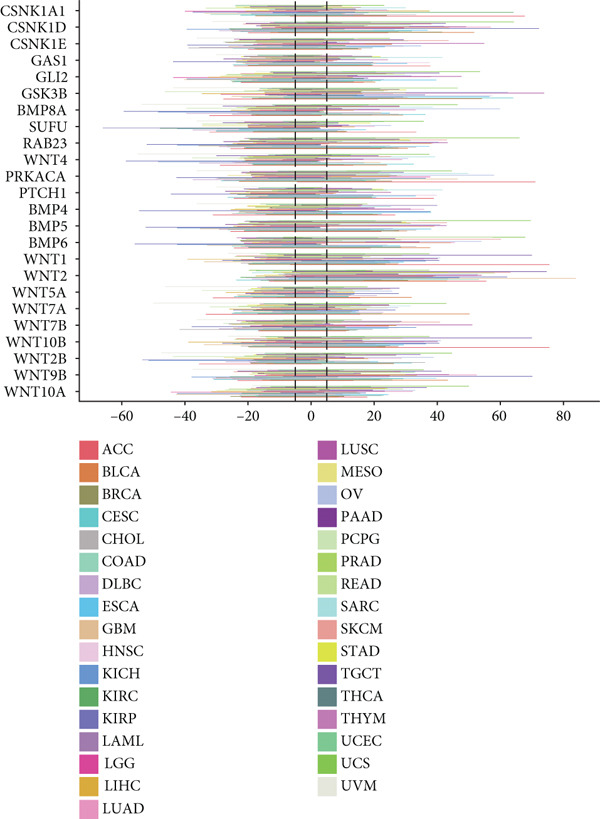
(a)

**Figure:**
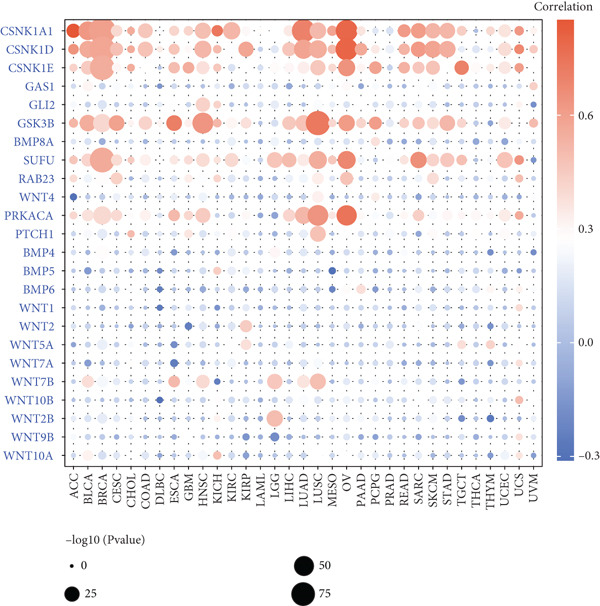
(b)

**Figure:**
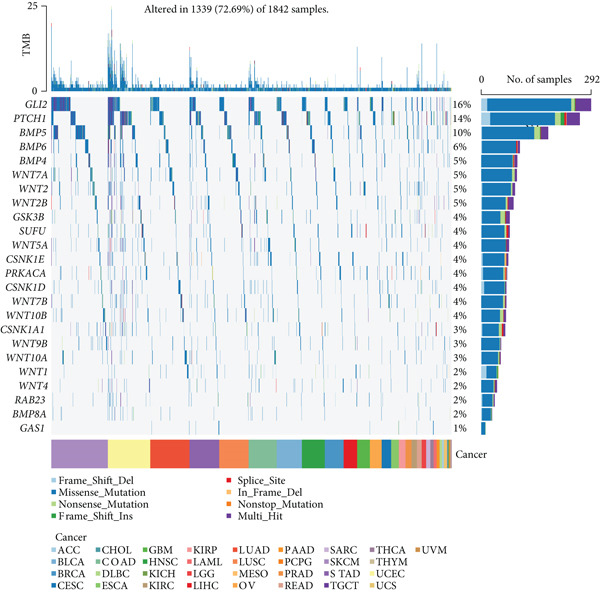
(c)

**Figure:**
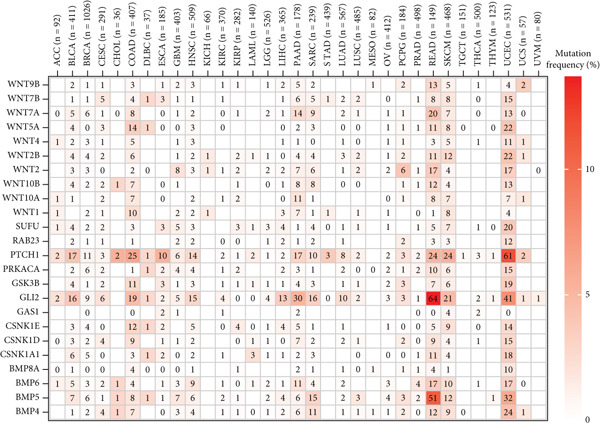
(d)

**Figure:**
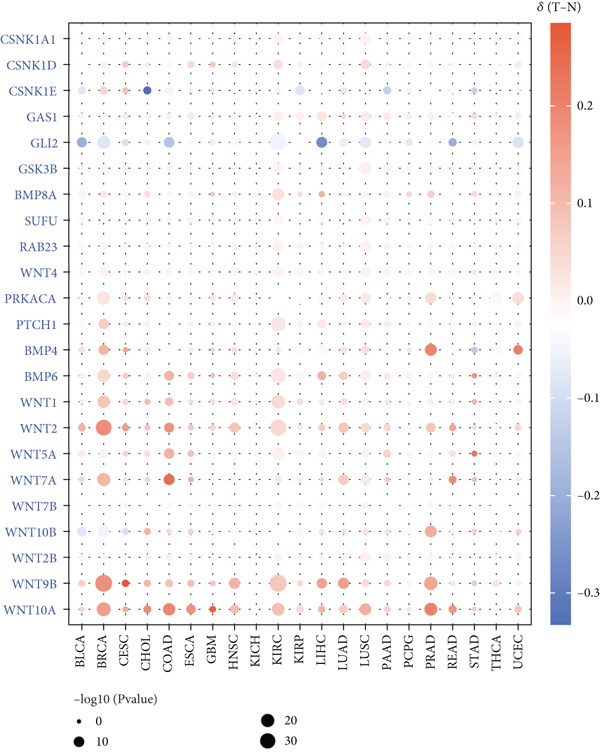
(e)

**Figure:**
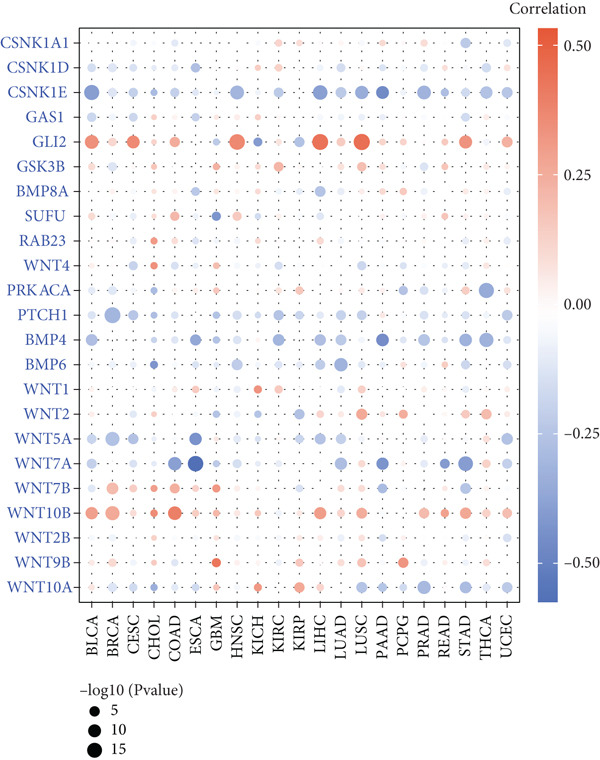
(f)

### 3.2. Construction of HH Signaling–Related Molecular Subtypes

We performed sample integration from the TCGA, GSE28735, GSE62452, and GSE57495 datasets and clustering analysis, determining the optimal clustering number *K* = 2 (Figures 3a, 3b, and 3c). All PC patients can be divided into two distinct molecular subtypes associated with HH signaling: HRGcluster A and HRGcluster B. PCA and t‐SNE demonstrated the reliability of our classification results (Figure 3d,e). Survival analysis demonstrated a notable difference in prognosis between PC patients assigned to HRGcluster A and HRGcluster B. Specifically, patients in HRGcluster A exhibited a significantly poorer prognosis compared to those in HRGcluster B (Figure 3f). This result emphasizes the possible clinical relevance of HH signaling in predicting patient outcomes and highlights the importance of further investigating the underlying molecular mechanisms associated with these distinct clusters in PC. Further comparison of clinical pathological features was conducted across different subtypes. Our analysis revealed that, in comparison to HRGcluster B, patients in HRGcluster A demonstrated a higher prevalence of advanced T and N stages, advanced clinical stages, and high pathological grades (Figures 3g, 3h, 3i, and 3j). This suggested that HRGcluster A could be linked to a more aggressive disease phenotype, underscoring the potential clinical significance of HH signaling in understanding the heterogeneity of the disease. Then, we investigated the expressed levels of 24 prognostic HRGs in different subtypes, showing a consistently poorer prognosis with the expression in HRGcluster A patients, where all 14 genes associated with poor prognosis are highly expressed in HRGcluster A (Figure 3k). Furthermore, ssGSEA indicated a higher HH signaling pathway score in HRGcluster A, suggesting a more active HH signaling connected with PC development and poor outcomes (Figure 3l).

Figure 3Construction of hedgehog (HH) signaling–related molecular subtypes. (a) The cumulative distribution function (CDF). (b) Changes in the area of CDF. (c) Consensus matrix. (d, e) PCA and t‐SNE analyses could clearly distinguish between patients with different HH signaling–related molecular subtypes. (f) Survival curves showed that the prognosis of HRGcluster A was worse than that of HRGcluster B. (g–j) The proportion of different T stages, N stages, clinical stages, and pathological grades between HRGcluster A and HRGcluster B. (k) Expression of hedgehog signaling‐related genes in HRGcluster A and HRGcluster B. (l) The ssGSEA indicated that the activity of the HH signaling pathway in HRGcluster A was higher (^ns^ *p* value > 0.05,  ^∗^ *p* value < 0.05,  ^∗∗^ *p* value < 0.01, and  ^∗∗∗^ *p* value < 0.001).

**Figure:**
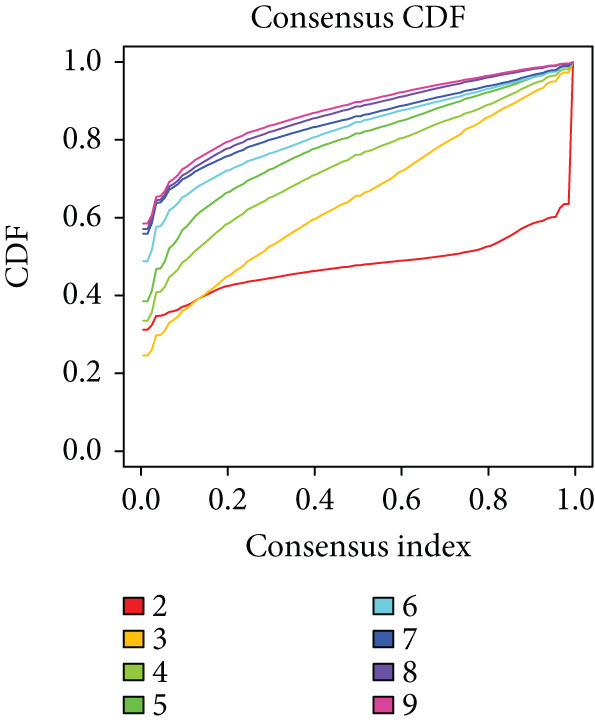
(a)

**Figure:**
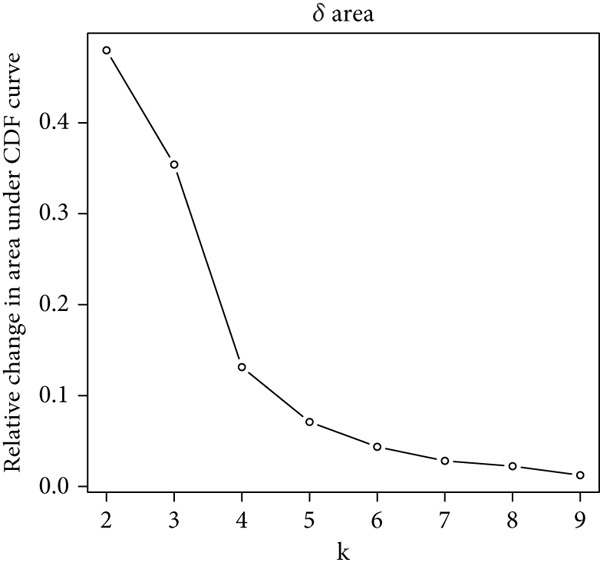
(b)

**Figure:**
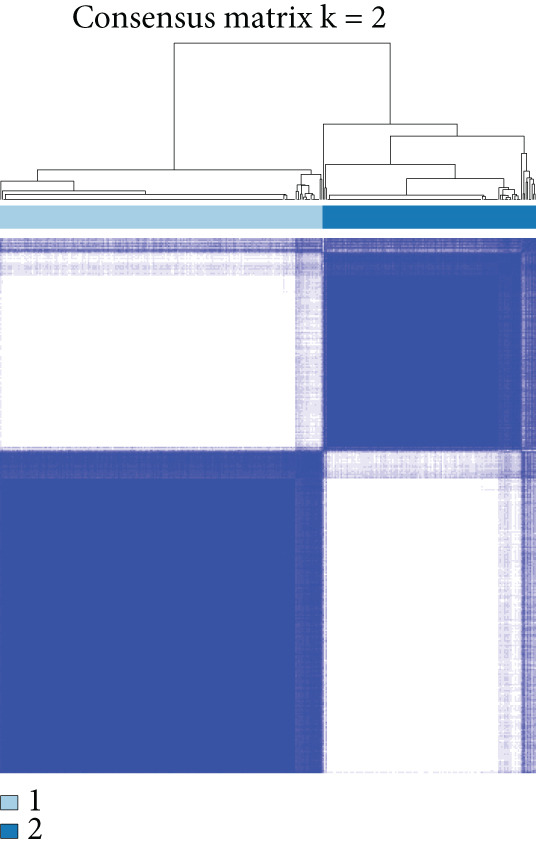
(c)

**Figure:**
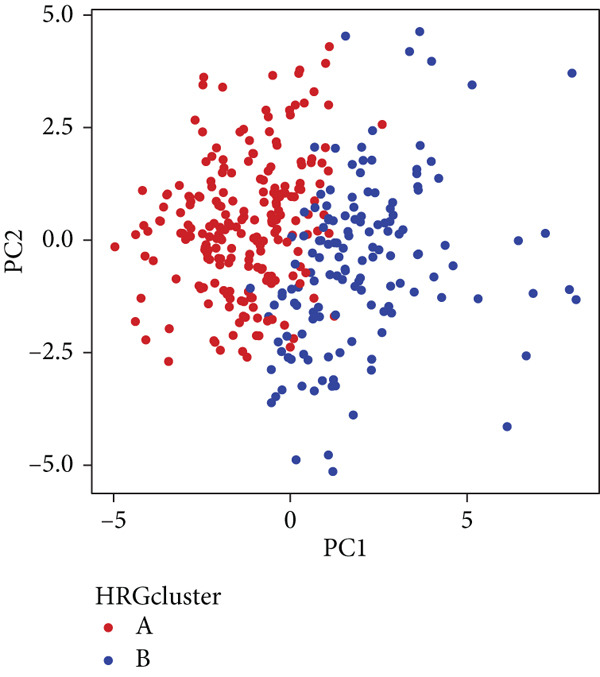
(d)

**Figure:**
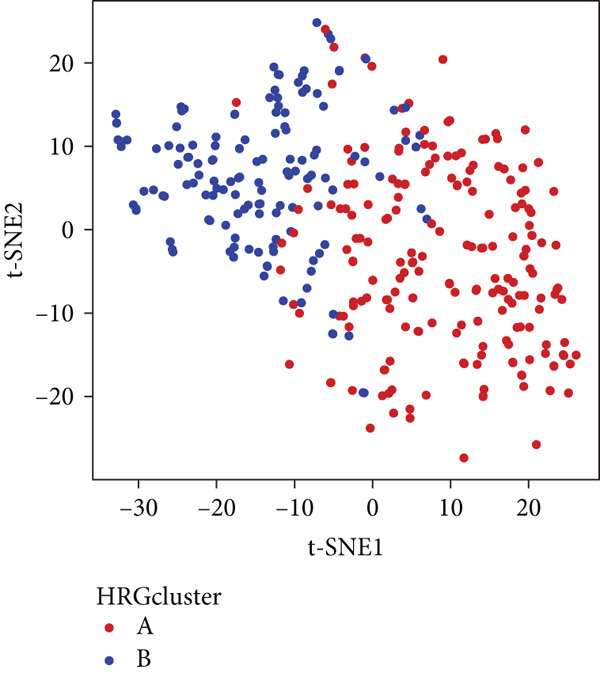
(e)

**Figure:**
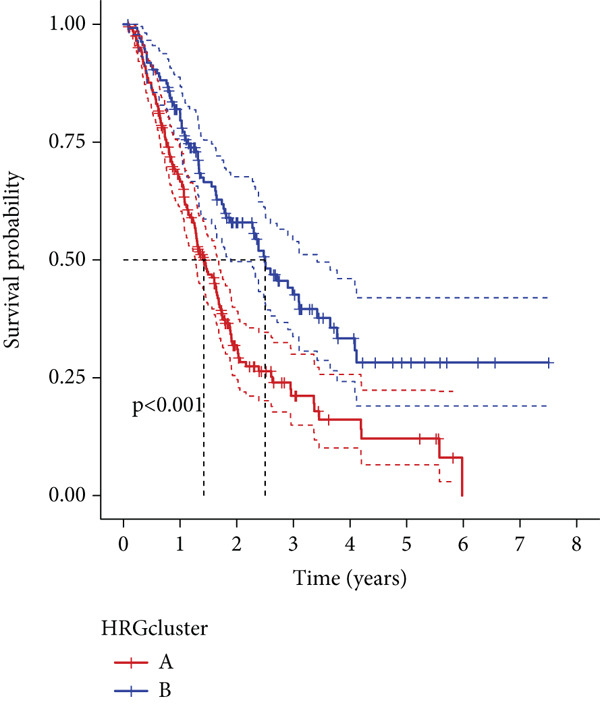
(f)

**Figure:**
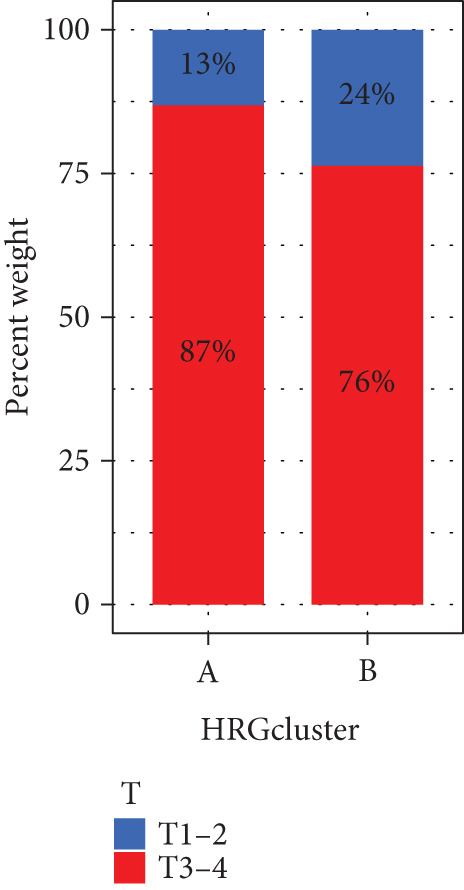
(g)

**Figure:**
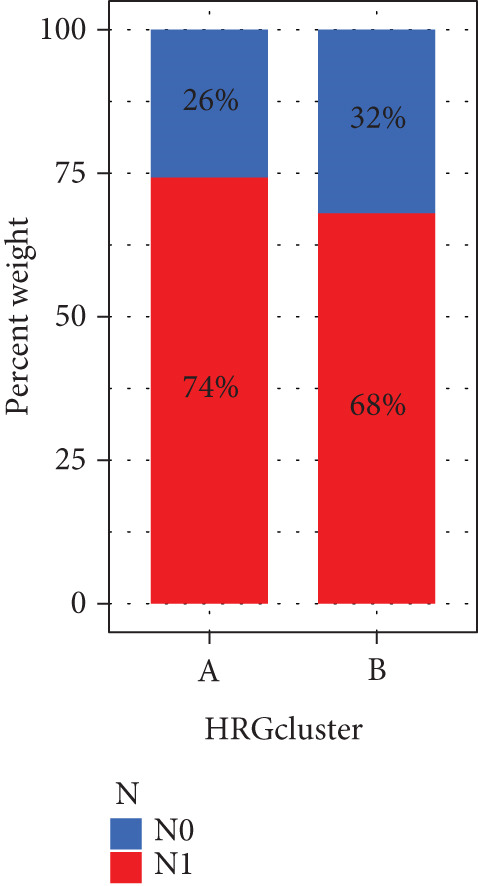
(h)

**Figure:**
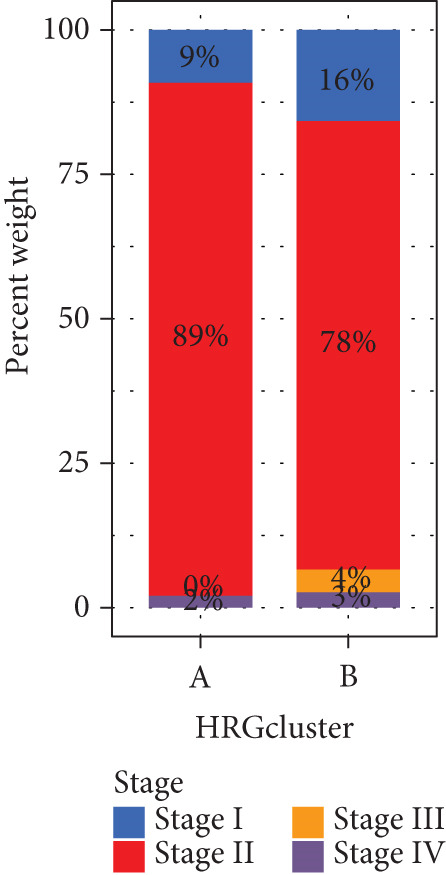
(i)

**Figure:**
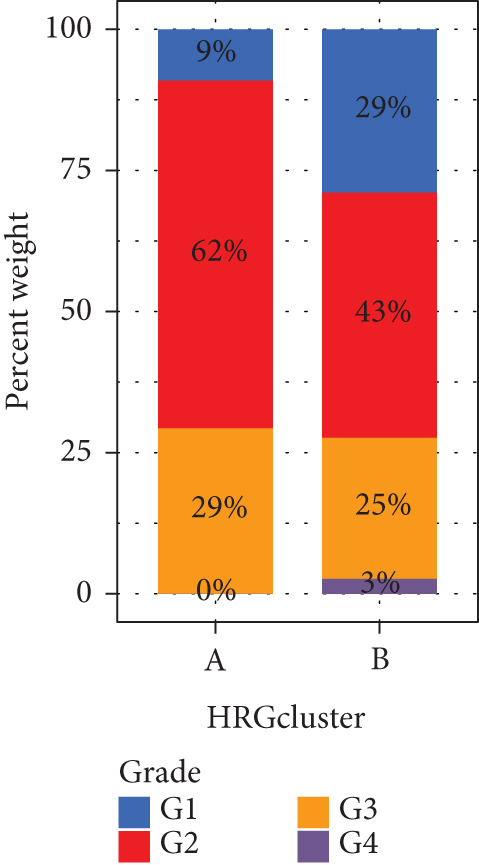
(j)

**Figure:**
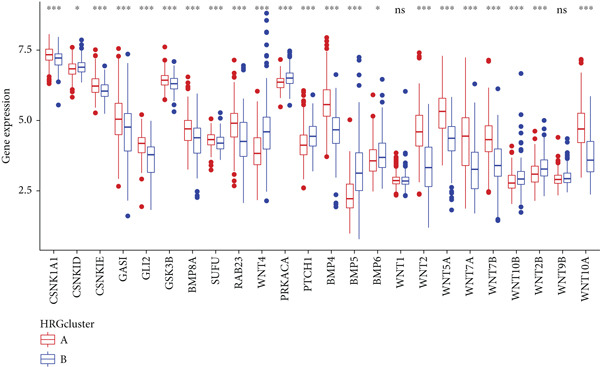
(k)

**Figure:**
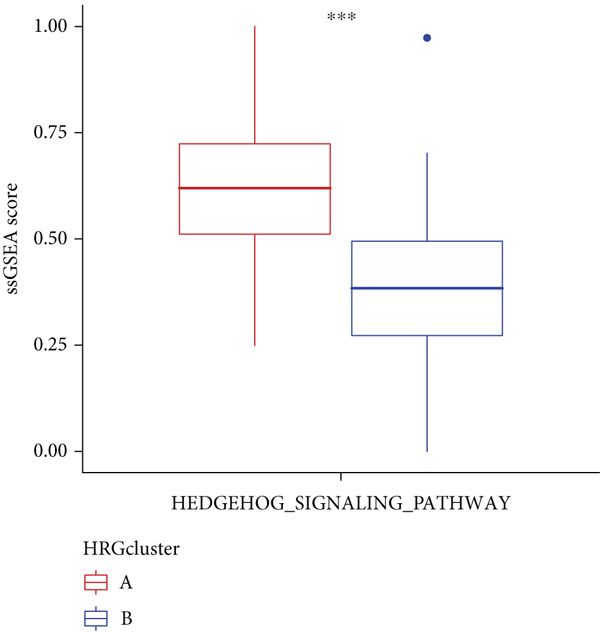
(l)

### 3.3. Biological Behavior, Immune Microenvironment, and Immunotherapy Response Between Different HH Signaling–Related Molecular Subtypes

To delve deeper into the underlying mechanisms contributing to the disparities observed between the two subtypes associated with the HH signaling pathway, we conducted GSVA and GSEA. GSVA results showed that HRGcluster B was mainly associated with intracellular energy metabolism, including “glycine serine threonine metabolism,” “primary bile acid biosynthesis,” “tryptophan metabolism,” “valine leucine isoleucine degradation,” and “fatty acid metabolism.” On the other hand, the enrichment patterns of HRGcluster A were more complex. Firstly, in contrast to the association of HRGcluster B with intracellular energy metabolism, HRGcluster A was significantly enriched in functions related to intercellular substance exchange and cell membrane alteration, such as “axon guidance,” “ECM receptor interaction,” “glycosphingolipid biosynthesis lacto and neolacto series,” and “glycosaminoglycan biosynthesis keratin sulfate.” Moreover, HRGcluster A was also closely associated with cancer development, as it was enriched in pathways like “basal cell carcinoma,” “small cell lung cancer,” and “P53 signaling pathway” (Figure 4a). These findings were further validated by GSEA. Of note, we observed a significant enrichment of the HH signaling pathway within HRGcluster A (Figure 4b), which is consistent with our previous findings using ssGSEA. These results provide further evidence supporting the potential association between aberrant HH signaling and the more aggressive disease phenotype observed in HRGcluster A.

Figure 4The biological behavior, immune microenvironment, and immunotherapy response between different hedgehog (HH) signaling‐related molecular subtypes. (a) GSVA. (b) GSEA. (c, d) The difference in immune cell subpopulation infiltration between HRGcluster A and HRGcluster B was evaluated by the ssGSEA and CIBERSORT algorithms. (e–h) The difference in T cell dysfunction, T cell exclusion, MSI, and TIDE scores between HRGcluster A and HRGcluster B (^ns^ *p* value > 0.05,  ^∗^ *p* value < 0.05,  ^∗∗^ *p* value < 0.01, and  ^∗∗∗^ *p* value < 0.001).

**Figure:**
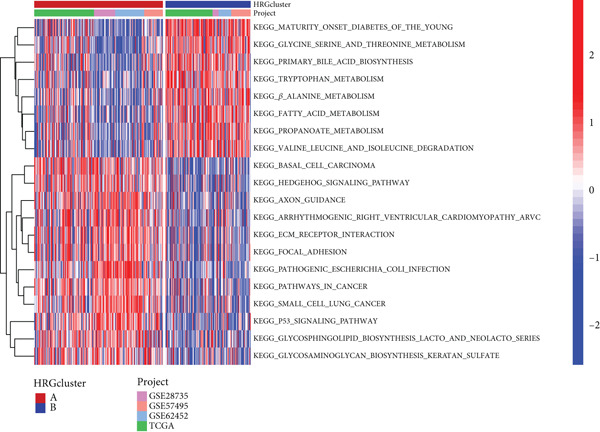
(a)

**Figure:**
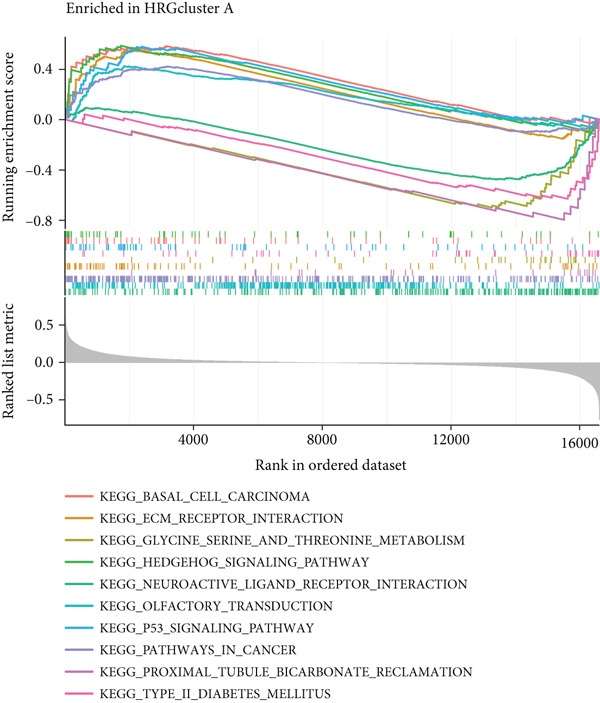
(b)

**Figure:**
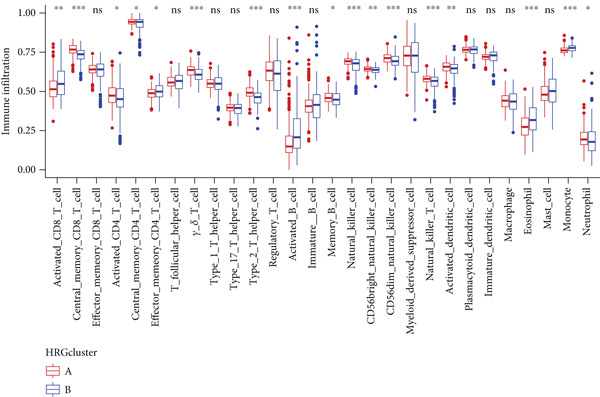
(c)

**Figure:**
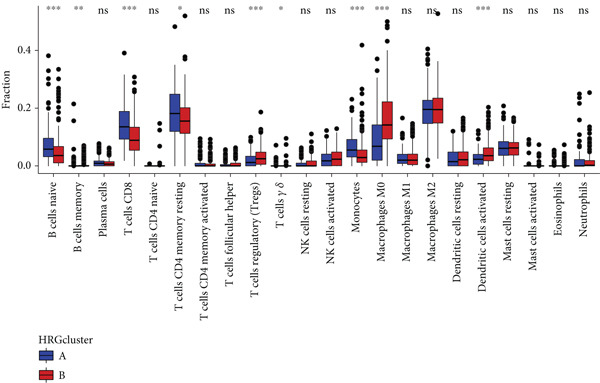
(d)

**Figure:**
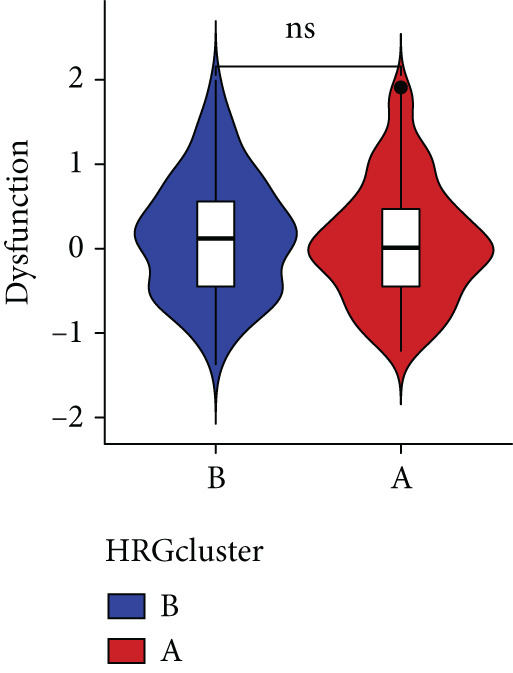
(e)

**Figure:**
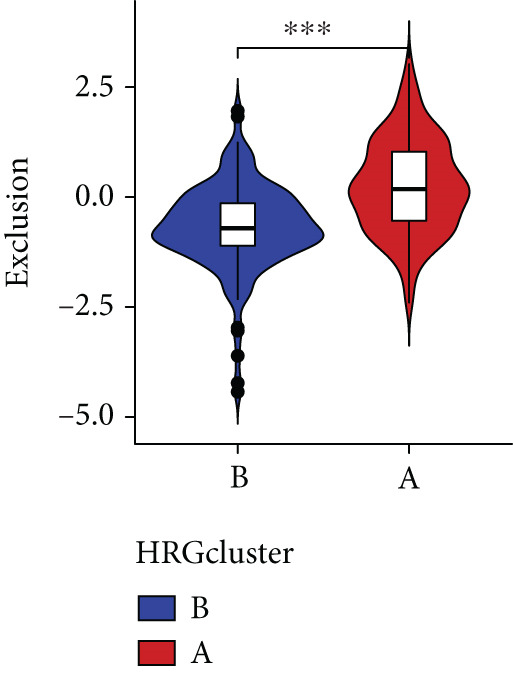
(f)

**Figure:**
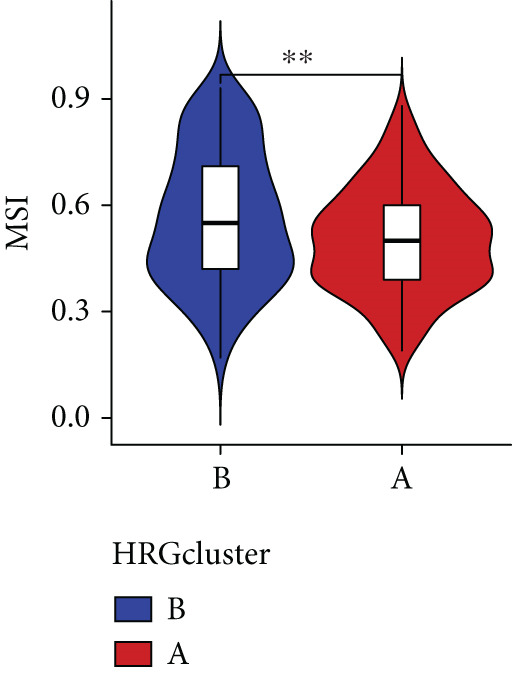
(g)

**Figure:**
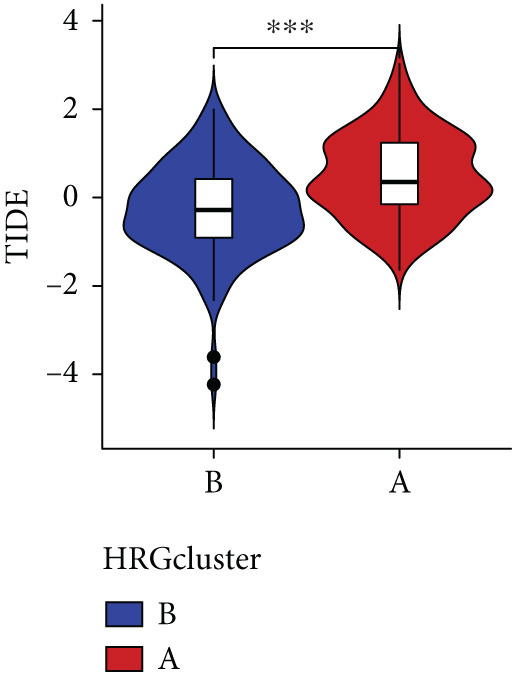
(h)

To assess the immunological characteristics between various subtypes, we employed the ssGSEA and CIBERSORT algorithms. The ssGSEA analysis revealed that HRGcluster A exhibited higher infiltration levels of activated CD4+ T cells, gamma delta T cells, natural killer cells (including CD56bright and CD56dim subsets), natural killer T cells, activated dendritic cells, and neutrophils. Conversely, HRGcluster B displayed notably higher abundances of activated CD8+ T cells, effector memory CD4+ T cells, activated B cells, eosinophils, and monocytes (Figure 4c). These findings shed light on the distinct immune microenvironments associated with each subtype and highlight potential differences in immune responses and tumor–immune interactions that may contribute to the contrasting clinical outcomes observed within the subtypes. Then, we employed the CIBERSORT algorithm to verify that HRGcluster A exhibited higher abundances of Tregs, M0 macrophages, activated dendritic cells, and gamma delta T cells, whereas HRGcluster B had abundances of naive B cells, CD8+ T cells, and monocytes (Figure 4d). These findings further support the notion that each subtype exhibits a unique immune microenvironment with distinct immune cell compositions. The identification of these differences may help guide personalized immunotherapeutic strategies for PC. Immunotherapy is aimed at enhancing the immune system′s ability to recognize and selectively target tumor cells, offering immense potential in the fight against cancer. By harnessing the natural power of the immune system, immunotherapy is likely to overcome the limitations of conventional cancer treatments as well as improve patient outcomes [43]. Through various mechanisms, such as immune checkpoint inhibition, adoptive cell transfer, or therapeutic vaccines, immunotherapy holds great promise for transforming the landscape of cancer treatment by providing more effective and targeted therapies [44]. PD‐1/PD‐L1 serves as a critical immune checkpoint that plays a pivotal role in regulating T cell responses. Activation of this pathway can trigger detrimental effects on T cell function, leading to apoptosis, dysfunction, and exhaustion. Consequently, the activation of PD‐1/PD‐L1 signaling hampers the activation, proliferation, and anticancer capabilities of CD8+ T cells, thereby facilitating the cancer immune reaction. By exploiting this mechanism, tumors can effectively evade the immune system′s surveillance and hinder the desired immune response against malignant cells [45]. Therefore, we further investigated the impact of the HH signal on immunotherapy response. Results showed that T cell dysfunction did not differ between different molecular subtypes, but HRGcluster A had higher T cell exclusion and TIDE scores, while HRGcluster B had a higher MSI score (Figures 4e, 4f, 4g, and 4h). These results suggested that HRGcluster B might be more likely to benefit from immunotherapy.

### 3.4. HH Signaling–Related Molecular Subtypes Are Associated With Sensitivity to Chemotherapy and Targeted Therapy

In the management of PC, drug‐assisted therapy, particularly chemotherapy, plays a crucial role in improving patient prognosis. However, PC is characterized by its intricate nature, displaying diverse genetic and phenotypic variations among individuals. Consequently, determining the specific drug sensitivity of each patient becomes instrumental in making informed clinical decisions regarding personalized treatment approaches. In this study, we employed the “OncoPredict” package to evaluate variances in drug sensitivity across various molecular subtypes of PC patients. Results showed that PC patients within HRGcluster B subtype were more sensitive to 5‐fluorouracil, gemcitabine, cisplatin, erlotinib, irinotecan, KRAS(G12C) inhibitor, oxaliplatin, cyclophosphamide, paclitaxel, and sorafenib (Figures 5a, 5b, 5c, 5d, 5e, 5f, 5g, 5g, 5h, 5i, and 5k). PC patients within HRGcluster A subtype exhibited enhanced sensitivity towards selumetinib and trametinib (Figure 5j,l).

Figure 5Sensitivity to chemotherapy and targeted therapy between different hedgehog (HH) signaling–related molecular subtypes. PC patients with HRGcluster B had higher sensitivity to 5‐fluorouracil (a), cisplatin (b), erlotinib (c), gemcitabine (d), irinotecan (e), KRAS(G12C) inhibitor (f), oxaliplatin (g), cyclophosphamide (h), paclitaxel (i), and sorafenib (k). PC patients in the HRGcluster A group had higher sensitivity to selumetinib (j) and trametinib (l).

**Figure:**
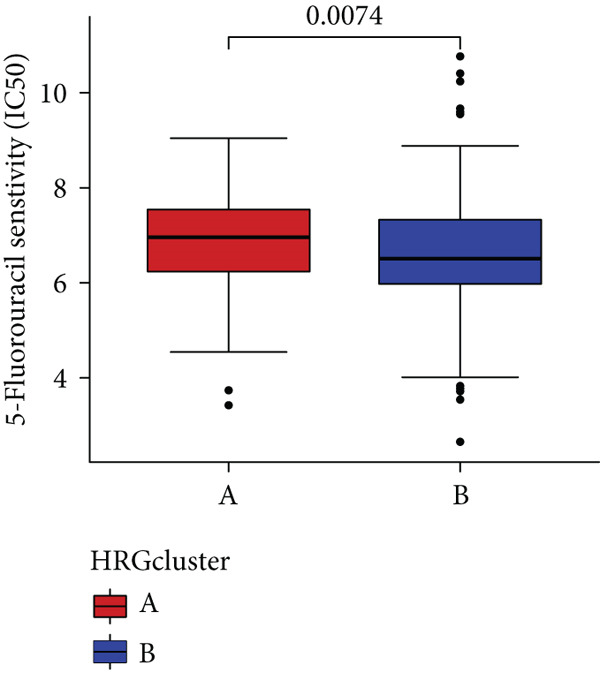
(a)

**Figure:**
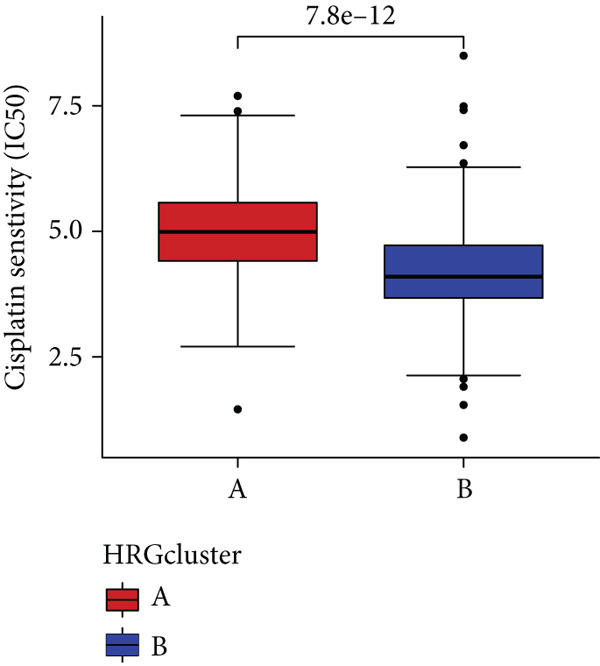
(b)

**Figure:**
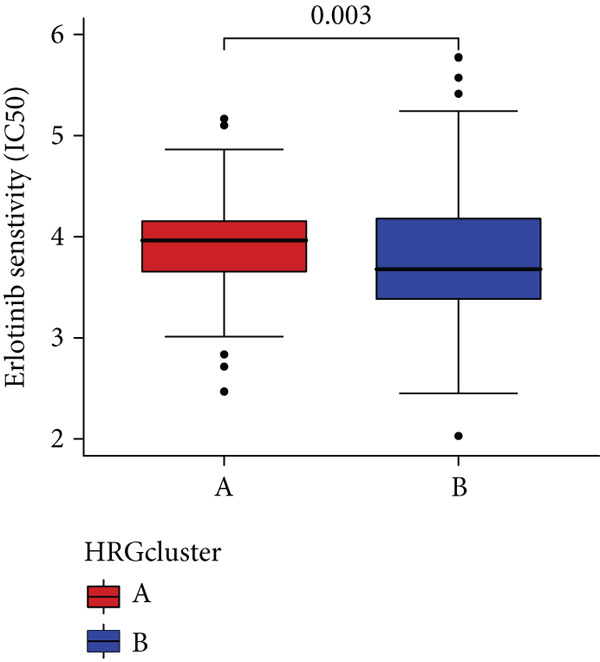
(c)

**Figure:**
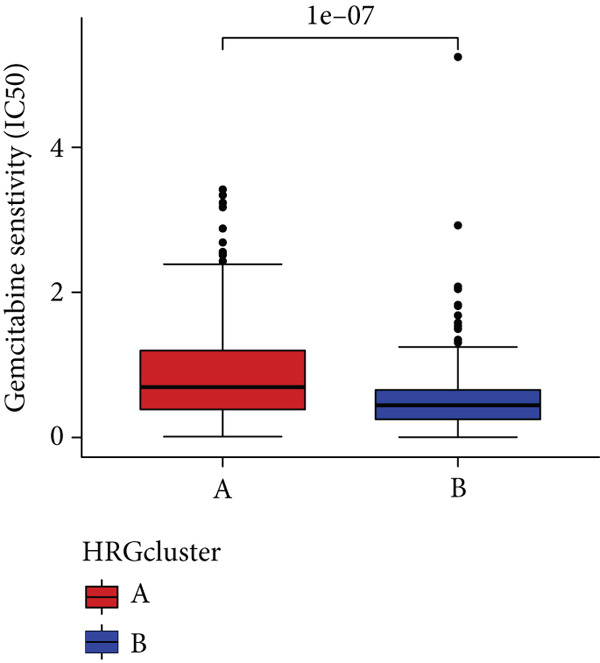
(d)

**Figure:**
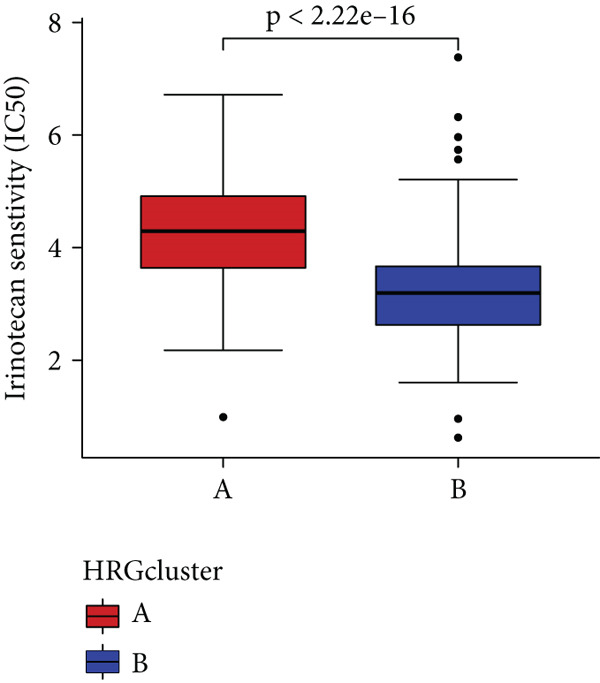
(e)

**Figure:**
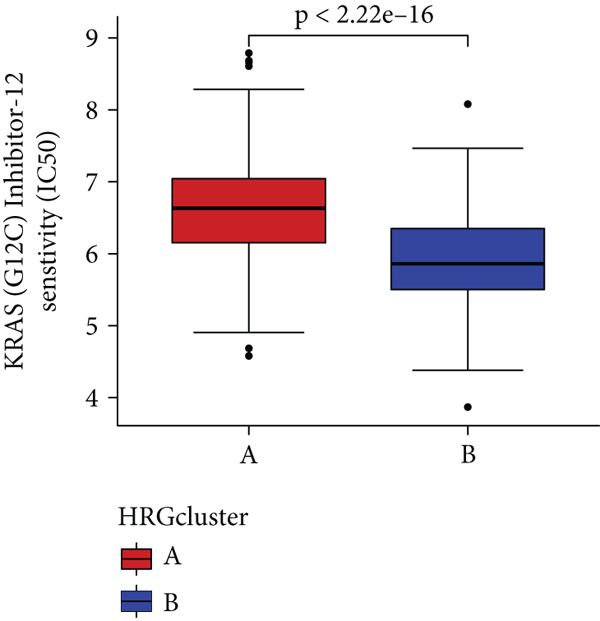
(f)

**Figure:**
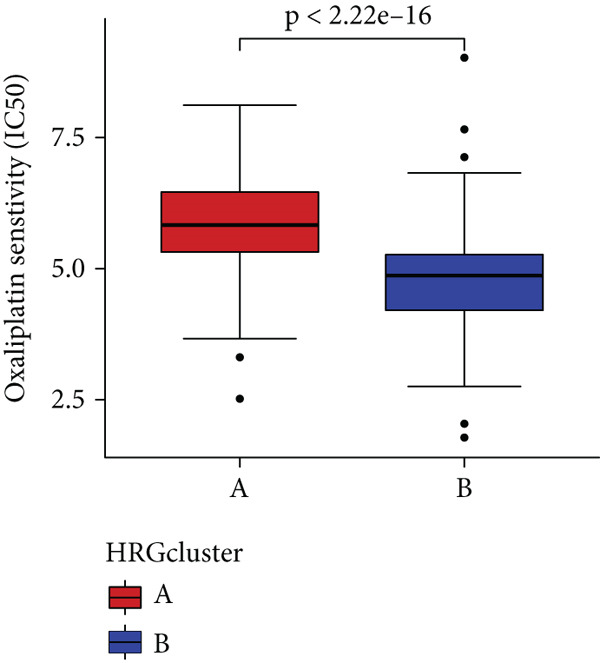
(g)

**Figure:**
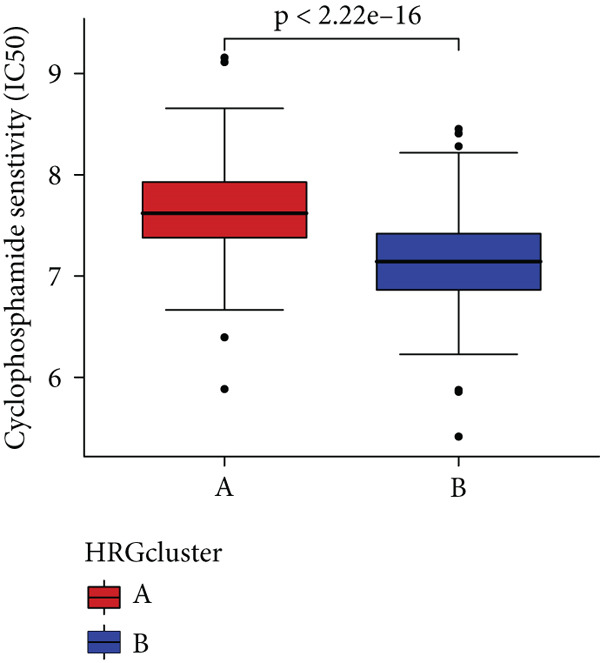
(h)

**Figure:**
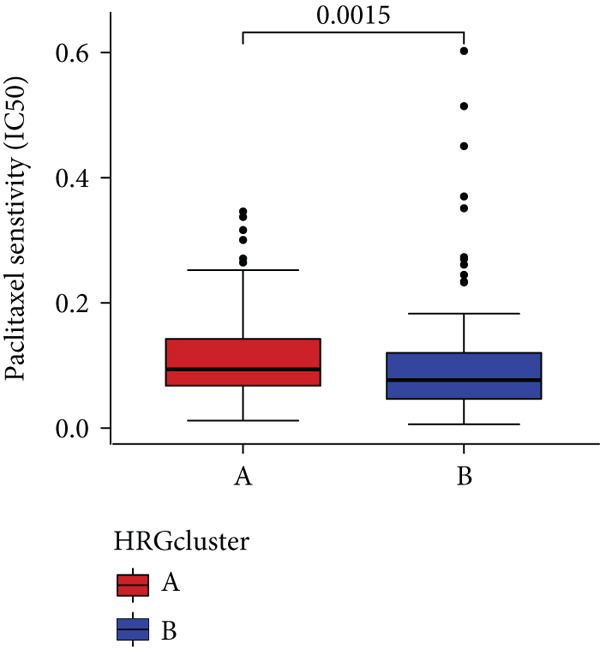
(i)

**Figure:**
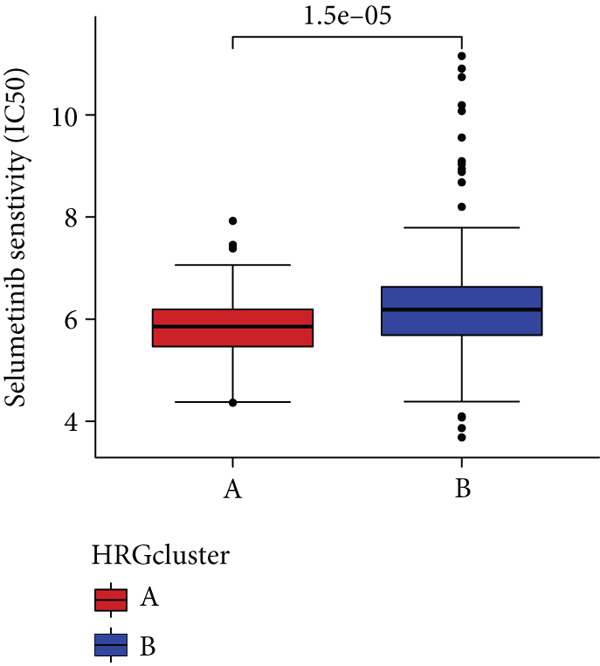
(j)

**Figure:**
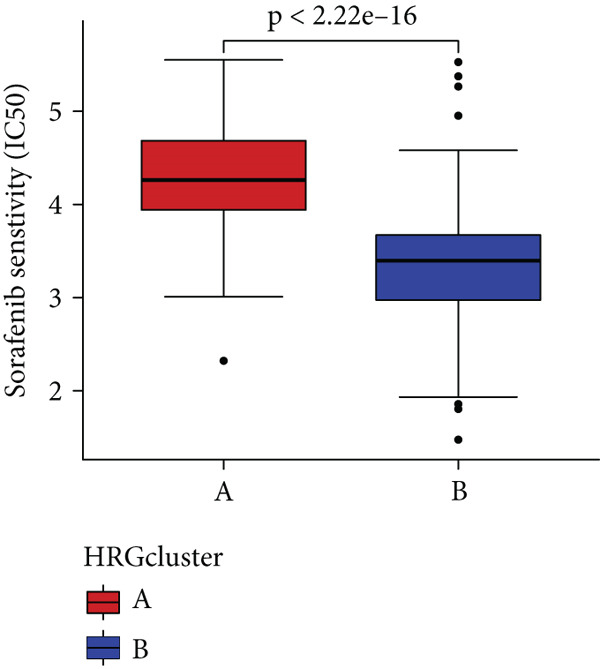
(k)

**Figure:**
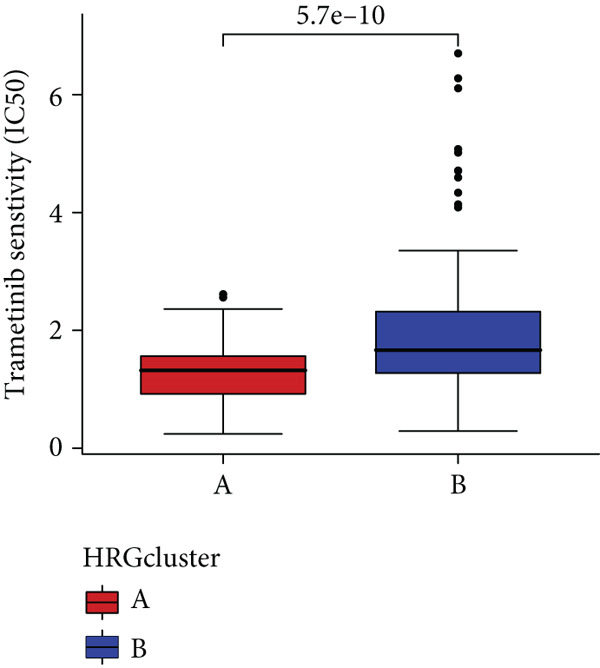
(l)

### 3.5. Differential Expression Analysis, Enrichment Analyses, and Clustering Analysis

To gain deeper insights into the distinctions among various patterns of HH signaling, we conducted a differential expression analysis. By examining the gene expression profiles, we elucidated the molecular mechanisms that underlie the divergent outcomes of HH signaling activation. Through this approach, we aimed to uncover the regulatory nodes that govern the fine‐tuning of HH signaling and the downstream effectors that contribute to the observed heterogeneity. A total of 258 HRDEGs were obtained, with 158 highly expressed in HRGcluster A and 100 expressed in HRGcluster B (Figure 6a). GO enrichment analysis indicated significant enrichment of these HRDEGs in processes like “extracellular structure organization,” “extracellular matrix organization,” “epithelial cell proliferation,” and other relevant aspects (Figure 6b). KEGG enrichment analysis uncovered that these genes exhibited potential involvement in functions related to “ECM‐receptor interaction,” “pancreatic secretion,” “focal adhesion,” “human papillomavirus infection,” and other pertinent pathways (Figure 6c). To demonstrate the influence of HRDEGs on the prognosis of PC, we conducted consensus clustering. As a result, all PC patients were categorized into two distinct subtypes: HRDEGcluster A and HRDEGcluster B (Figures 6d, 6e, and 6f). PCA and t‐SNE techniques could effectively distinguish patients from HRDEGcluster A and HRDEGcluster B, underscoring the robustness and reliability of the clustering outcomes (Figure 6g,h). Survival analysis revealed a significantly superior prognosis for patients in HRDEGcluster B compared to those in HRDEGcluster A (Figure 6i).

Figure 6Differential expression analysis, enrichment analyses, and clustering analysis. (a) 258 differentially expressed genes were identified between different hedgehog (HH) signaling–related subtypes. (b) GO enrichment analysis. (c) KEGG enrichment analysis. (d) The cumulative distribution function (CDF). (e) Changes in the area of CDF. (f) Consensus matrix. (g, h) PCA and t‐SNE analyses could clearly distinguish HRDEGcluster A from HRDEGcluster B. (i) Survival curves showed that the prognosis of HRDEGcluster A was worse than that of HRDEGcluster B.

**Figure:**
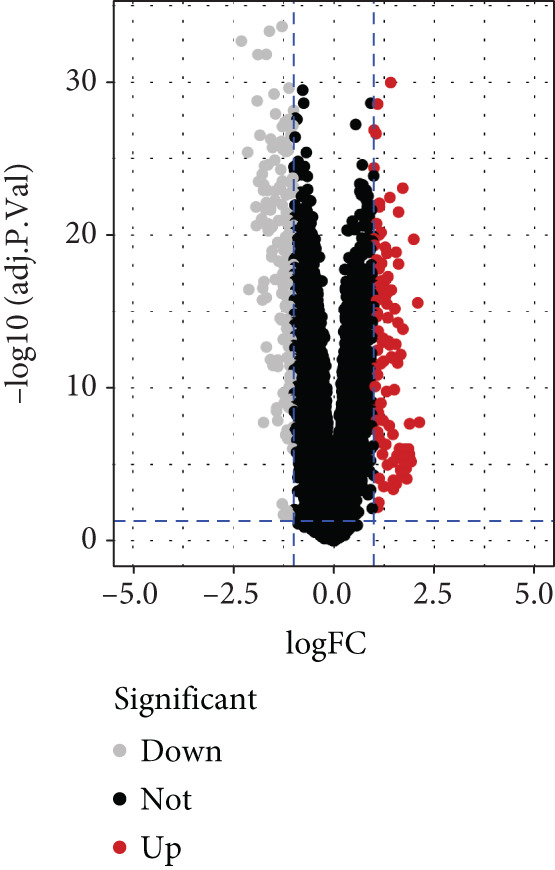
(a)

**Figure:**
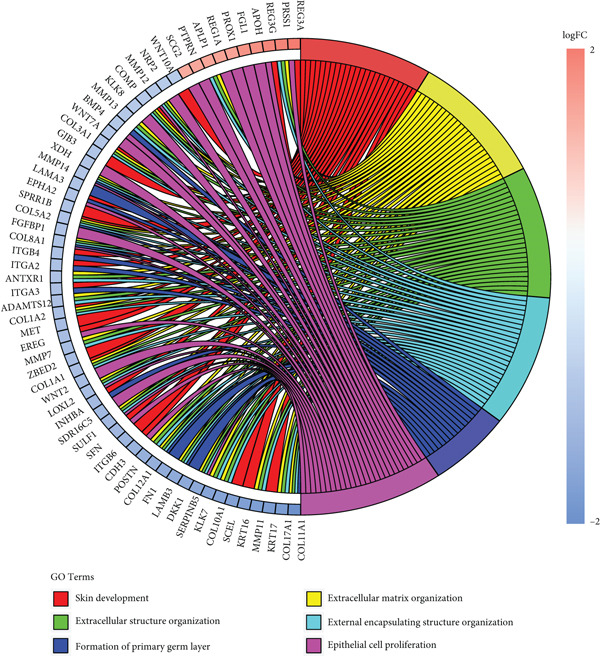
(b)

**Figure:**
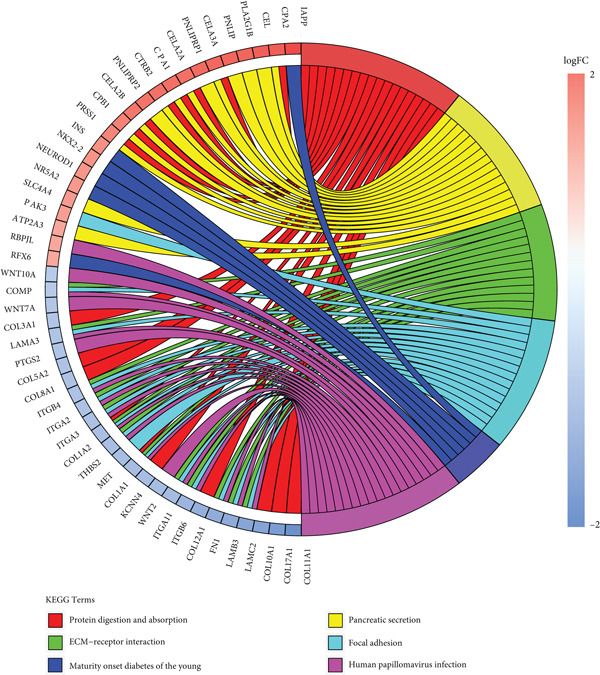
(c)

**Figure:**
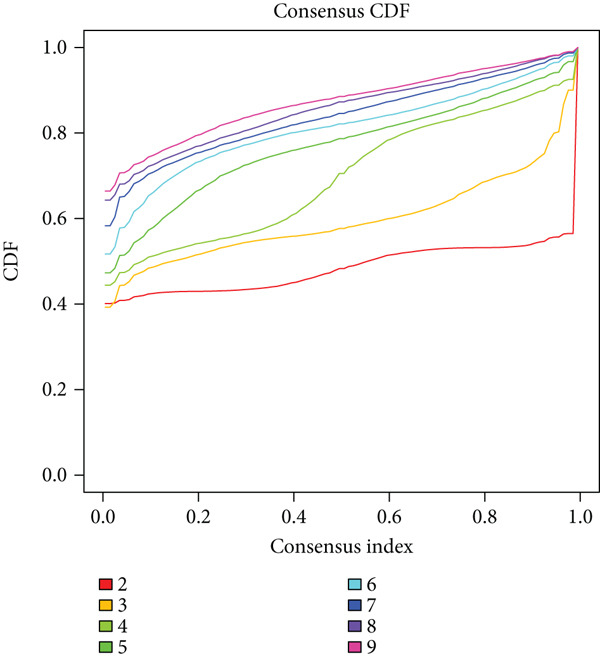
(d)

**Figure:**
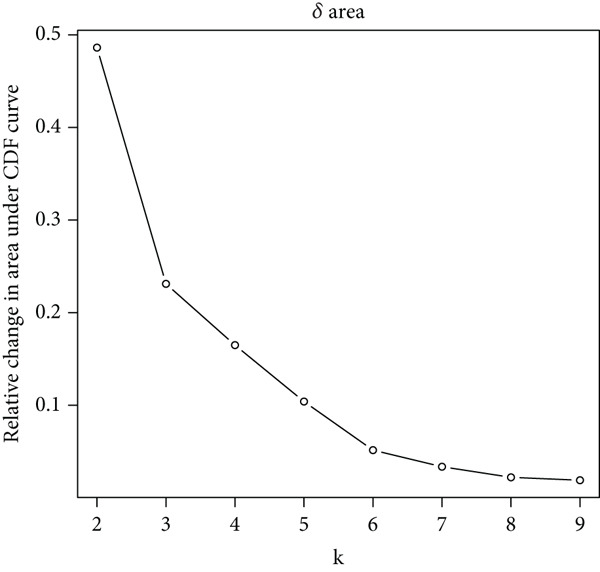
(e)

**Figure:**
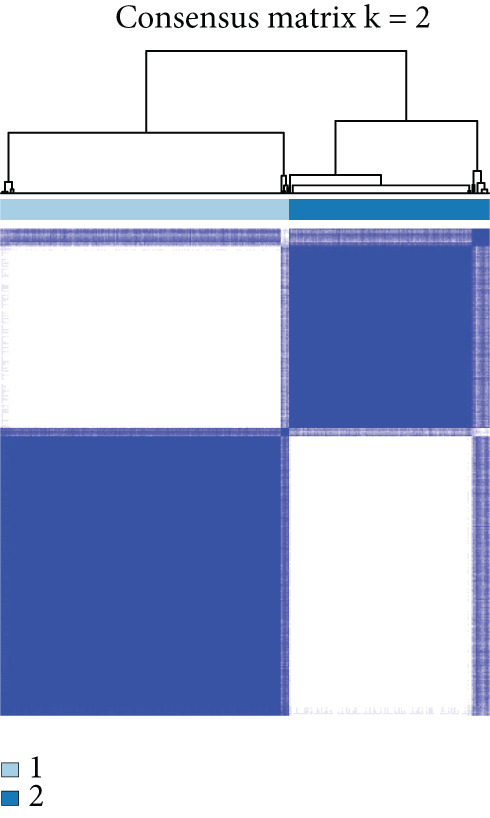
(f)

**Figure:**
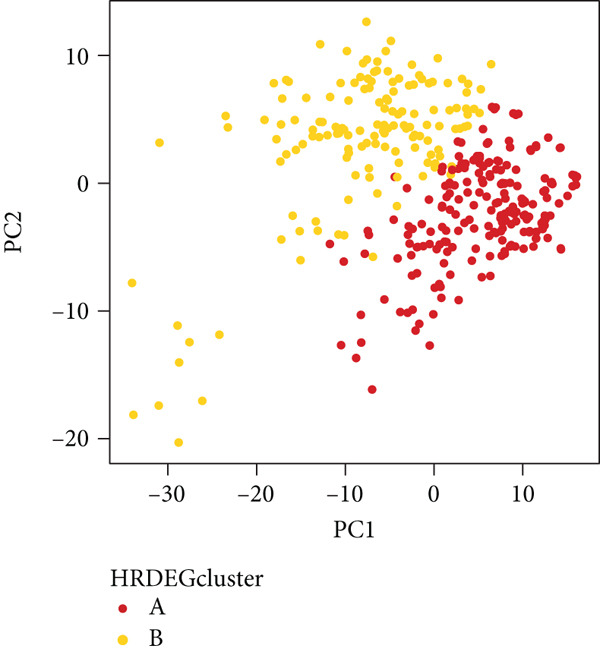
(g)

**Figure:**
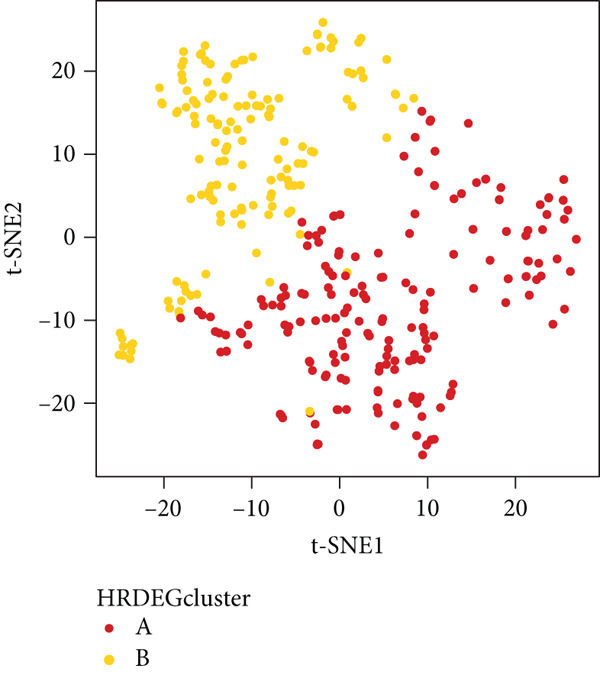
(h)

**Figure:**
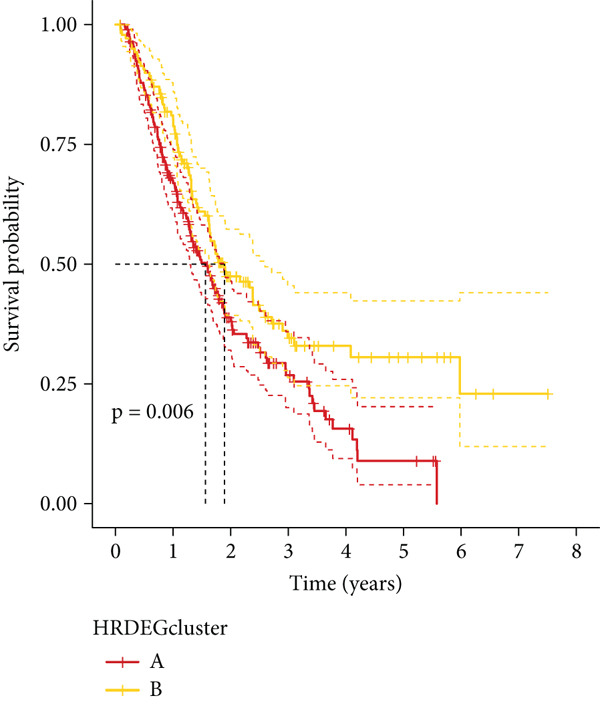
(i)

### 3.6. Building and Validation of Prognostic Model

To enhance the predictive accuracy of individual sample outcomes, we employed LASSO and Cox regression to develop a prognostic model for PC utilizing HRDEGs. Initially, we conducted univariable Cox regression analysis to screen 68 prognosis‐linked HRDEGs (Supporting Information 2: Table S3). Then, LASSO regression was implemented to eliminate overfitting among prognosis‐linked HRDEGs (Figure 7a,b). Then, multivariate Cox regression analysis was used to further screen prognosis‐linked HRDEGs and construct a prognostic model, which includes four genes: SERPINB3, LY6D, DCBLD2, and ANLN (Figure 7c). KM curves manifested that, compared with high‐risk PC, low‐risk PC had a notably longer survival time (Figure 7d). The internal validation and whole TCGA‐PC cohorts further confirmed the reliability of the model (Figure 7e,f). AUC for 1‐year, 2‐year, and 3‐year survival rates in the training cohort were 0.785, 0.800, and 0.770, respectively (Figure 7g), while in the internal validation cohort, they were 0.723, 0.627, and 0.708, respectively (Figure 7h), and in the whole TCGA‐PC cohort, they were 0.757, 0.707, and 0.739, respectively (Figure 7i), indicating that our model has good predictive value. PCA and t‐SNE further confirmed the rationality of the model (Figures 7j, 7k, and 7l). What is more, to further validate the model′s reliability, we used samples from the GSE28735, GSE57495, and GSE62452 datasets for external validation, and the results further confirmed the reliability of our model. KM curves manifested that high‐risk PC had poorer outcomes (Figures 8a, 8b, and 8c). AUC for 1‐year, 2‐year, and 3‐year survival rates in the GSE28735 dataset were 0.601, 0.769, and 0.887, respectively (Figure 8d); in the GSE57495 dataset, they were 0.694, 0.697, and 0.689, respectively (Figure 8e); in the GSE62452 dataset, they were 0.589, 0.749, and 0.883, respectively (Figure 8f). PCA and t‐SNE again confirmed the model′s reliability (Figures 8g, 8h, and 8i).

Figure 7Establishment and verification of hedgehog (HH) signaling–related prognostic model. (a) The coefficient path of LASSO regression. (b) The cross‐validation curve. (c) Coefficient of SERPINB3, LY6D, DCBLD2, and ANLN. (d) Survival curves of the training, (e) internal validation, and (f) whole TCGA‐PC cohorts showed that the low‐risk group had a better prognosis. ROC curves of the (g) training, (h) internal validation, and (i) whole TCGA‐PC cohorts indicated that the HH signaling–related prognostic model had excellent prediction performance. PCA and t‐SNE analyses could clearly distinguish PC patients with various risk scores in the (j) training, (k) internal validation, and (l) whole TCGA‐PC cohorts.

**Figure:**
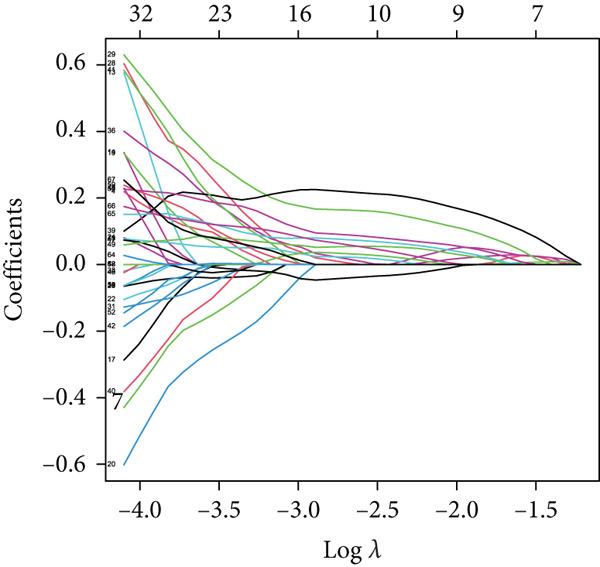
(a)

**Figure:**
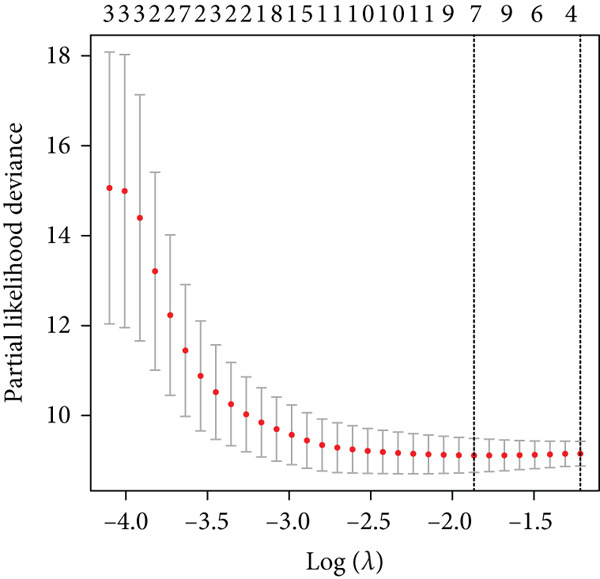
(b)

**Figure:**
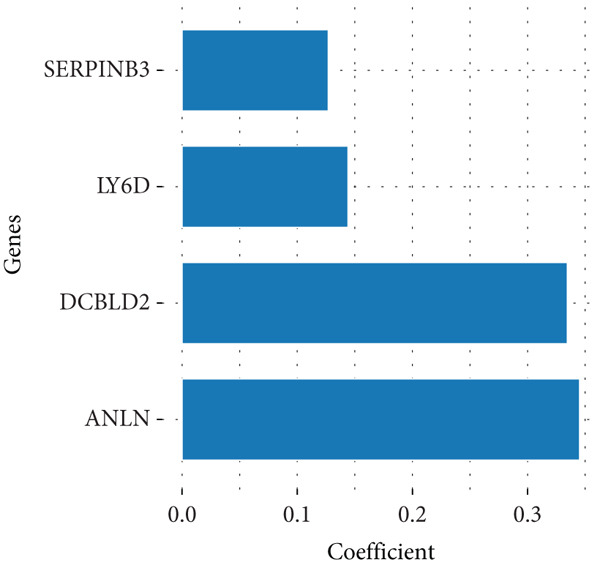
(c)

**Figure:**
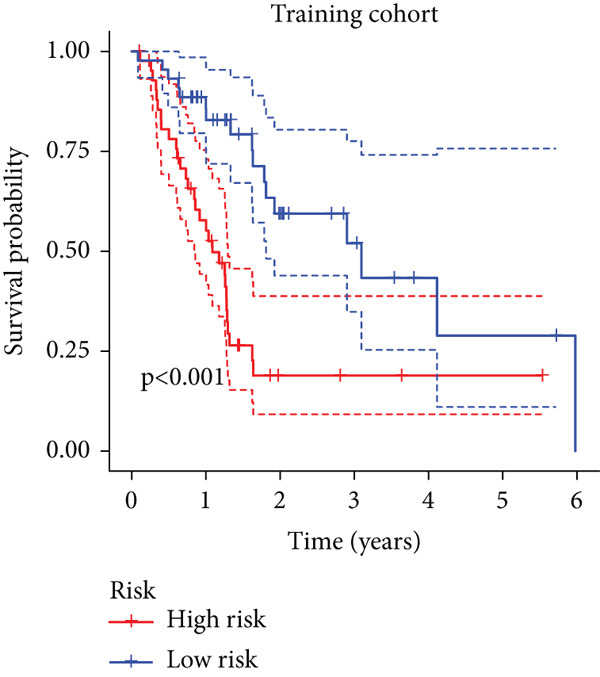
(d)

**Figure:**
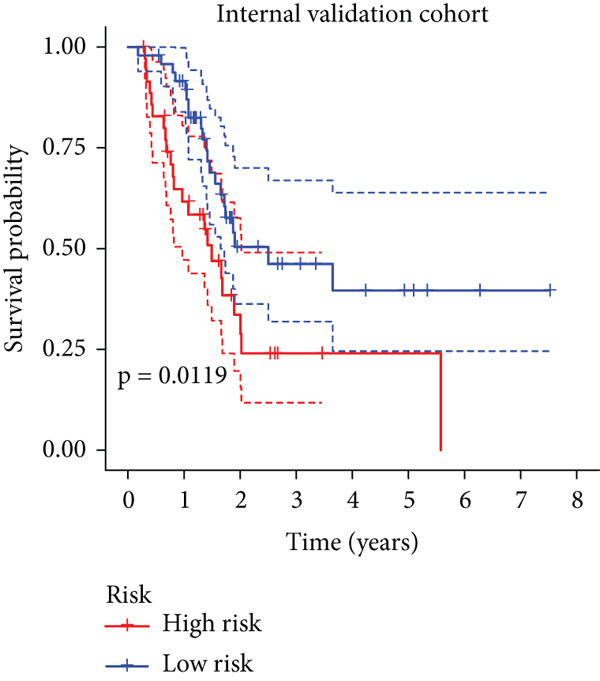
(e)

**Figure:**
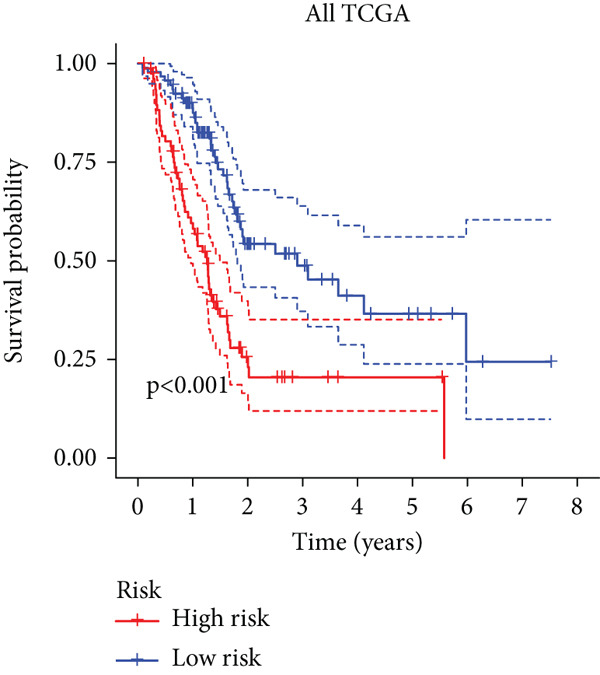
(f)

**Figure:**
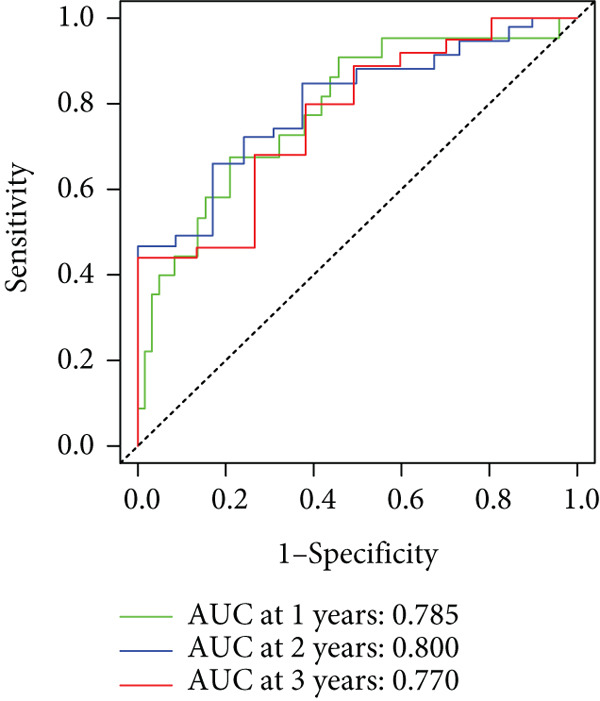
(g)

**Figure:**
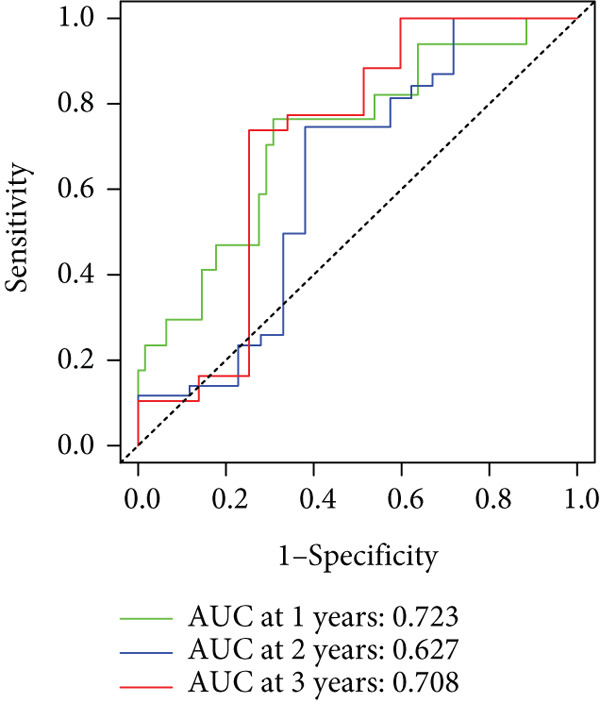
(h)

**Figure:**
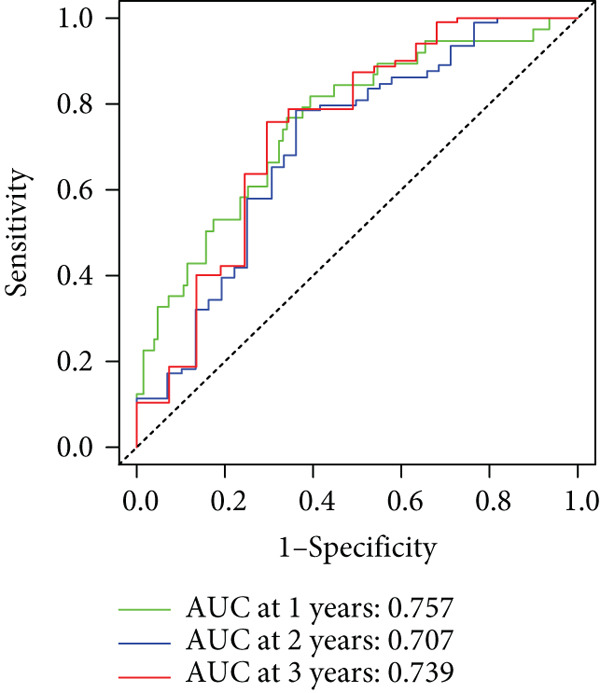
(i)

**Figure:**
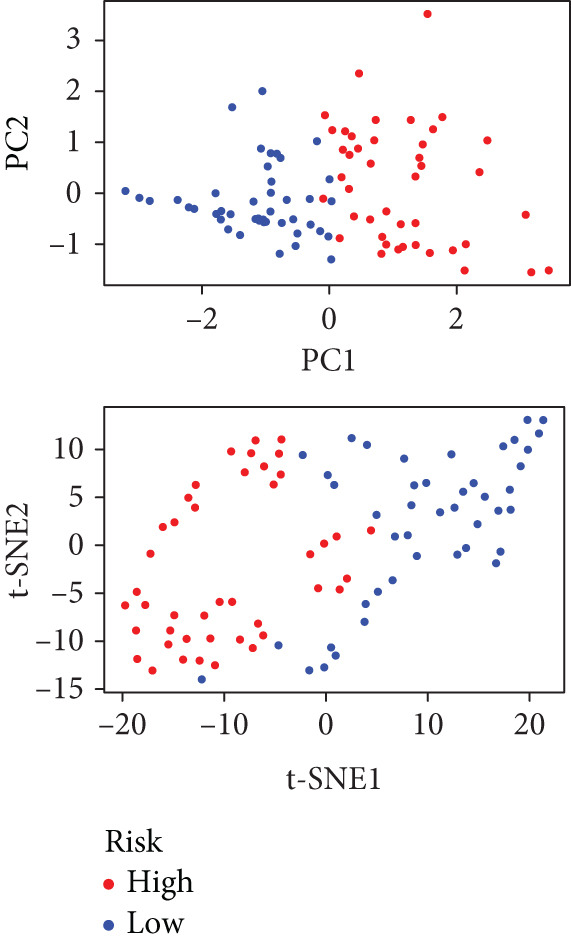
(j)

**Figure:**
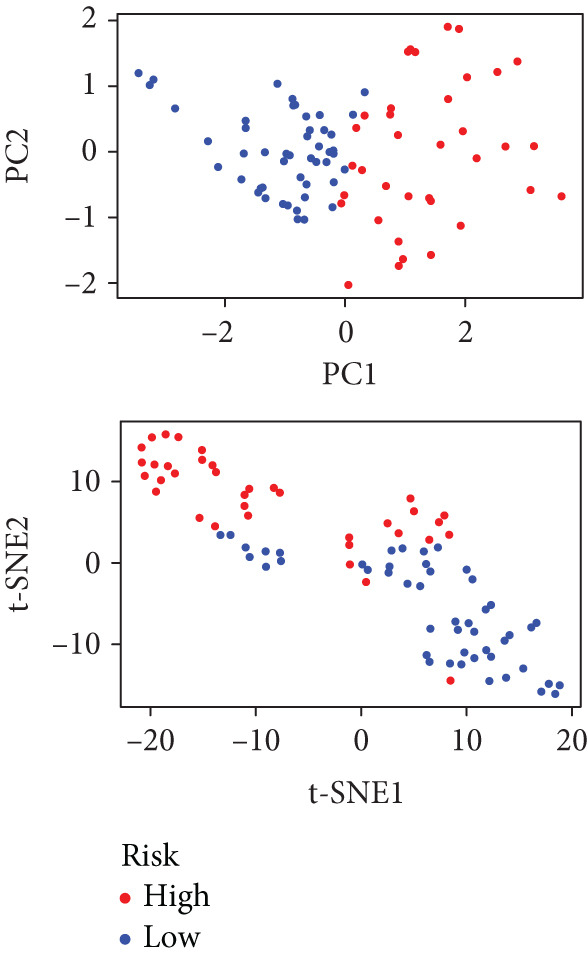
(k)

**Figure:**
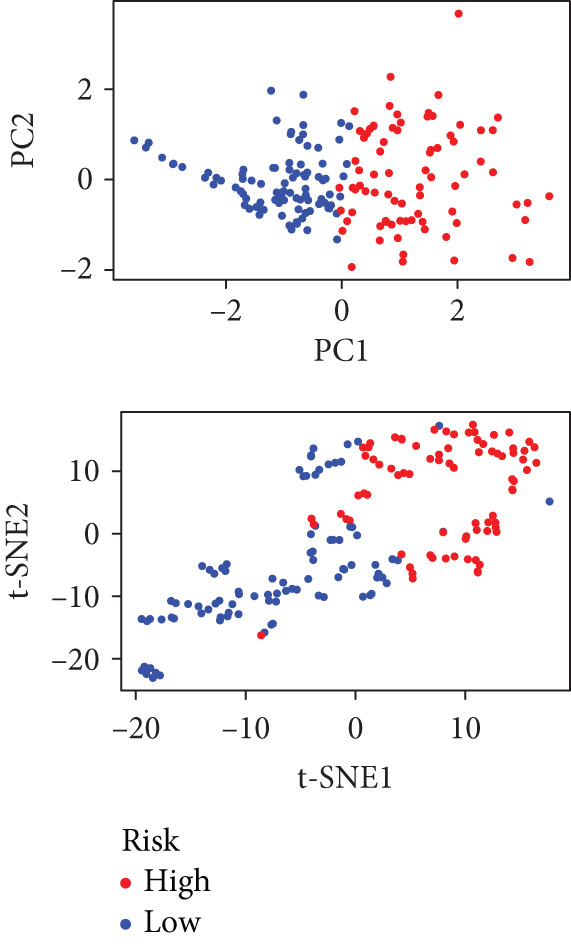
(l)

Figure 8External validation of the hedgehog (HH) signaling–related prognostic model. Survival curves of the GSE28735 (a), GSE57495 (b), and GSE62452 (c) cohorts validated that PC patients with a low risk had a better prognosis. ROC curves of the GSE28735 (d), GSE57495 (e), and GSE62452 (f) cohorts validated that the HH signaling–related prognostic model had excellent prediction performance. PCA and t‐SNE analyses could clearly distinguish PC patients with various risk scores in the GSE28735 (g), GSE57495 (h), and GSE62452 (i) cohorts.

**Figure:**
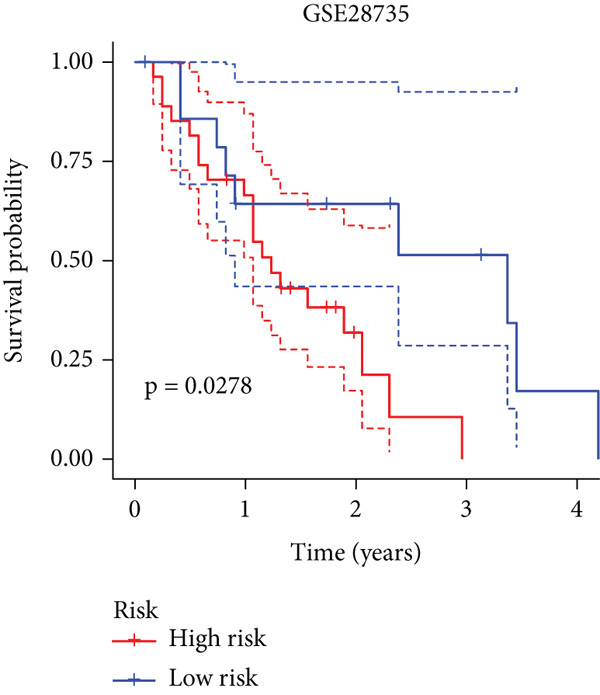
(a)

**Figure:**
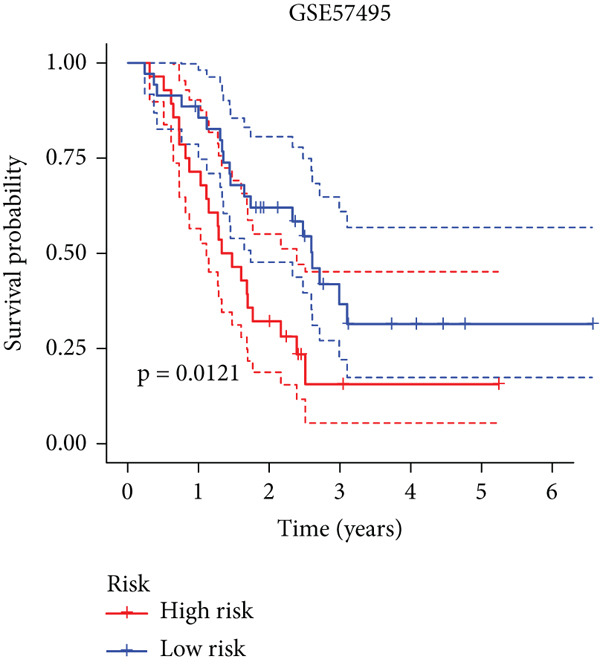
(b)

**Figure:**
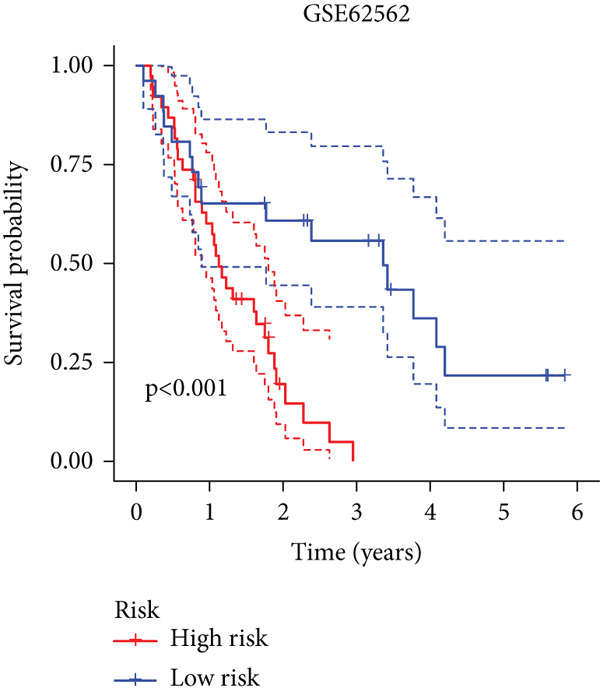
(c)

**Figure:**
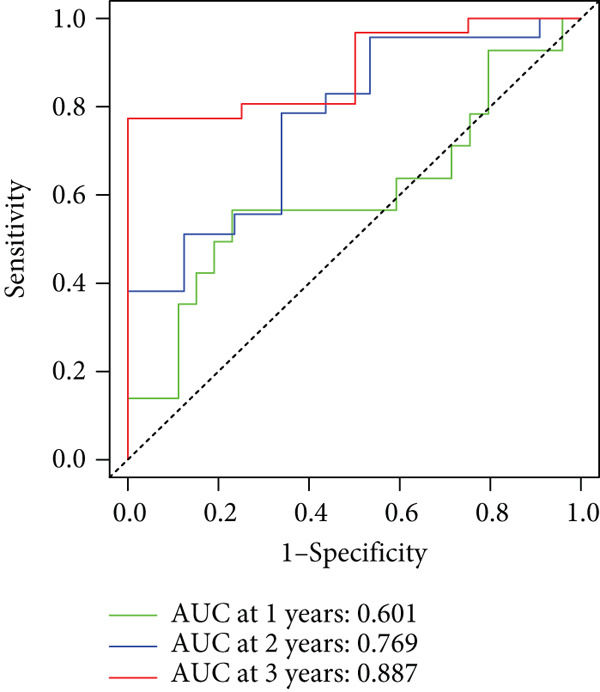
(d)

**Figure:**
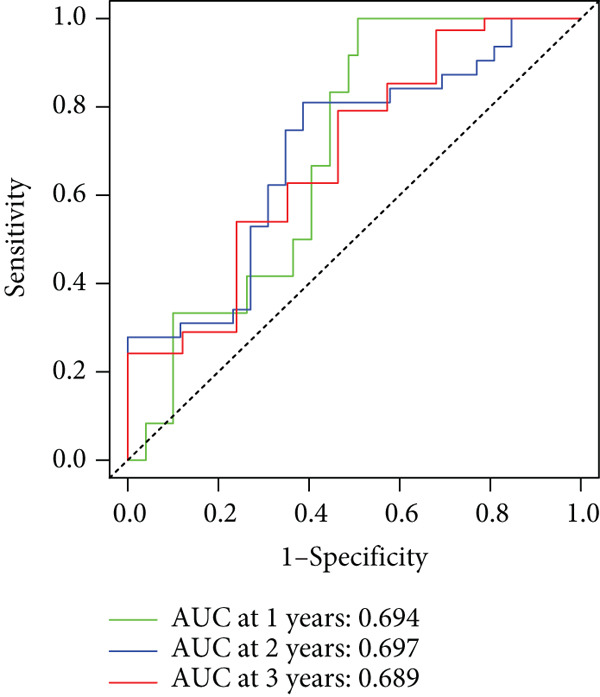
(e)

**Figure:**
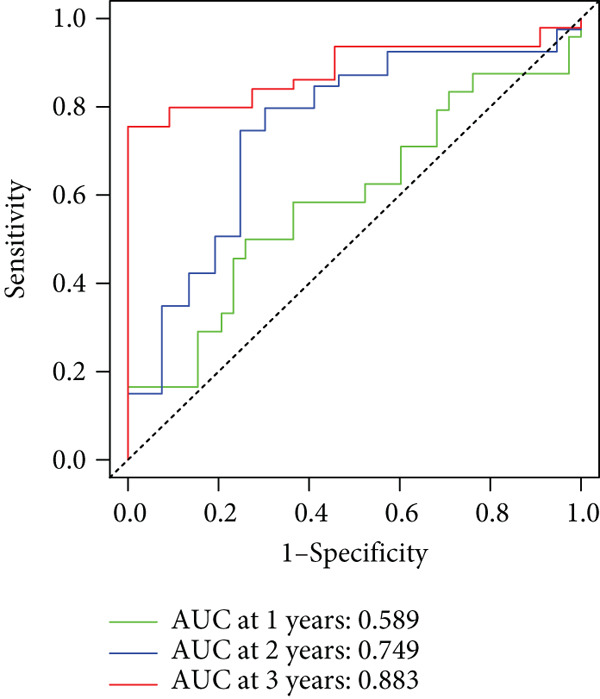
(f)

**Figure:**
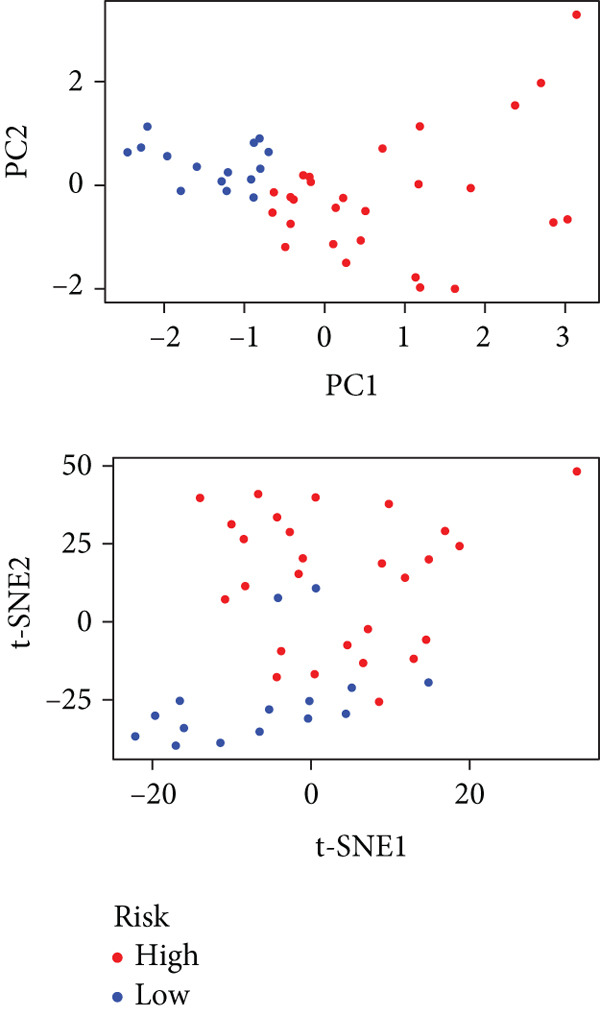
(g)

**Figure:**
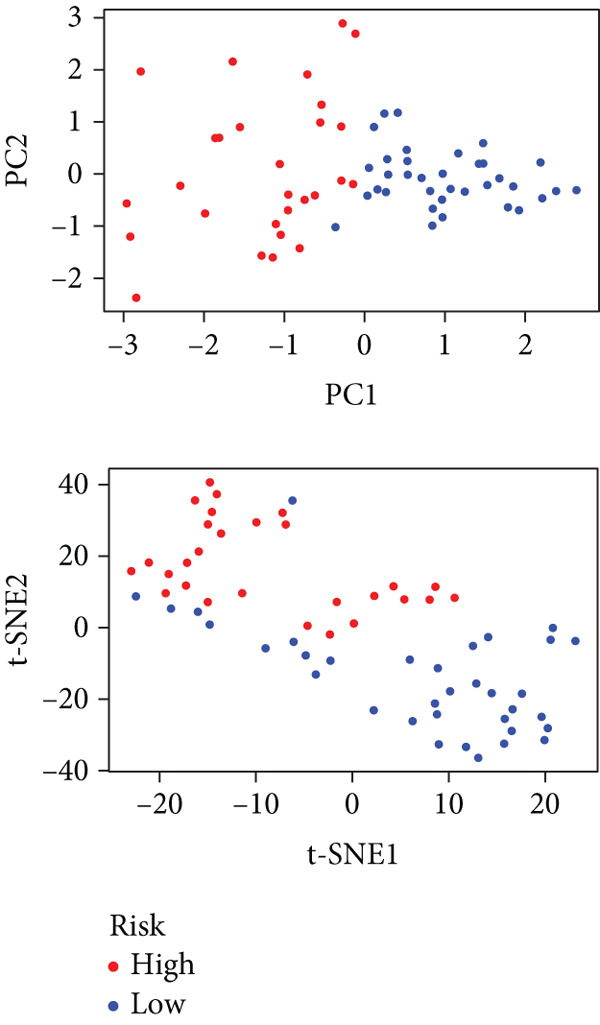
(h)

**Figure:**
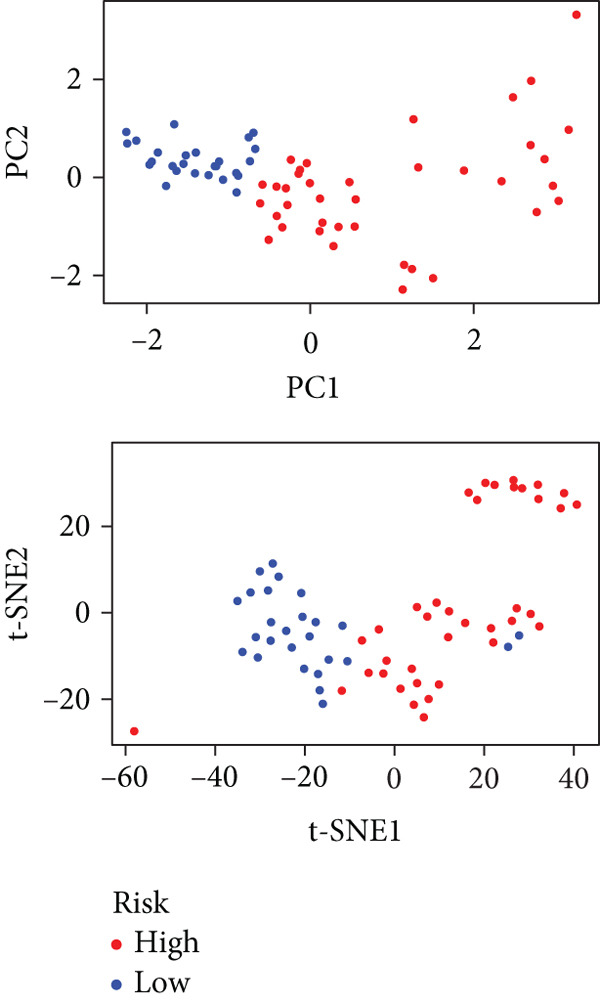
(i)

Sankey diagram showed that patients from HRGcluster A mainly corresponded to HRDEGcluster A and the high‐risk subgroup, while patients from HRGcluster B mainly corresponded to HRDEGcluster B and the low‐risk subgroup (Figure 9a). Additionally, contrasting HRGcluster A subtype, the PC of the HRGcluster B subtype showed a notably lower risk score (Figure 9b). Similarly, contrasting HRDEGcluster A subtype, the PC of the HRDEGcluster B subtype showed a notably lower risk score (Figure 9c). Differential expression analysis showed that most HRGs had notably elevated expression within a high‐risk subgroup, while WNT4, WNT1, WNT10B, WNT2B, WNT9B, PRKACA, PTCH1, BMP5, and BMP6 had notably elevated expression within a low‐risk subgroup (Figure 9d). The ssGSEA revealed a noteworthy disparity in the enrichment score of the HH signaling between PC patients with various risks. Specifically, the HH pathway exhibited a significantly lower ssGSEA score within the low‐risk subgroup (Figure 9e). Subsequently, the association between the model genes and HRGs was further evaluated. We found that DCBLD2 and ANLN were positively correlated with most HRGs. But WNT4, WNT10B, WNT2B, WNT9B, PTCH1, BMP5, and BMP6 were HRGs significantly negatively correlated with both DCBLD2 and ANLN (Figure 9f). In addition, the correlation between ANLN, DCBLD2, LY6D, and SERPINB3 and HH signaling was further investigated (Figures 9g, 9h, 9i, and 9j), and the results showed that DCBLD2 had the most significant positive correlation with HH signaling.

Figure 9Relationship between hedgehog (HH) signaling–related molecular subtypes and the prognostic model. (a) Sankey diagram. (b) The difference in HH signaling–related risk scores between HRGcluster A and HRGcluster B. (c) The difference in HH signaling–related risk scores between HRDEGcluster A and HRDEGcluster B. (d) The expressed difference of HH signaling–related genes in different risk subgroups. (e) The ssGSEA analysis indicated that the high‐risk subgroup had higher HH signaling activity. (f) The correlation between the model gene and HH signaling–related genes. ANLN (g), DCBLD2 (h), and LY6D (i) had significant positive correlation with HH signaling, while SERPINB3 (j) had no significant correlation with HH signaling (^ns^ *p* value > 0.05,  ^∗^ *p* value < 0.05,  ^∗∗^ *p* value < 0.01, and  ^∗∗∗^ *p* value < 0.001).

**Figure:**
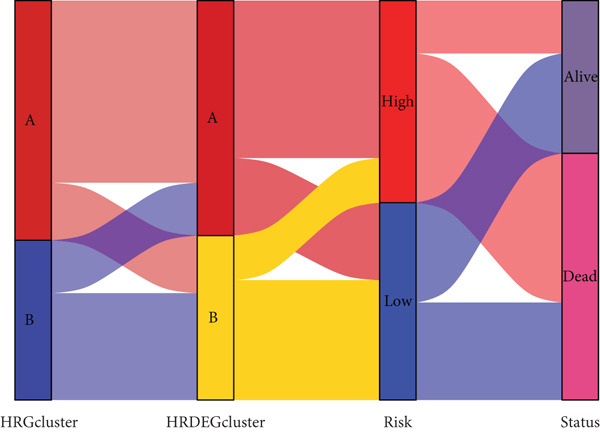
(a)

**Figure:**
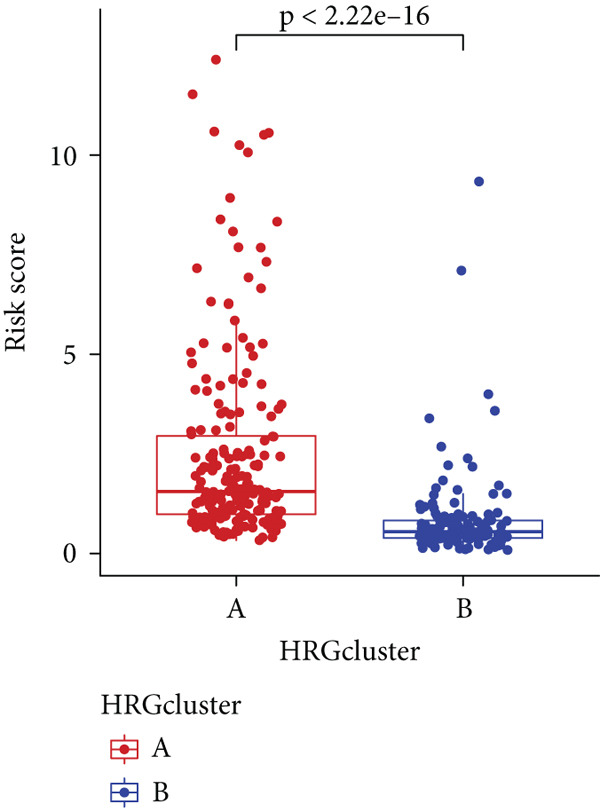
(b)

**Figure:**
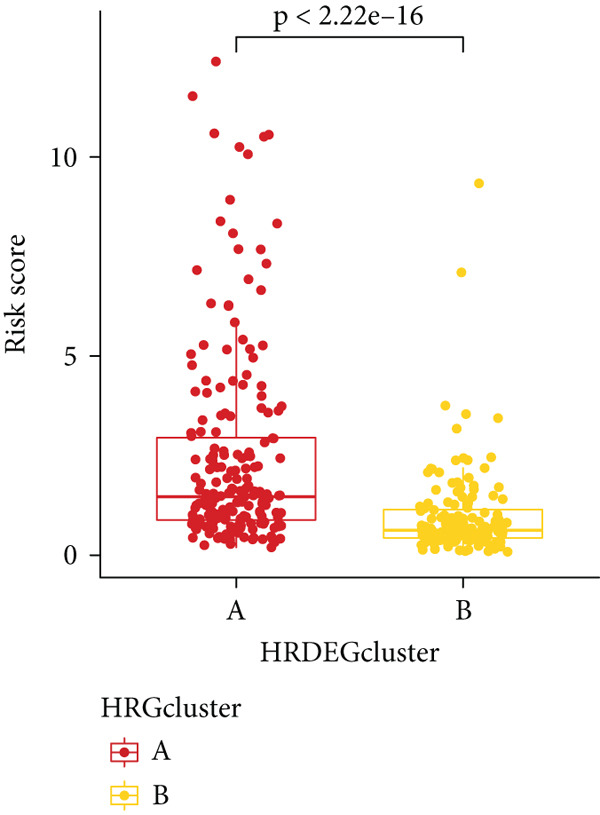
(c)

**Figure:**
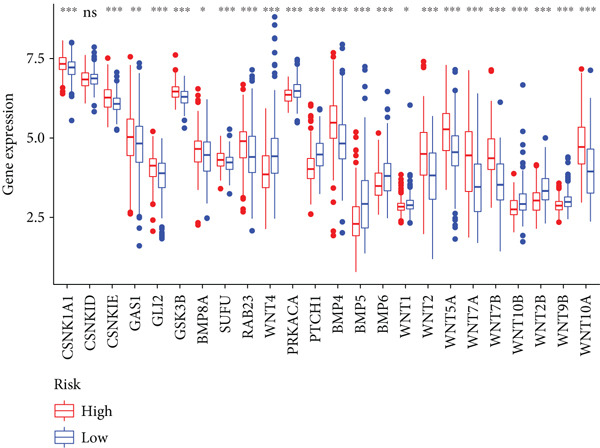
(d)

**Figure:**
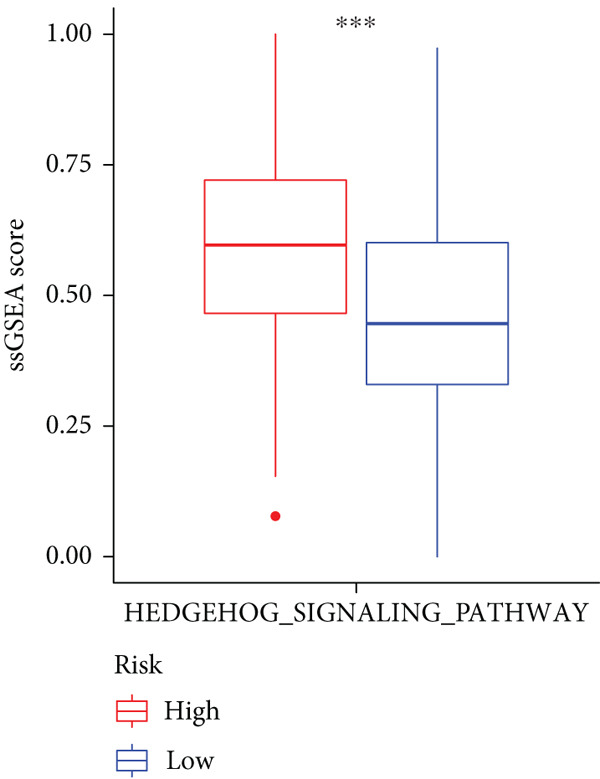
(e)

**Figure:**
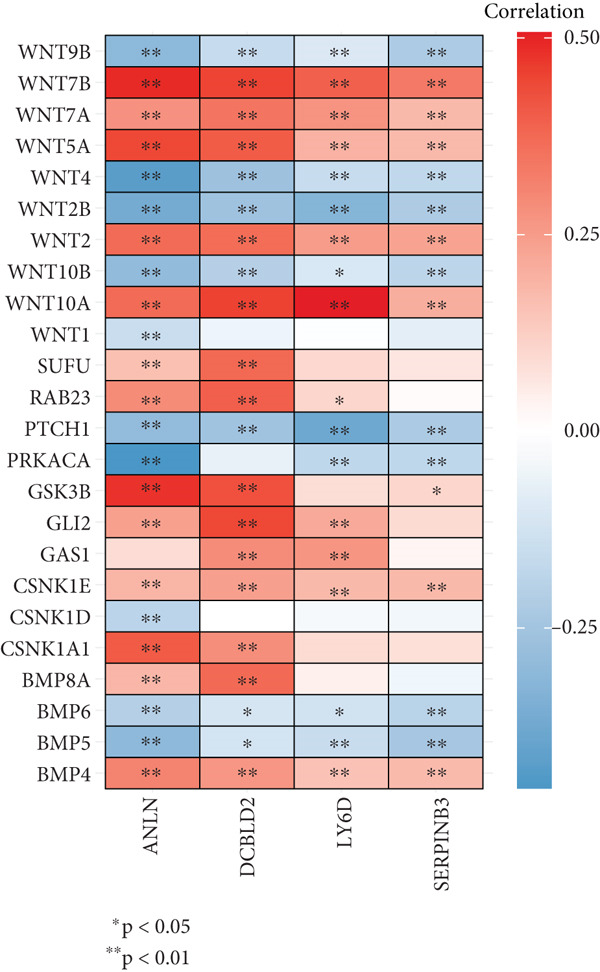
(f)

**Figure:**
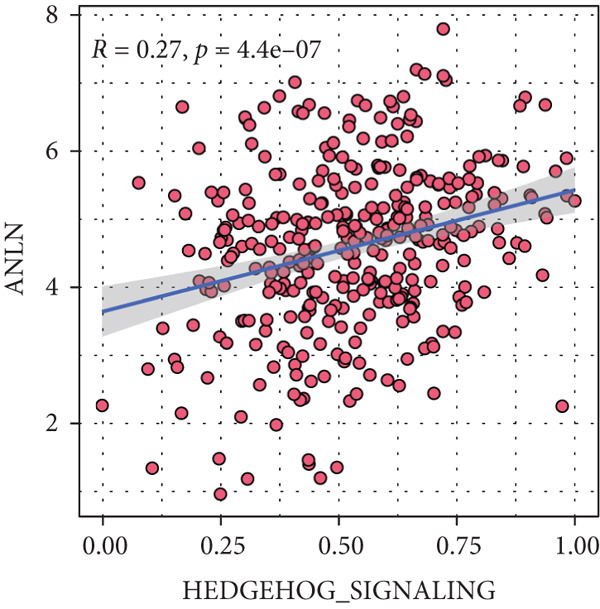
(g)

**Figure:**
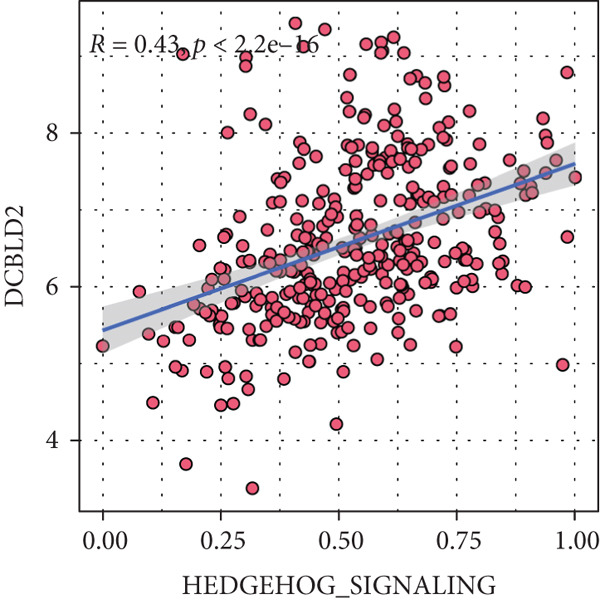
(h)

**Figure:**
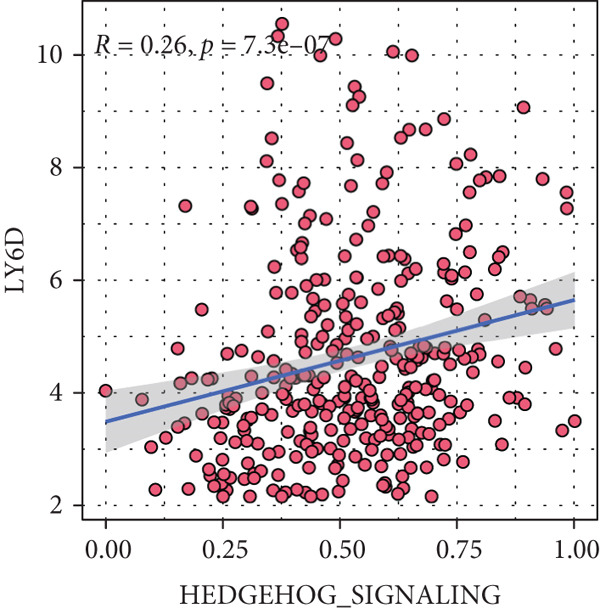
(i)

**Figure:**
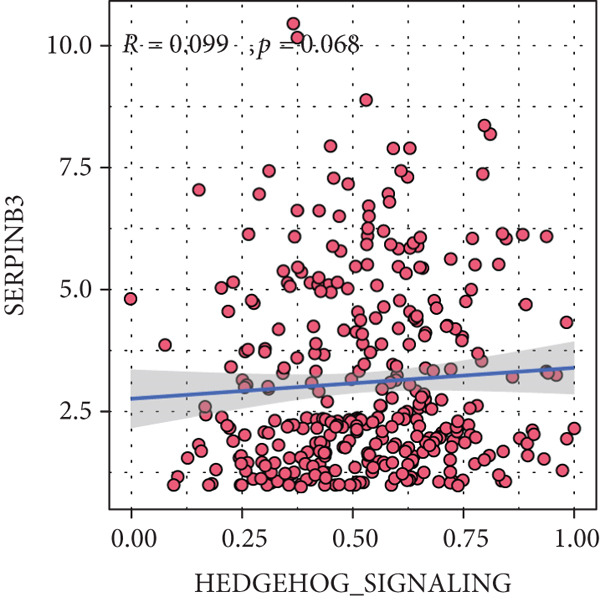
(j)

### 3.7. Immune Landscape and Immunotherapy Response Between Different Risk Subgroups

The immune microenvironment of different risk subgroups was investigated in this study. The ssGSEA analysis showed that low‐risk PC had higher infiltration of activated CD8+ T cells, effector memory CD8+ T cells, effector memory CD4+ T cells, activated B cells, monocytes, mast cells, eosinophils, and neutrophils, but high‐risk PC had higher infiltration of central memory CD8+ T cells, activated CD4+ T cells, gamma delta T cells, Type 2 T helper cells, NK cells, and neutrophils (Figure 10a). CIBERSORT results also suggested that low‐risk PC patients had higher infiltration of CD8+ T cells, naive B cells, and monocytes, but high‐risk PC had higher infiltration of follicular helper T cells, Tregs, resting NK cells, M0 macrophages, activated dendritic cells, and neutrophils (Figure 10b). The correlation of model genes, risk score, and HH signaling to immune cells was assessed. Results indicated that CD8+ T cells were notably negatively correlated with model genes, risk score, and HH signaling. M0 macrophages and activated dendritic cells were notably positively correlated with model genes, risk score, and HH signaling (Figure 10c). Besides, immunotherapy responses were further predicted. Low‐risk PC had higher dysfunction and MSI scores (Figure 10d,f), but lower exclusion and TIDE scores (Figure 10e,g). These results indicated that PC with a low risk was more sensitive to immunotherapy than PC with a high risk.

Figure 10Immune microenvironment and immunotherapy response in different risk subgroups. Immune cell subpopulation infiltration in high‐ and low‐risk subgroups was assessed by the (a) ssGSEA and (b) CIBERSORT algorithms. (c) The correlation of immune cell subpopulation and model genes, risk score, and hedgehog signaling ssGSEA score. (d) T cell dysfunction, (e) T cell exclusion, (f) MSI, and (g) TIDE scores in high‐ and low‐risk subgroups (^ns^ *p* value > 0.05,  ^∗^ *p* value < 0.05,  ^∗∗^ *p* value < 0.01, and  ^∗∗∗^ *p* value < 0.001).

**Figure:**
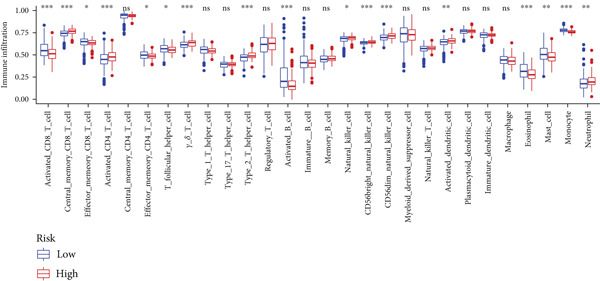
(a)

**Figure:**
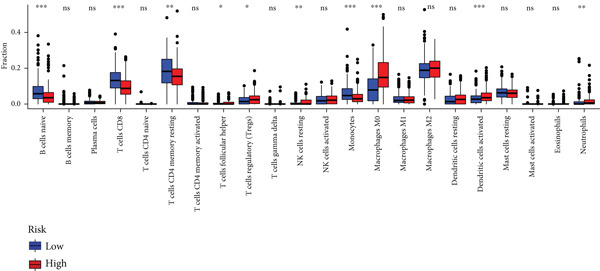
(b)

**Figure:**
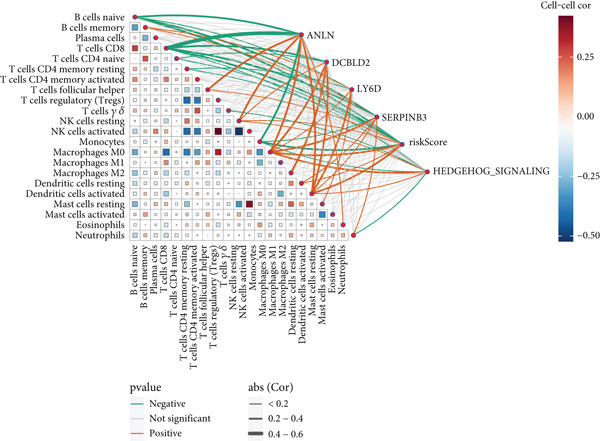
(c)

**Figure:**
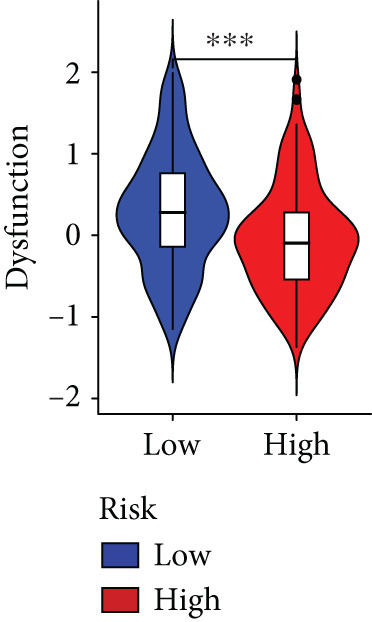
(d)

**Figure:**
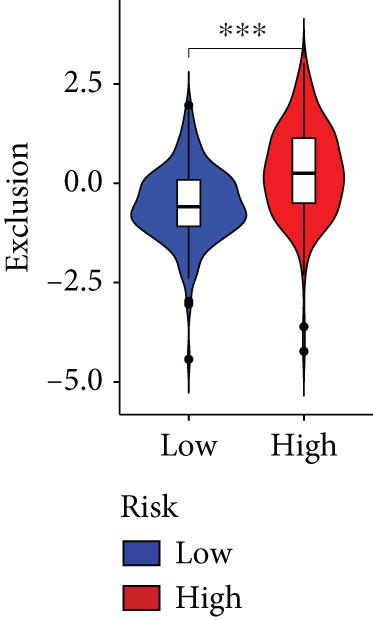
(e)

**Figure:**
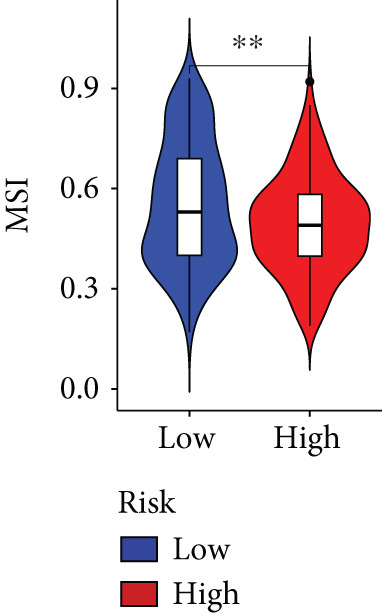
(f)

**Figure:**
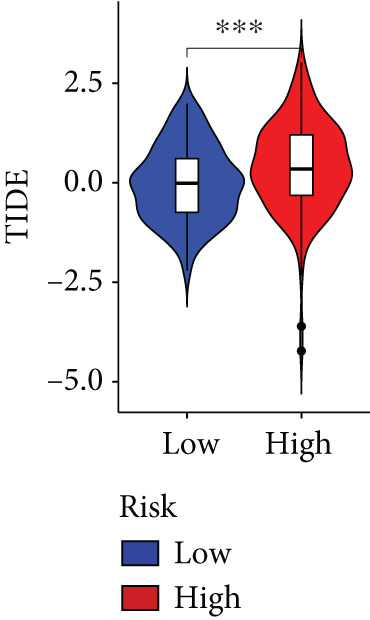
(g)

### 3.8. SERPINB3, LY6D, DCBLD2, and ANLN Are Upregulated in PC and Associated With Poor Prognosis

By the GEPIA platform, we conducted an expression analysis at the RNA level and discovered significant upregulation of SERPINB3, LY6D, DCBLD2, and ANLN within PC tumor tissues (Figures 11a, 11b, 11c, and 11d). Furthermore, we analyzed the protein expression levels of ANLN, DCBLD2, LY6D, and SERPINB3 using the UALCAN platform, which were notably upregulated within PC tumor tissues (Figures 11e, 11f, 11g, and 11h). Additionally, the elevated expression of SERPINB3, LY6D, DCBLD2, and ANLN was linked to an unfavorable prognosis in patients with PC (Figures 11i, 11j, 11k, and 11l). Immunohistochemistry images from the HPA database also manifested that the protein expression levels of ANLN, LY6D, and SERPINB3 are notably higher in PC tumor tissues (Figures 12a, 12b, and 12c). Immunofluorescence images from the HPA database also showed that ANLN protein is mainly located in the nucleoplasm and midbody, DCBLD2 protein is mainly located in the plasma membrane, cytosol, and Golgi apparatus, and SERPINB3 protein is mainly located in the cytosol and plasma membrane (Figures 12d, 12e, and 12f).

Figure 11ANLN, DCBLD2, LY6D, and SERPINB3 are upregulated in pancreatic cancer (PC) and associated with poor prognosis. ANLN (a), DCBLD2 (b), LY6D (c), and SERPINB3 (d) expressions in PC tissues were notably higher than that in normal tissues at the RNA level. ANLN (e), DCBLD2 (f), LY6D (g), and SERPINB3 (h) expressions in PC tissues were notably higher than that in normal tissues at the protein level. The high expressions of ANLN (i), DCBLD2 (j), LY6D (k), and SERPINB3 (l) were significantly associated with a poor prognosis for PC (^ns^ *p* value > 0.05,  ^∗^ *p* value < 0.05,  ^∗∗^ *p* value < 0.01, and  ^∗∗∗^ *p* value < 0.001).

**Figure:**
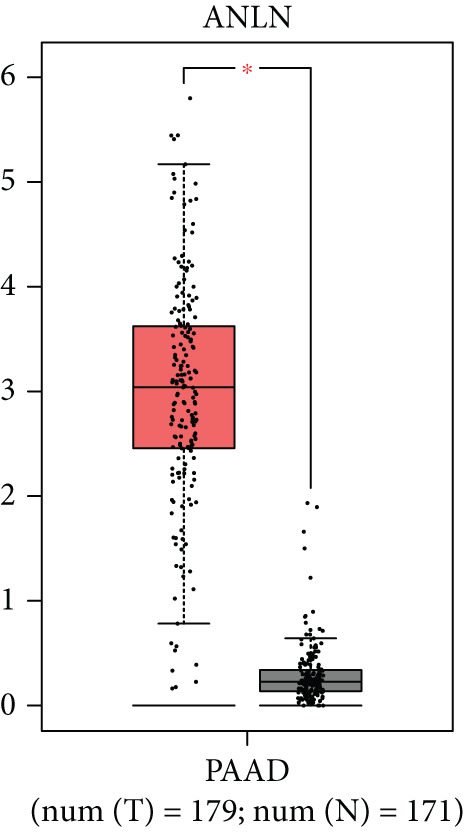
(a)

**Figure:**
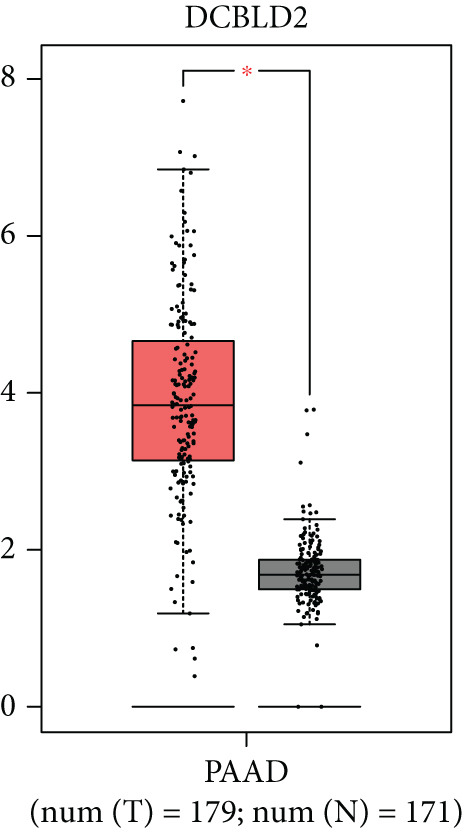
(b)

**Figure:**
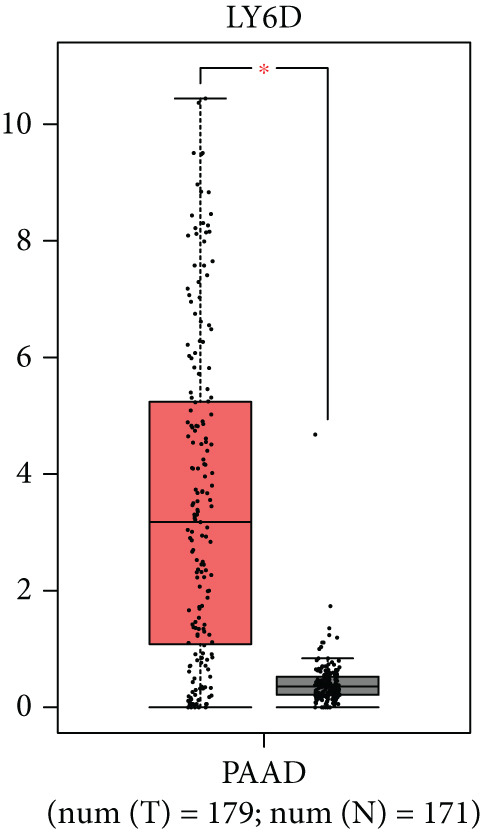
(c)

**Figure:**
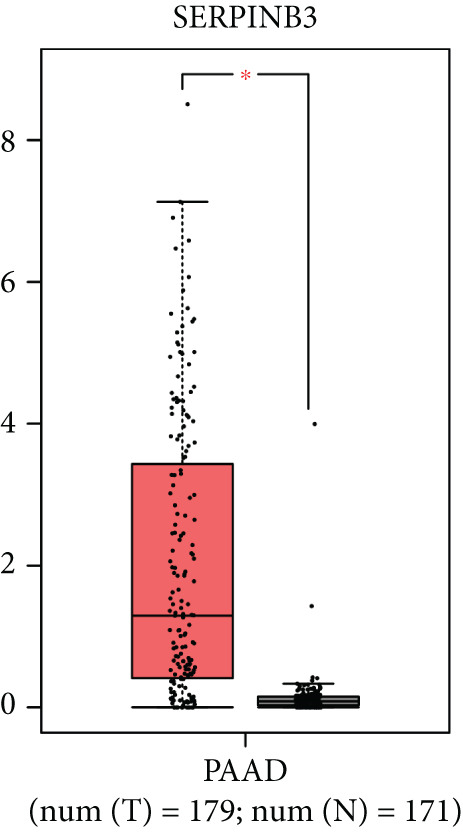
(d)

**Figure:**
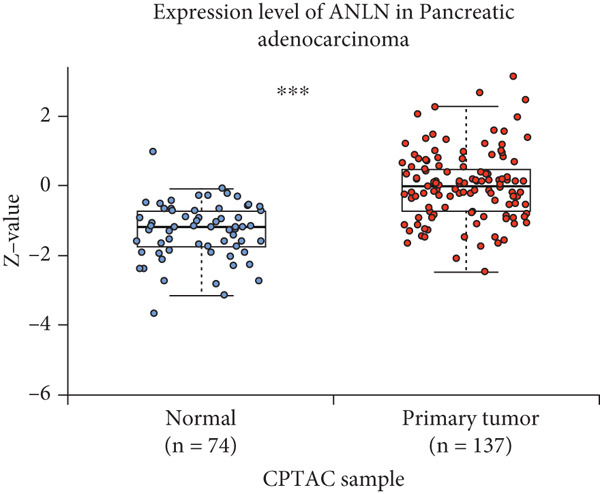
(e)

**Figure:**
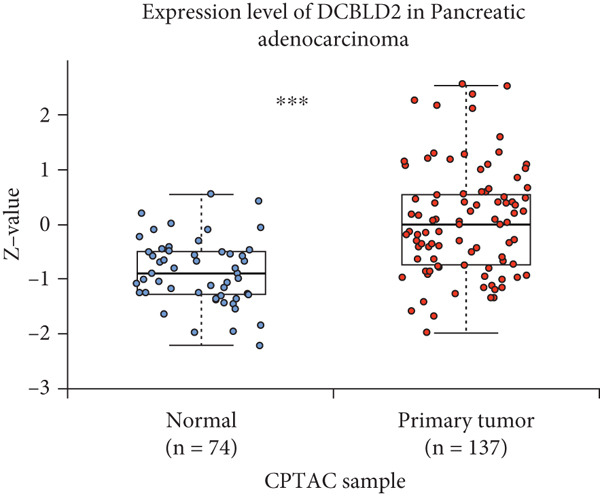
(f)

**Figure:**
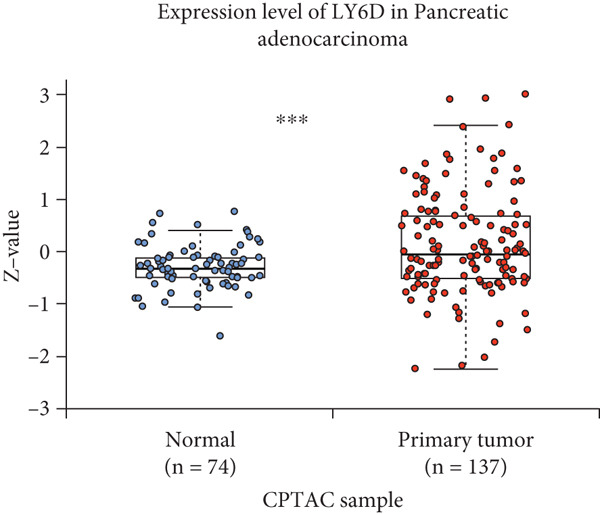
(g)

**Figure:**
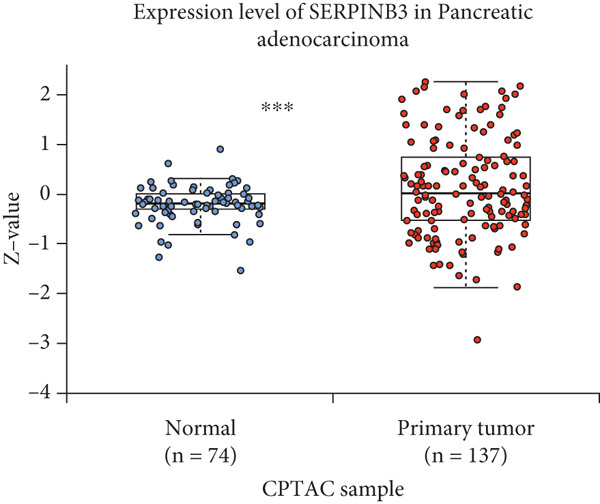
(h)

**Figure:**
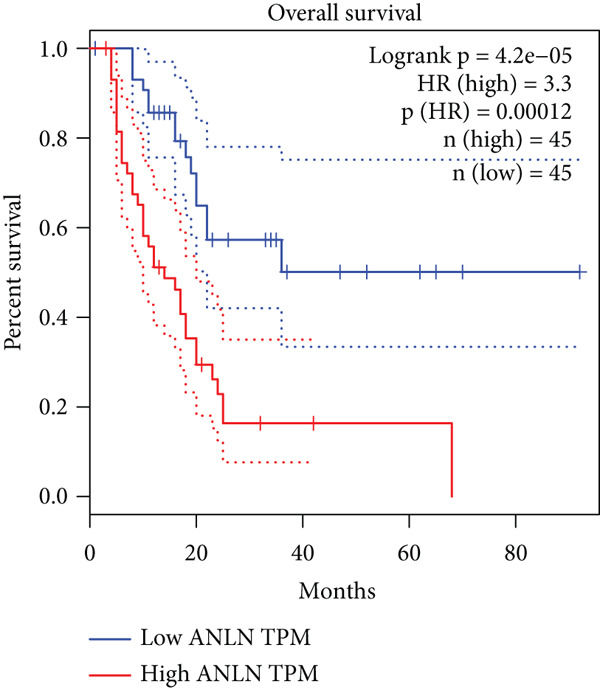
(i)

**Figure:**
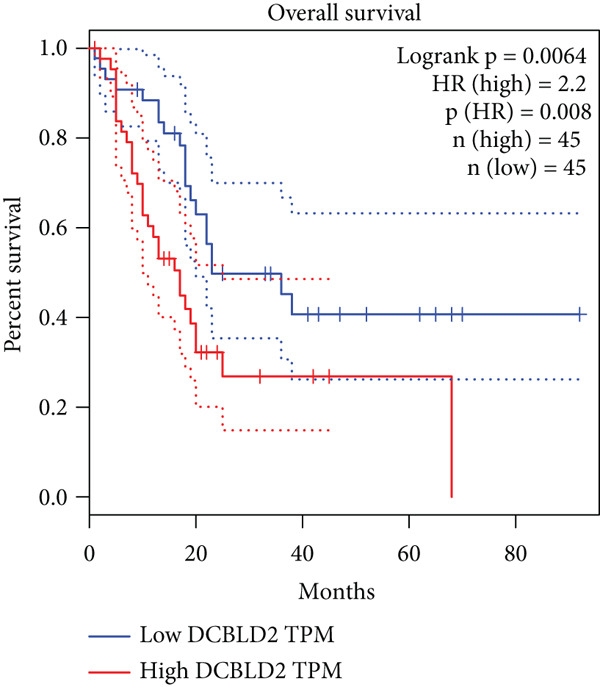
(j)

**Figure:**
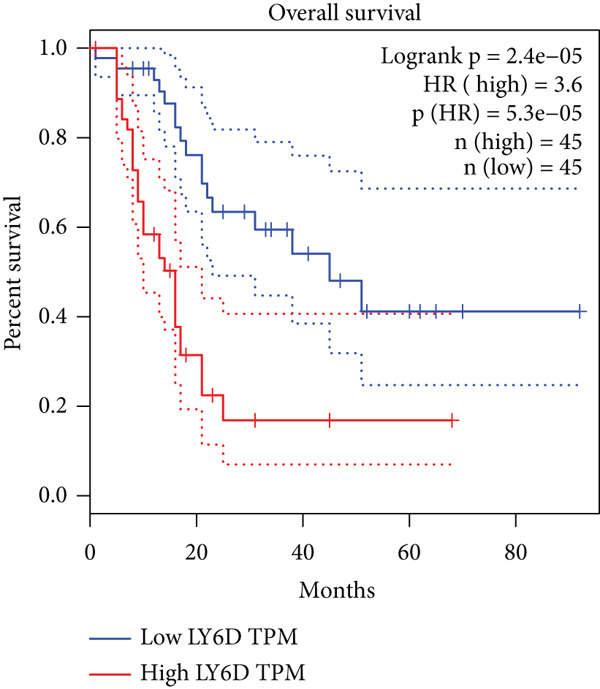
(k)

**Figure:**
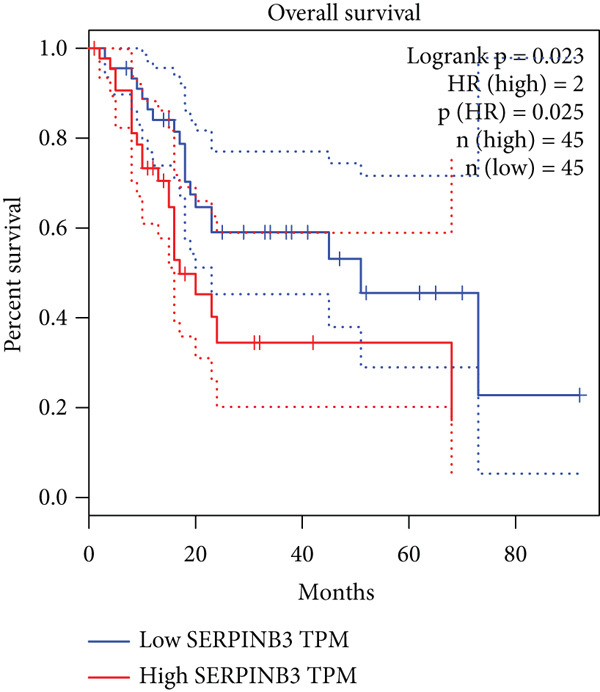
(l)

Figure 12Immunohistochemistry and immunofluorescence images of ANLN, DCBLD2, LY6D, and SERPINB3. Immunohistochemistry images of ANLN (a), LY6D (b), and SERPINB3 (c) in pancreatic cancer and normal tissues were obtained from the Human Protein Atlas (HPA) database (scale bar: 200 *μ*m). Immunofluorescence images of ANLN (d), DCBLD2 (e), and SERPINB3 (f) were obtained from the HPA database (scale bar: 20 *μ*m).

**Figure:**
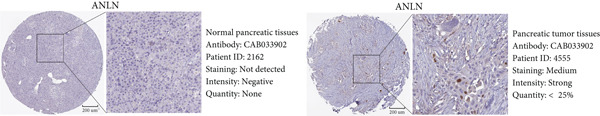
(a)

**Figure:**
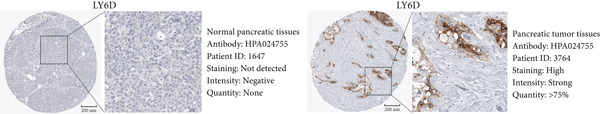
(b)

**Figure:**
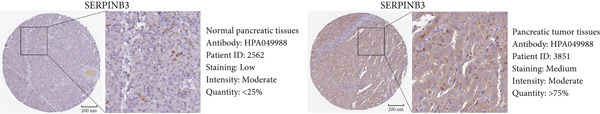
(c)

**Figure:**
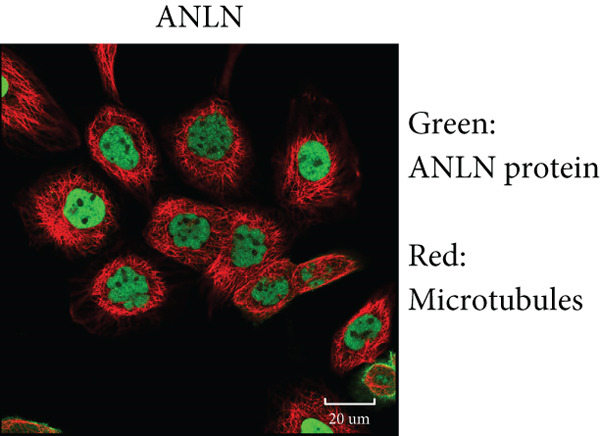
(d)

**Figure:**
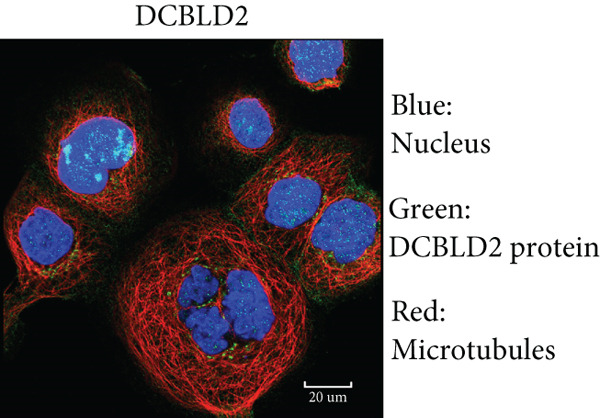
(e)

**Figure:**
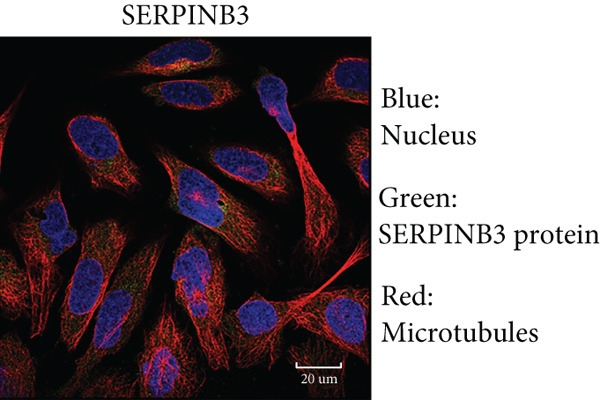
(f)

### 3.9. Single‐Cell Transcriptomics Reveals a Significant Positive Correlation Between DCBLD2 and HH Signaling

To further explore HH signaling characteristics and the relationship between HH signaling and DCBLD2 at the single‐cell level, we obtained scRNA‐seq from three primary PCs. After filtering with preset quality control criteria, 26,308 cells were collected (Supporting Information 1: Figure S1). By dimensionality reduction, these 26,308 cells were grouped into 22 cell clusters (Figure 13a). Cell subtypes were first annotated with reference to Human Primary Cell Atlas Data (Supporting Information 1: Figure S2). Then, we analyzed the expression levels of typical marker genes in each cell cluster (Supporting Information 1: Figure S3). Combining these two approaches, the cell clusters are finally annotated into eight cell subsets: T cells (38.22%), epithelial cells (30.55%), fibroblasts (15.66%), monocytes (2.71%), macrophages (7.71%), NK cells (2.62%), B cells (1.81%), and endothelial cells (0.77%) (Figure 13b and Supporting Information 1: Figure S4). The expression of DCBLD2 in each cell subset was analyzed. Results showed that DCBLD2 was relatively highly expressed in epithelial cells and fibroblasts (Figure 13c,d). HH signaling activity in each cell subset was assessed, and the results indicated that HH signaling has relatively higher activity in fibroblasts, epithelial cells, and endothelial cells (Figure 13e). The colocalization analysis showed that the distribution of DCBLD2 and HH signaling was very consistent (Figure 13f). All the cells and each cell subpopulation were divided into the DCBLD2‐high group or DCBLD2‐low group according to the median value of DCBLD2 expression. In all the cells, the Wilcoxon rank sum test showed that the DCBLD2‐high group had a significantly higher HH signaling score (Figure 13g). Subsequently, the relationship between DCBLD2 and HH signaling was explored in each cell subset. Results showed that in the epithelial cells, fibroblasts, macrophages, and endothelial cells, the DCBLD2‐high group had a significantly higher HH signaling score (Figure 13h). To further investigate the characteristics of HH signaling in malignant cells, epithelial cells were extracted. After dimensionality reduction clustering, 19 cell clusters were identified (Figure 13i). CNV for each cell cluster was calculated using inferCNV, and T cells and B cells were used as references (Figures 13j, 13k, and 13l). By comparing CNV with T cells and B cells, normal epithelial cells and malignant cells were identified (Figure 13m). Further analysis showed that the activity of HH signaling in malignant cells was significantly higher than that in normal epithelial cells (Figure 13n,o). In addition, in both normal epithelial cells and malignant cells, the DCBLD2‐high group had a significantly higher HH signaling score (Figure 13p).

Figure 13Single‐cell analysis. (a) UMAP plot showed that 22 cell clusters were identified. (b) UMAP plot showed that eight cell subsets were annotated. (c) UMAP plot visualized the expression of DCBLD2. (d) DCBLD2 had relatively higher expression levels in epithelial cells and fibroblasts. (e) The activity of hedgehog (HH) signaling in each cell subset was calculated by UCell, singscore, and AddModuleScore algorithms. (f) Colocalization analysis of HH signaling and DCBLD2. (g) The DCBLD2 high‐expression group had a significantly higher HH signaling score. (h) In the epithelial cells, fibroblasts, macrophages, and endothelial cells, the DCBLD2 high‐expression group had a significantly higher HH signaling score. (i) UMAP plot showed that epithelial cells were further extracted and reduced in dimension to 19 cell clusters. (j) Copy number variation (CNV) in 19 cell clusters, T cells, and B cells was calculated by inferCNV. (k) CNV score of T cells and B cells. (l) CNV score of 19 cell clusters. (m) UMAP plot showed that normal epithelial cells and malignant cells were identified by comparing the CNV score with T cells and B cells. (n) The activity of HH signaling in epithelial cells and malignant cells was calculated by UCell, singscore, and AddModuleScore algorithms. (o) The activity of HH signaling in malignant cells was significantly higher than that in normal epithelial cells. (p) In both normal epithelial cells and malignant cells, the DCBLD2 high‐expression group had a significantly higher HH signaling score.

**Figure:**
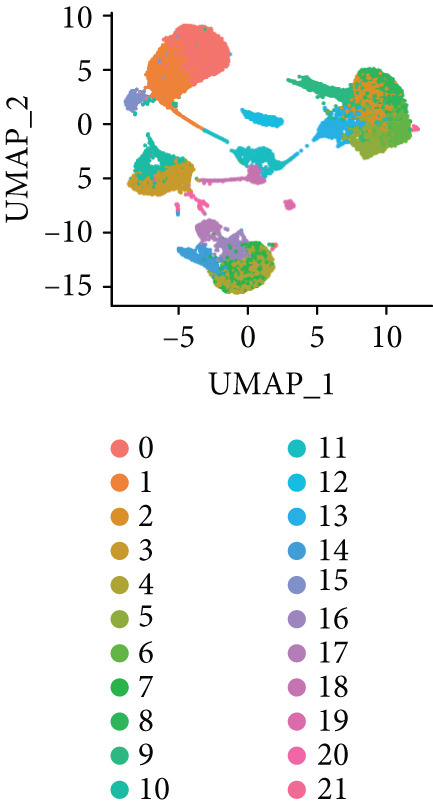
(a)

**Figure:**
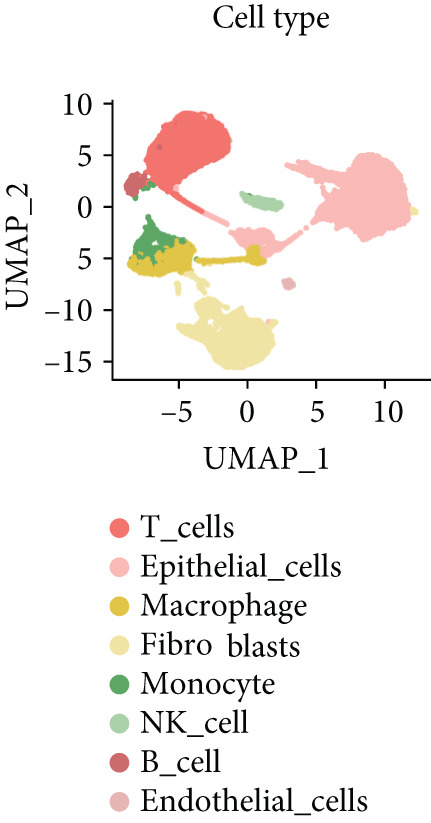
(b)

**Figure:**
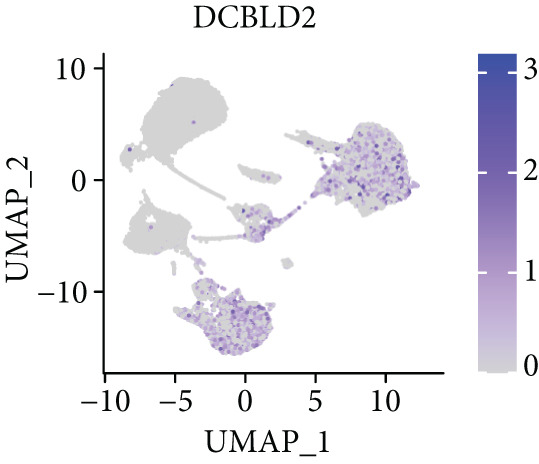
(c)

**Figure:**
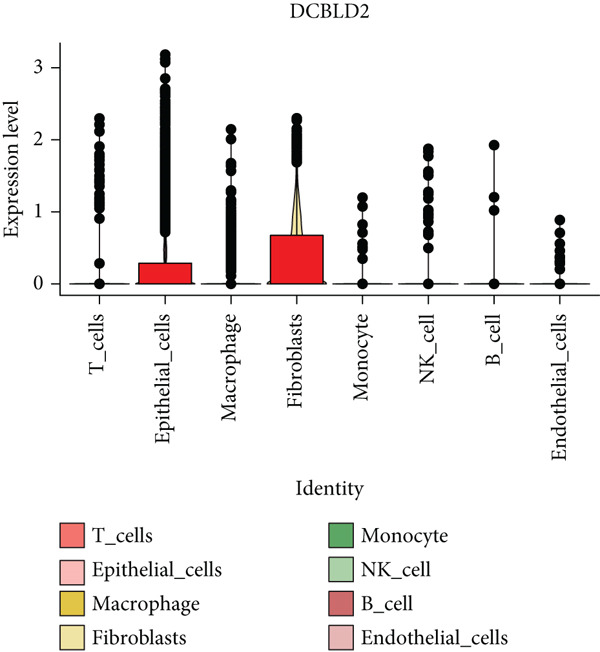
(d)

**Figure:**
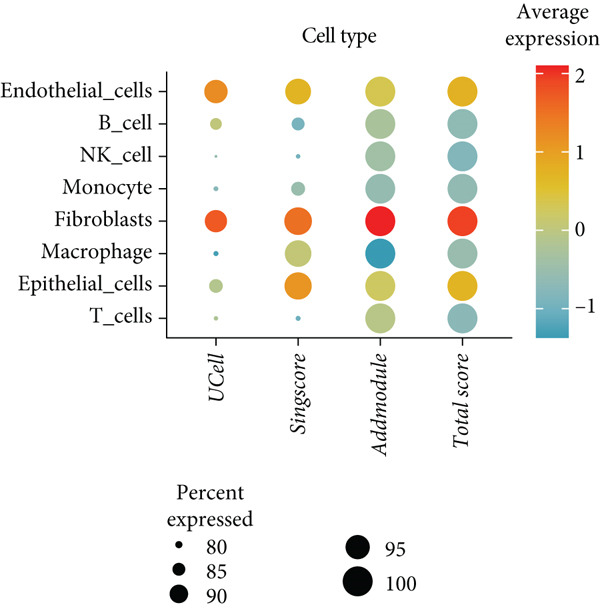
(e)

**Figure:**
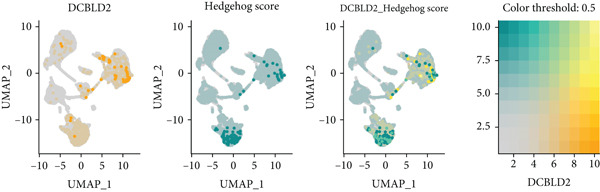
(f)

**Figure:**
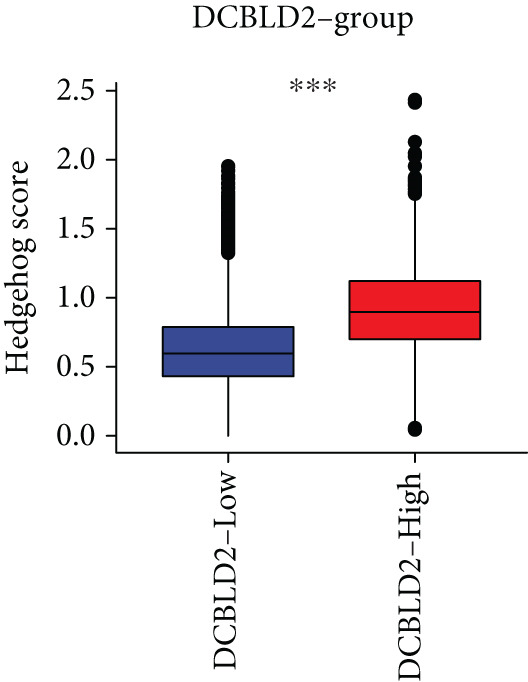
(g)

**Figure:**
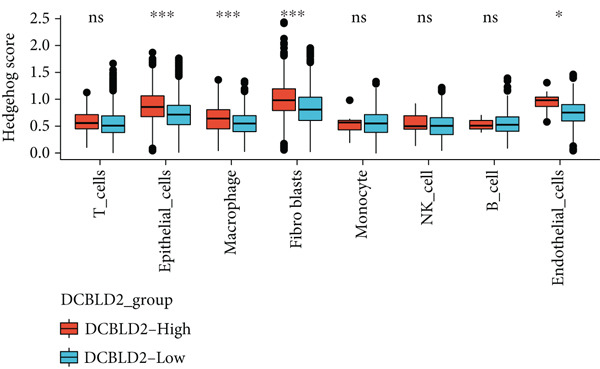
(h)

**Figure:**
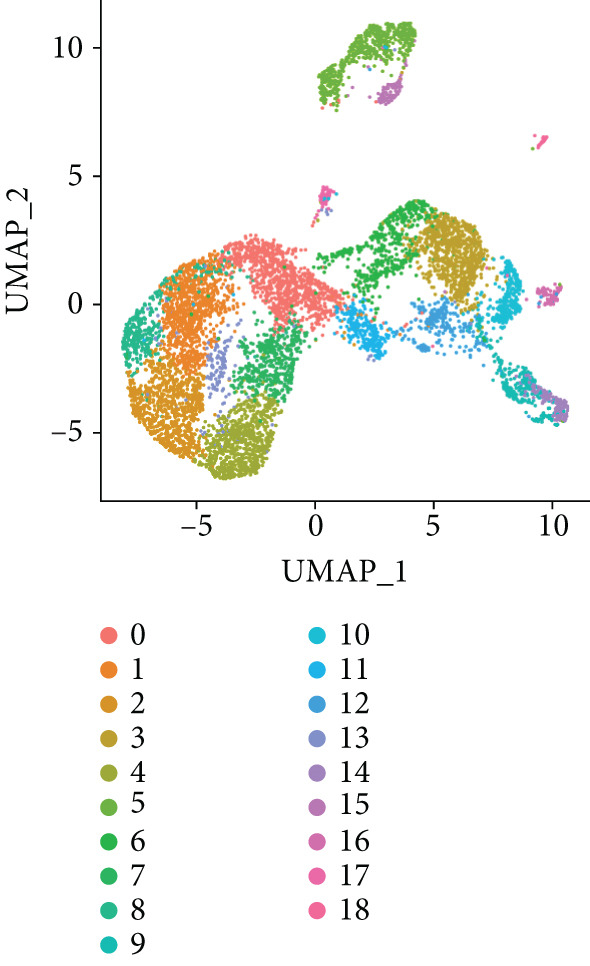
(i)

**Figure:**
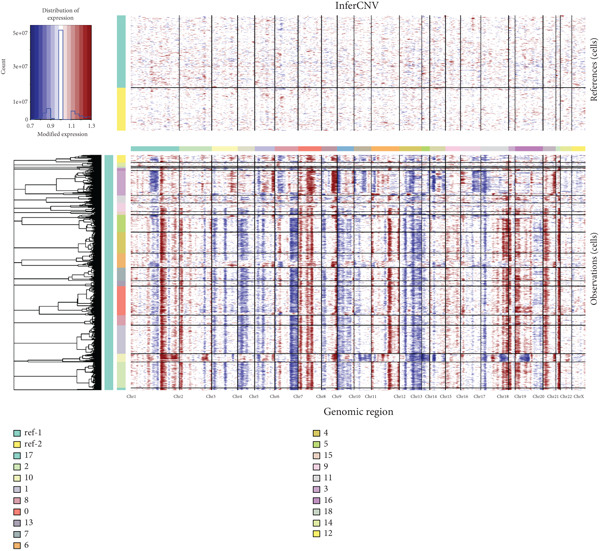
(j)

**Figure:**
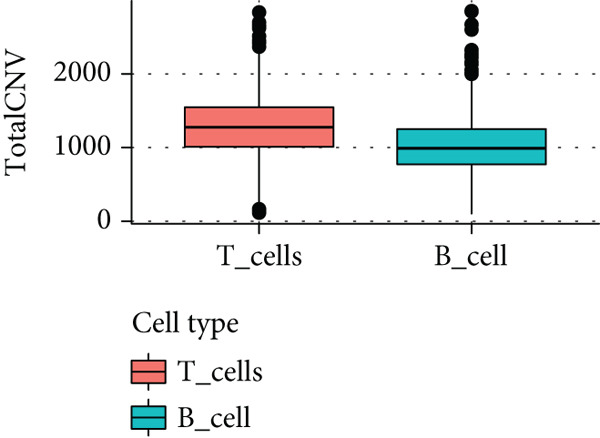
(k)

**Figure:**
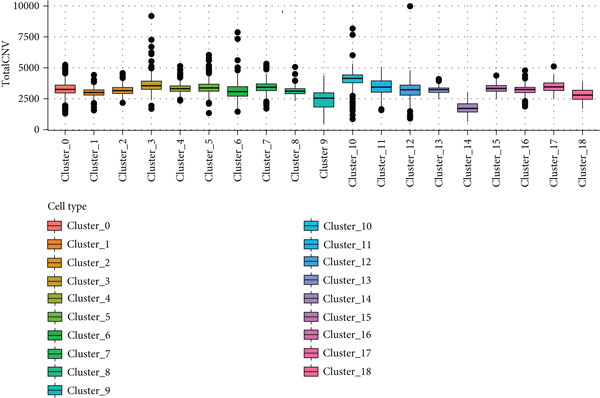
(l)

**Figure:**
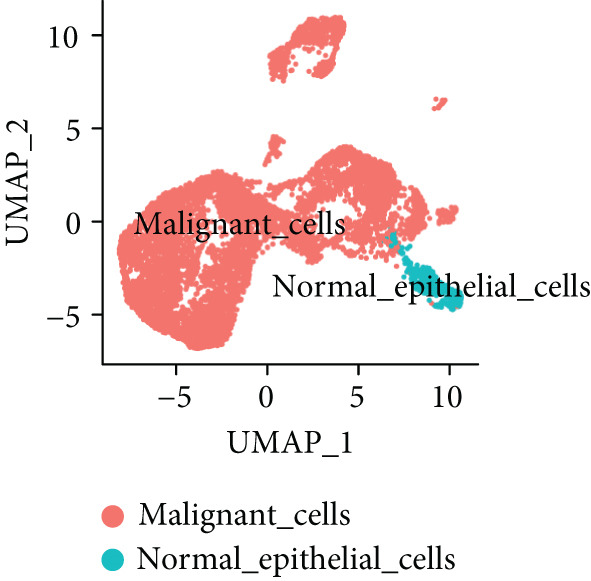
(m)

**Figure:**
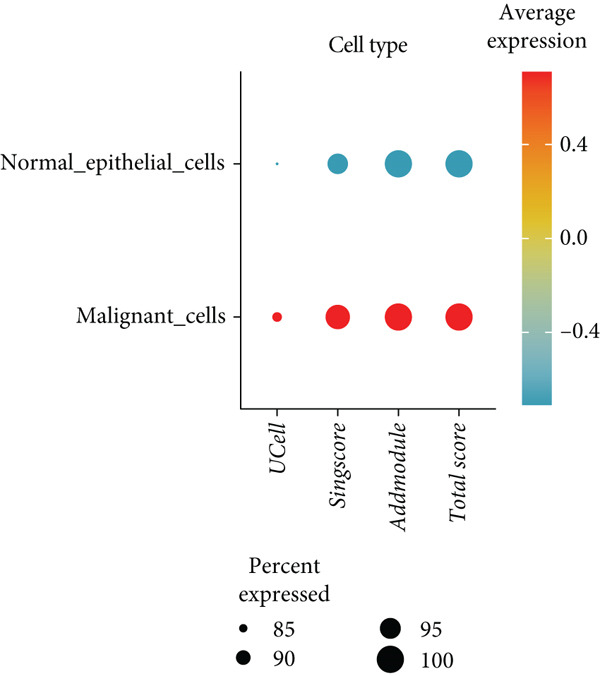
(n)

**Figure:**
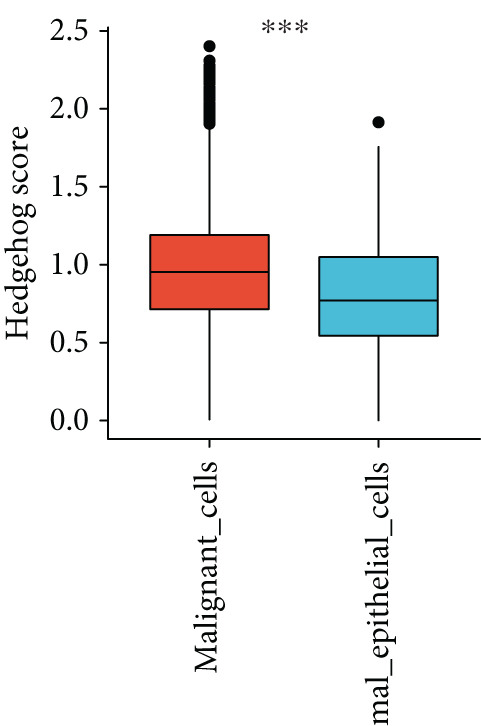
(o)

**Figure:**
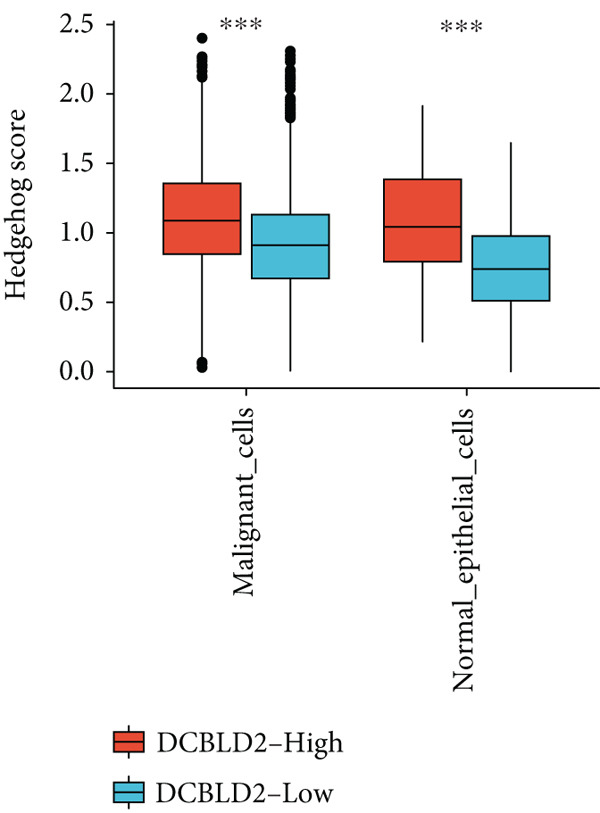
(p)

### 3.10. DCBLD2 Knockdown Downregulates HH Signaling and Inhibits the Proliferation, Migration, and Invasion of PC Cells

Based on the previous results, we find that of the four model genes, DCBLD2 has the most significant correlation with HH signaling. To further explore the function and mechanism of DCBLD2 in PC cells, we transfected siRNA targeting DCBLD2 into PANC‐1 and BxPC‐3 cells. PCR results indicated that the expression of DCBLD2 was successfully knocked down (Figure 14a,d). In addition, after DCBLD2 was knocked down, the expression of HRGs GLI1 and PTCH1 was also significantly reduced (Figures 14b, 14c, 14e, and 14f). CCK‐8 experiments showed that DCBLD2 knockdown could suppress the proliferation of PANC‐1 and BxPC‐3 cells (Figure 14g,h). Scratch experiments showed that DCBLD2 knockdown could significantly suppress the migration of PANC‐1 and BxPC‐3 cells (Figure 14i,j). Transwell invasion experiments showed that DCBLD2 knockdown could suppress the invasion of PANC‐1 and BxPC‐3 cells (Figure 14k,l). These results suggested that DCBLD2 can affect the proliferation and metastasis of PC cells by regulating HH signaling.

Figure 14DCBLD2 knockdown downregulates hedgehog (HH) signaling and inhibits the proliferation, migration, and invasion of PC cells. (a) The qRT‐PCR manifested that the expression of DCBLD2 was successfully knocked down in PANC‐1 cells. The expression of (b) GLI1 and (c) PTCH1 was significantly reduced after DCBLD2 knockdown in PANC‐1 cells. (d) The qRT‐PCR manifested that the expression of DCBLD2 was successfully knocked down in BxPC‐3 cells. The expression of (e) GLI1 and (f) PTCH1 was significantly reduced after DCBLD2 knockdown in BxPC‐3 cells. Cell viability fold of (g) PANC‐1 cells and (h) BxPC‐3 cells on Day 1, Day 2, and Day 3 was assessed by the CCK‐8 assay. Scratch assays indicated that the migration ability of (i) PANC‐1 cells and (j) BxPC‐3 cells was significantly inhibited after DCBLD2 knockdown (the scale bar represents 200 *μ*m). Transwell invasion assays indicated that the invasion ability of (k) PANC‐1 cells and (l) BxPC‐3 cells was significantly inhibited after DCBLD2 knockdown (the scale bar represents 100 *μ*m) (^ns^ *p* value > 0.05,  ^∗^ *p* value < 0.05,  ^∗∗^ *p* value < 0.01, and  ^∗∗∗^ *p* value < 0.001).

**Figure:**
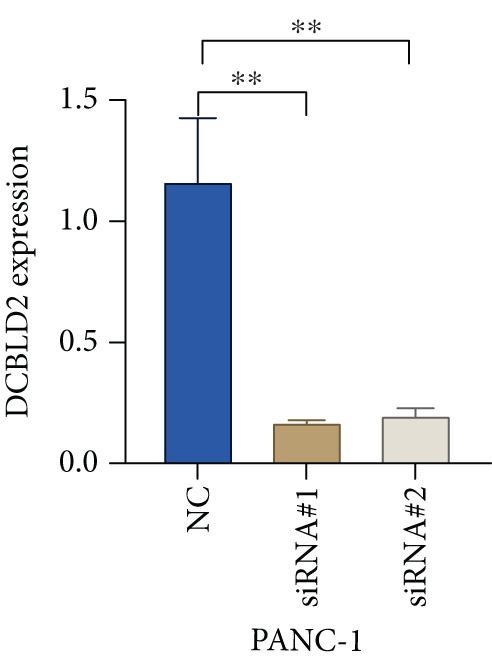
(a)

**Figure:**
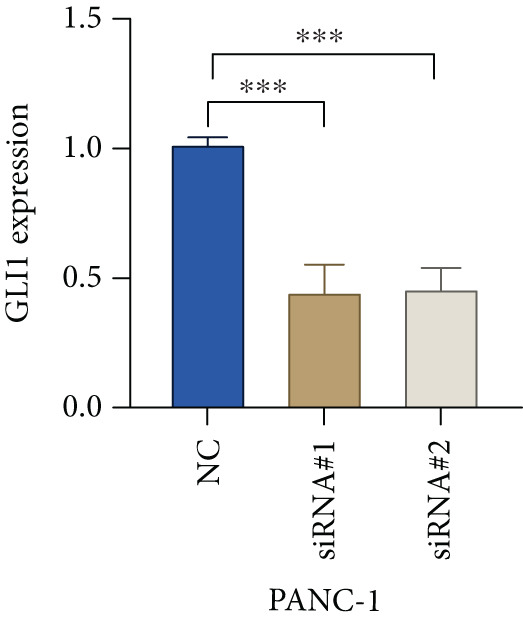
(b)

**Figure:**
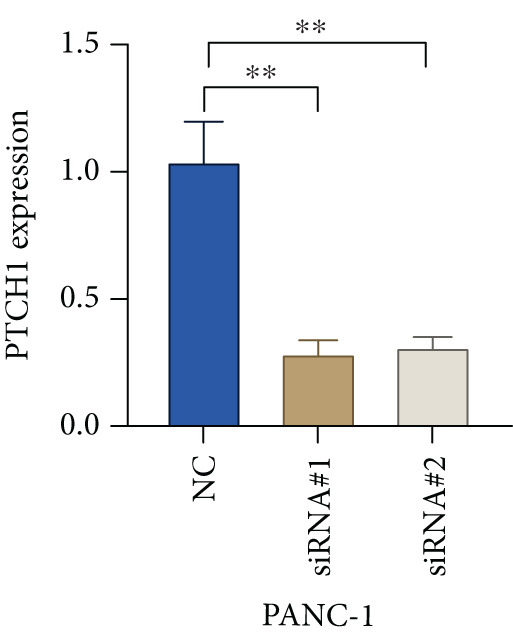
(c)

**Figure:**
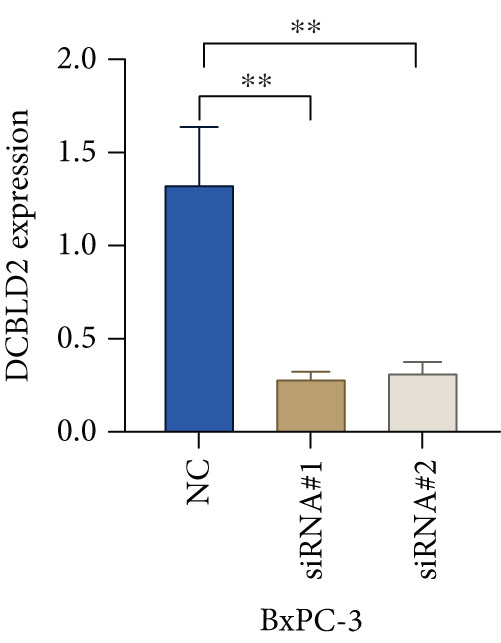
(d)

**Figure:**
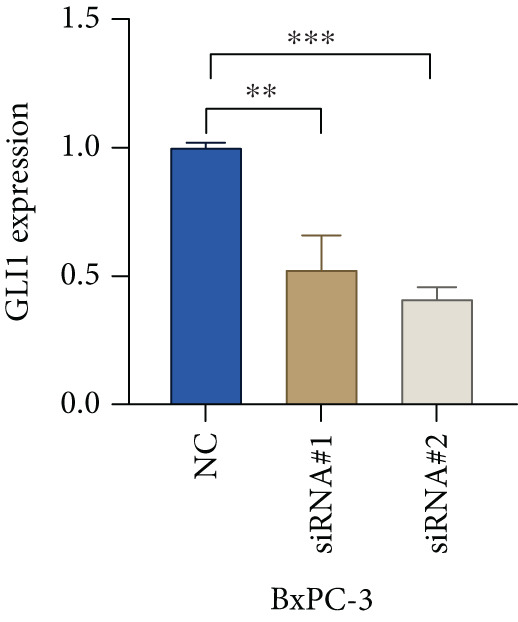
(e)

**Figure:**
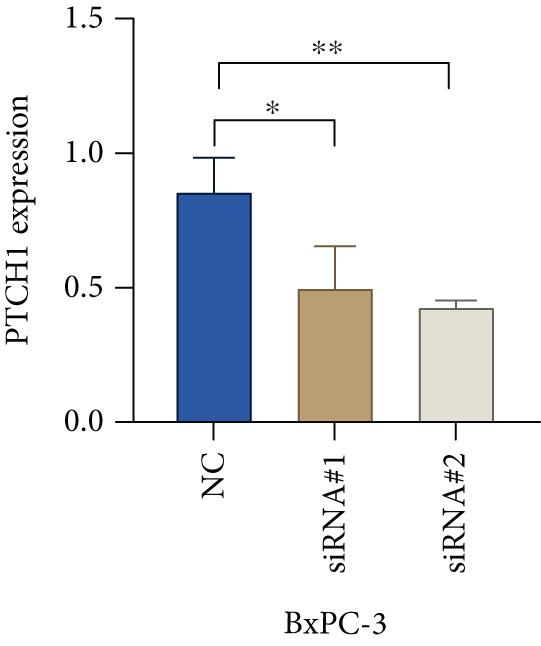
(f)

**Figure:**
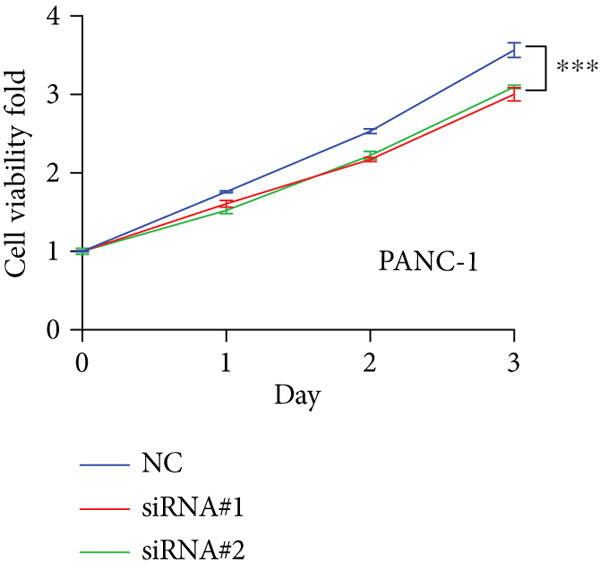
(g)

**Figure:**
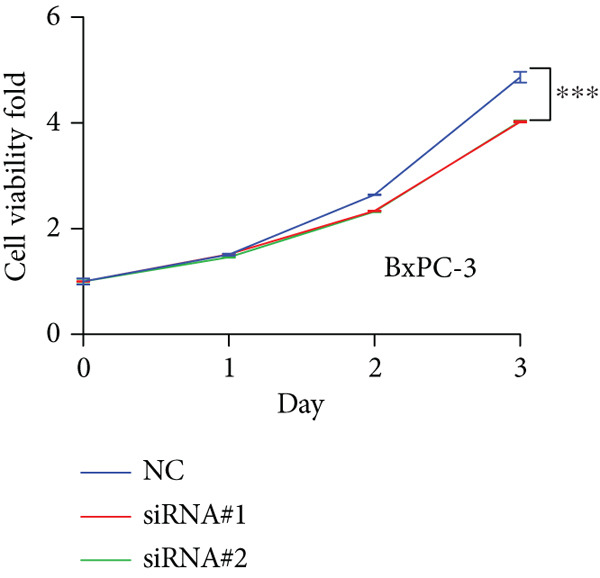
(h)

**Figure:**
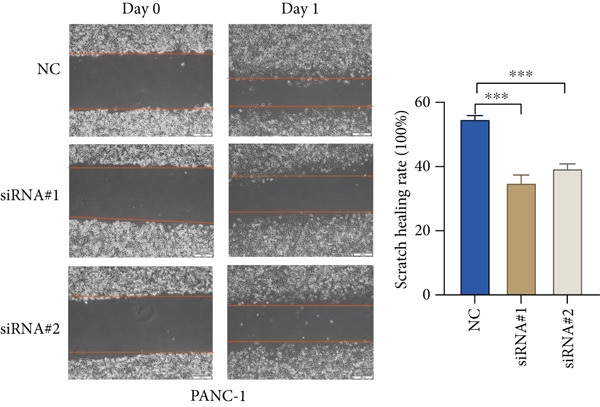
(i)

**Figure:**
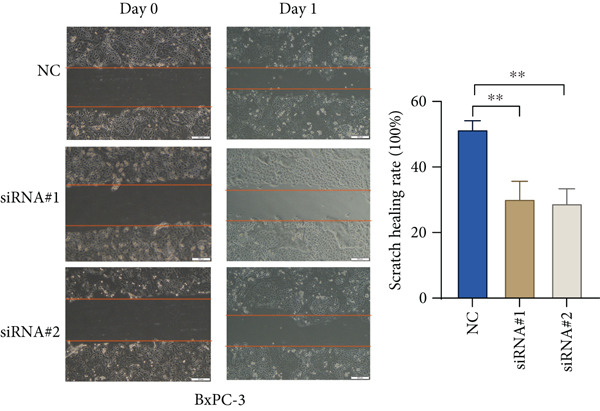
(j)

**Figure:**
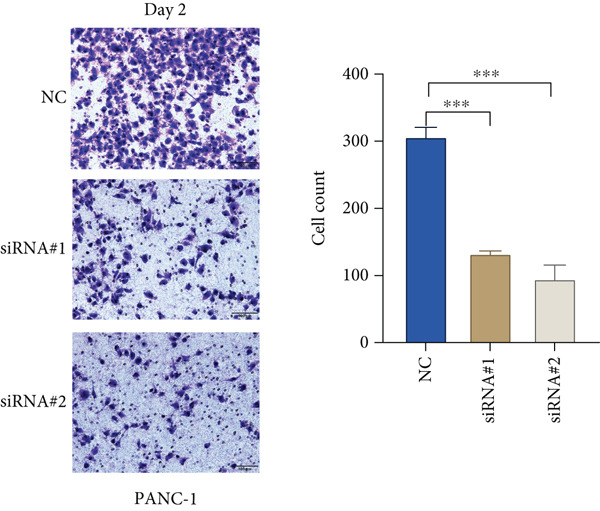
(k)

**Figure:**
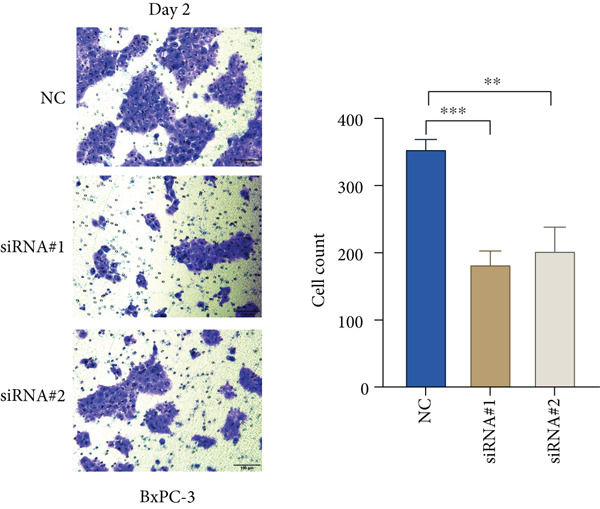
(l)

## 4. Discussion

HH signaling regulates cell fate, proliferation, and differentiation in numerous developmental processes across both vertebrates and invertebrates [46]. Recently, numerous researchers uncovered that the dysregulated activation of HH signaling is engaged in cancer occurrence [47]. HH signaling can induce the generation of heterogeneous subpopulations of cancer cells that participate in angiogenesis and immortality. These help in cancer relapse, metastasis, chemotherapy/radiotherapy resistance, and rapid adaptation to changes in TME, thereby avoiding apoptosis of tumor cells [48]. Berman et al. showed that colorectal cancer development may be caused by the inactivation of the HH pathway [49]. A study focusing on prostate cancer revealed that HH was the predominant signaling pathway affected by RCC2 knockdown in DU145 cells. Hence, regulating the HH pathway could potentially aid in managing metastatic and recurrent prostate cancer [15]. PC is a highly invasive tumor that is mostly diagnosed at an advanced stage [50]. The effects of chemotherapy and radical surgery are not optimistic, and the 5‐year survival rate is not beyond 10% [51]. Hence, deciphering the key components of HH in PC is vital for clinically targeted therapies. However, there is limited research on the specific mechanisms of HH signaling in PC. At present, multiomics and single‐cell analysis have been widely used in cancer research [52–54]. In this study, we utilized integrated bulk and single‐cell data and an in vitro experiment to comprehensively analyze the potential functions of HH signaling in PC.

GSEA revealed that the HH pathway is highly expressed in PC tissue, suggesting its potential involvement in the occurrence and development of PC. Using Cox regression analysis, we identified a total of 24 HRGs that are significantly associated with PC prognosis. Notably, a majority of these HRGs are known to play critical functions within the WNT pathway, which governs essential biological processes. Examples of such HRGs include WNT9B, WNT2B, and WNT10B. These highlight the potential role of dysregulated WNT signaling within PC progression and provide potential targets for further investigation and therapeutic interventions [55]. CSNK1A1 (a negative regulator of WNT) and some growth‐related proteins like BMP8A and BMP4 were also found to be associated with PC prognosis [56]. Subsequently, we determined HH signaling‐linked molecular subtypes of PC and divided all patients into two subtypes: HRGcluster A and HRGcluster B. HRGcluster A had a poorer prognosis and was significantly enriched in functional areas such as axon guidance and ECM receptor interaction. One report has it that disrupting the SLIT2‐ROBO pathway, an axon guidance factor, in PC may enhance metastasis and make PC cells easier for neural invasion [57]. A research investigation demonstrated that the dismal prognosis of PC can be largely attributed to its robust fibrotic stromal reaction, characterized by elevated levels of ECM collagen I. This microenvironment provides a niche for CSCs. As the content of ECM collagen I increases, CSCs undergo a metabolic shift from glucose to glutamine utilization. Notably, this transition to glutamine metabolism represents a significant collagen I‐dependent feature in CSCs [58]. Furthermore, HRGcluster A exhibited more active HH signaling and p53 signaling. The p53 is a tumor suppressor, and dysregulation of the p53 signaling pathway created via p53 mutations, deletions, or abnormalities in upstream and downstream mediators is almost universally present in all types of tumors [59]. Although there is no study showing the association between p53 and the HH pathway in PC, an experiment in gastric cancer provides us with a hint: the *Δ*Np63/SHH pathway can inhibit the stemness of gastric cancer cells [60].

Advanced PC is characterized by a fibrotic and immune‐excluded TME, which hinders the success of immune therapy [61, 62]. The key to unlocking immune therapy for PC may lie in treating early‐stage PC, with a clear focus on target identification and precise drug selection. The HH pathway also plays an important role in the evolution of the TME. A study in the field of breast cancer shows that the HH signal can coordinate macrophage metabolic changes, leading to immune‐suppressive M2 polarization [63]. A study from de la Roche et al. [64] found that HH signaling was involved in mediating the activation of cytotoxic T cells. Therefore, understanding the function of the HH pathway in the immunological characteristics of PC is crucial. Our results manifested that CD8+ T cells, naïve B cells, and monocytes had a higher abundance in HRGcluster B. Additionally, a higher abundance of activated dendritic cells, neutrophils, Tregs, and macrophages M0 was observed in HRGcluster A, indicating an interesting and complex result. Studies have shown that immune cells with antitumor effects, such as CD4+, CD8+ effector T lymphocytes, and NK cells, are reduced or functionally inactive in PC, while immune cells with immunosuppressive effects, like tumor‐associated macrophages, Tregs, and myeloid‐derived suppressor cells, are functionally active and proliferating, thus creating a microenvironment favorable for PC immune escape [65]. Consistent with our findings, previous studies have shown that M0 macrophages and neutrophils accumulate in PC tissue, indicating a bad outcome [66]. Existing research has shown that B cells have dual functions in the TME. Under specific conditions, B cells exhibit immunosuppressive activity, inhibiting the effector function of T cells and/or NK cells, and exerting negative regulatory effects on these immune cells, thereby assisting tumor cells in evading the immune response [67]. However, the presence of CD20+ B cells has also been found to be associated with improved prognosis in different tumors, such as melanoma, sarcoma, colorectal cancer, and biliary tract cancer [68]. Monocytes, as immune cells, can influence the TME via various mechanisms, including inducing immune tolerance, promoting angiogenesis, and enhancing tumor cell proliferation. However, they could also produce antitumor effector molecules by activating antigen‐presenting cells [69]. This study shows that monocytes and B cells are more abundant in the PC tissues of the HRGcluster B subgroup, suggesting an association with a better prognosis. The complexity of the tumor immune microenvironment makes immunotherapy one of the most popular tumor treatment methods [70, 71]. As a well‐known immune checkpoint, although the clinical efficacy of PD‐L1 blocking in PC has been observed, PD‐1/PD‐L1 inhibitors enable T lymphocytes to restore the killing function to tumor cells by removing the immunosuppressive regulation of T lymphocytes [72]. Notably, this study shows that HRGcluster B with a low HH signal score was more likely to benefit from immunotherapy. The higher level of T cell infiltration in HRGcluster B also confirms this hypothesis.

To improve the response rates of PC for adjuvant therapy, we identified the sensitivities of different drugs in two molecular subtypes. This study manifested that PC patients with HRGcluster B were more sensitive to most drugs, while PC patients with HRGcluster A were only more sensitive to selumetinib and trametinib. This may explain why HRGcluster B has a better outcome than HRGcluster A. As a broad‐spectrum anticancer agent, cisplatin in combination with gemcitabine inhibits cancer cell multiplication by blocking the replication and transcription of tumor cell DNA [73]. The anticancer effect of paclitaxel is achieved by causing multipolar spindle chromosomal disintegration in cancer cells and increasing chromosomal instability [74]. Currently, the recommended initial chemotherapy protocol for PC consists of either the FOLFIRINOX regimen, which comprises irinotecan, oxaliplatin, 5‐fluorouracil, and calcium folinate, or gemcitabine with nab‐paclitaxel. These drugs have shown effectiveness in improving patient outcomes and are commonly employed as the frontline treatment options for PC. Some studies have shown that FOLFIRINOX is more effective than single‐agent gemcitabine, but it also has more toxic side effects [75]. However, more targeted drugs are still needed to overcome drug resistance in PC. Sorafenib and erlotinib are two orally available multitarget tyrosine kinase inhibitors that selectively block the ATP‐binding site of BCR/ABL kinase, inhibit phosphorylation of tyrosine residues in BCR/ABL kinase substrates, inactivate the enzyme, and subsequently block a series of signaling pathways, leading to apoptosis in BCR/ABL‐positive cells [76]. Cyclophosphamide primarily exerts its effects on tumor immunity. Following administration, it undergoes hepatic metabolism by microsomal functional oxidase, converting it into aldophosphamide. Inside tumor cells, aldophosphamide further breaks down into acrolein and nitrogen mustard, effectively inducing the apoptosis of tumor cells. This mechanism contributes to the anticancer properties of cyclophosphamide [77]. It is worth noting that, as MEK inhibitors, selumetinib and trametinib exhibit higher sensitivity in HRGclusterA than in HRGclusterB. Consistent with current clinical studies, MEK inhibitors, as downstream drugs of KRAS, are mainly used in advanced PC patients [78].

A HH‐related prognostic predictive model that encompasses SERPINB3, LY6D, DCBLD2, and ANLN was developed in this study. SERPINB3 belongs to the serine protease inhibitor family and is located on chromosome 18q21.3. It primarily inhibits cysteine proteases such as tissue proteases K, L, S, and V. SERPINB3 has been found to have antiapoptotic, proproliferative, and promigratory effects and is frequently overexpressed in squamous cell carcinomas such as cervical cancer, lung cancer, esophageal cancer, and head and neck tumors [79]. LY6D is a gene that codes for proteins predominantly located on the cell membrane and serves as a specific marker for early lymphocyte development in B cells and T cells [80]. DCBLD2 is a type I transmembrane protein, first cloned from human coronary artery endothelial cells [81]. Consistent with our results, existing research has shown that both LY6D and DCBLD2 exhibit high expression in PC and are associated with poor prognosis [82]. Feng et al. [83] indicated that DCBLD2 was upregulated in PC tissues and was a risk factor for poor prognosis. DCBLD2 is primarily involved in inhibiting cell immunity and activating carcinogenic signaling pathways, acting as an oncogene [84]. This study revealed, through single‐cell transcriptomics and in vitro experiments, that DCBLD2 may affect the proliferation and metastasis of PC cells by regulating the HH signaling pathway. ANLN is an actin‐binding protein that exhibits widespread expression and exerts a critical protumorigenic function in cellular processes such as growth, migration, and cytokinesis. Extensive research has consistently indicated the upregulation of ANLN in diverse cancer types, along with its substantial associations with patient prognosis and cancer characteristics [85]. Idichi et al. [86] suggested that microRNA‐217 could inhibit the aggressiveness of PC cells by downregulating ANLN. This study indicated that ANLN was upregulated in PC and was linked to a poor outcome.

This study is the first to use bioinformatics and experimental validation to identify the molecular characteristics of HH signaling in PC and reveal that DCBLD2 promotes PC progression by regulating HH signaling. Nevertheless, this study has certain limitations. First, the dataset used in our study was from a publicly available database and was retrospective. Further validation of our results in future multicenter prospective studies is necessary. Second, although we detected that DCBLD2 knockdown could downregulate HH signaling and inhibit the proliferation, migration, and invasion of PC cells. Further, rescue experiments are also necessary in future in‐depth studies.

## 5. Conclusion

This study reveals the molecular characteristics of HH signaling in PC, which is closely associated with PC development, progression, prognosis, TME, and treatment response. SERPINB3, LY6D, DCBLD2, and ANLN are upregulated in PC and associated with poor prognosis and can serve as potential therapeutic targets for PC. In vitro experiments revealed that DCBLD2 can promote PC progression by regulating HH signaling, further highlighting its potential as a therapeutic target. In summary, the comprehensive characterization of HH signaling in PC provides new insights and theoretical foundations for the diagnosis and treatment of PC.

## Ethics Statement

The authors have nothing to report.

## Disclosure

All authors approved the submitted version

## Conflicts of Interest

The authors declare no conflicts of interest.

## Author Contributions

Biao Zhang, Bingqian Huang, Chongchan Bao, Jinming Liu, and Zhizhou Wang conceptualized and designed the study. Biao Zhang and Bingqian Huang collected and analyzed the data. Xinya Zhao, Biao Zhang, Bingqian Huang, and Bolin Zhang wrote the article and conducted experiments. Chongchan Bao, Jinming Liu, and Zhizhou Wang revised the manuscript. All authors contributed to the article. Biao Zhang, Bingqian Huang, and Xinya Zhao contributed equally to this study.

## Funding

This study was supported by the Medical Science Research Project of Dalian City, 2212006.

## Supporting Information

Additional supporting information can be found online in the Supporting Information section.

## Supporting information


**Supporting Information 1** Figure S1: The quality control of scRNA‐seq data. Figure S2: UMAP plot of cell subset annotations based on Human Primary Cell Atlas Data. Figure S3: The expression of the typical marker gene in each cell cluster. Figure S4: The percentage of each cell subset in the total number of cells.

**Supporting Information 2** Table S1: Hedgehog signaling–related genes. Table S2: The primer sequences of SERPINB3, LY6D, DCBLD2, and ANLN. Table S3: The prognosis‐linked HRDEGs were screened using univariable Cox regression.

**Supporting Information 3** Supplementary raw data and images. The raw data and images generated in this study are uploaded to a zip file named “Raw data and images.” The raw data include data from PCR, CCK‐8, scratch, and Transwell invasion assays. The raw images also include images from the scratch and transwell invasion assays.

## Data Availability

The data that support the findings of this study are available in the supporting information of this article.
