# Supplementary figures and images for: Integrated Multiomics Unravels Hedgehog (HH) Signaling Characteristics in Pancreatic Cancer (PC) and DCBLD2 Regulates HH Signaling to Drive PC Progression
Source: Hum Mutat. 2025 Dec 11;2025:4806397. doi: 10.1155/humu/4806397 (PMC12714175; doi:10.1155/humu/4806397)

**nFeature\_RNA**

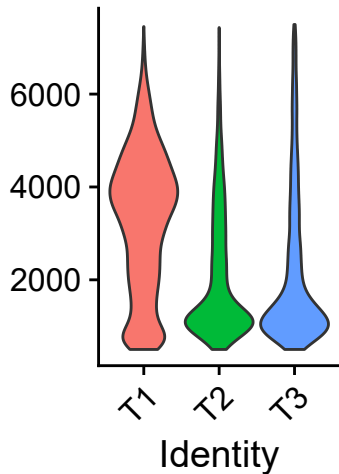

**nCount\_RNA**

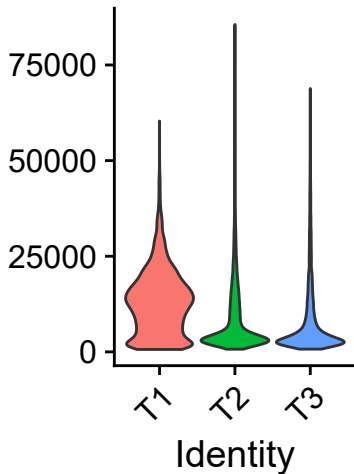

**percent.mt**

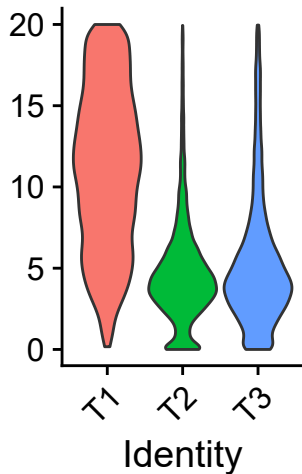

Supplement: Supplementary file 1 — Supporting Information 1 Figure S1: The quality control of scRNA‐seq data. Figure S2: UMAP plot of cell subset annotations based on Human Primary Cell Atlas Data. Figure S3: The expression of the typical marker gene in each cell cluster. Figure S4: The percentage of each cell subset in the total number of cells. [file HUMU-2025-4806397-s003.zip › Supporting Information 1/Figure S1.pdf]

# celltype

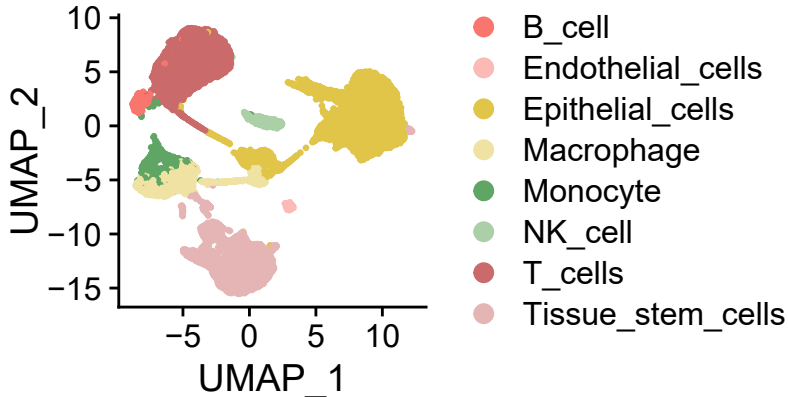

Supplement: Supplementary file 1 — Supporting Information 1 Figure S1: The quality control of scRNA‐seq data. Figure S2: UMAP plot of cell subset annotations based on Human Primary Cell Atlas Data. Figure S3: The expression of the typical marker gene in each cell cluster. Figure S4: The percentage of each cell subset in the total number of cells. [file HUMU-2025-4806397-s003.zip › Supporting Information 1/Figure S2.pdf]

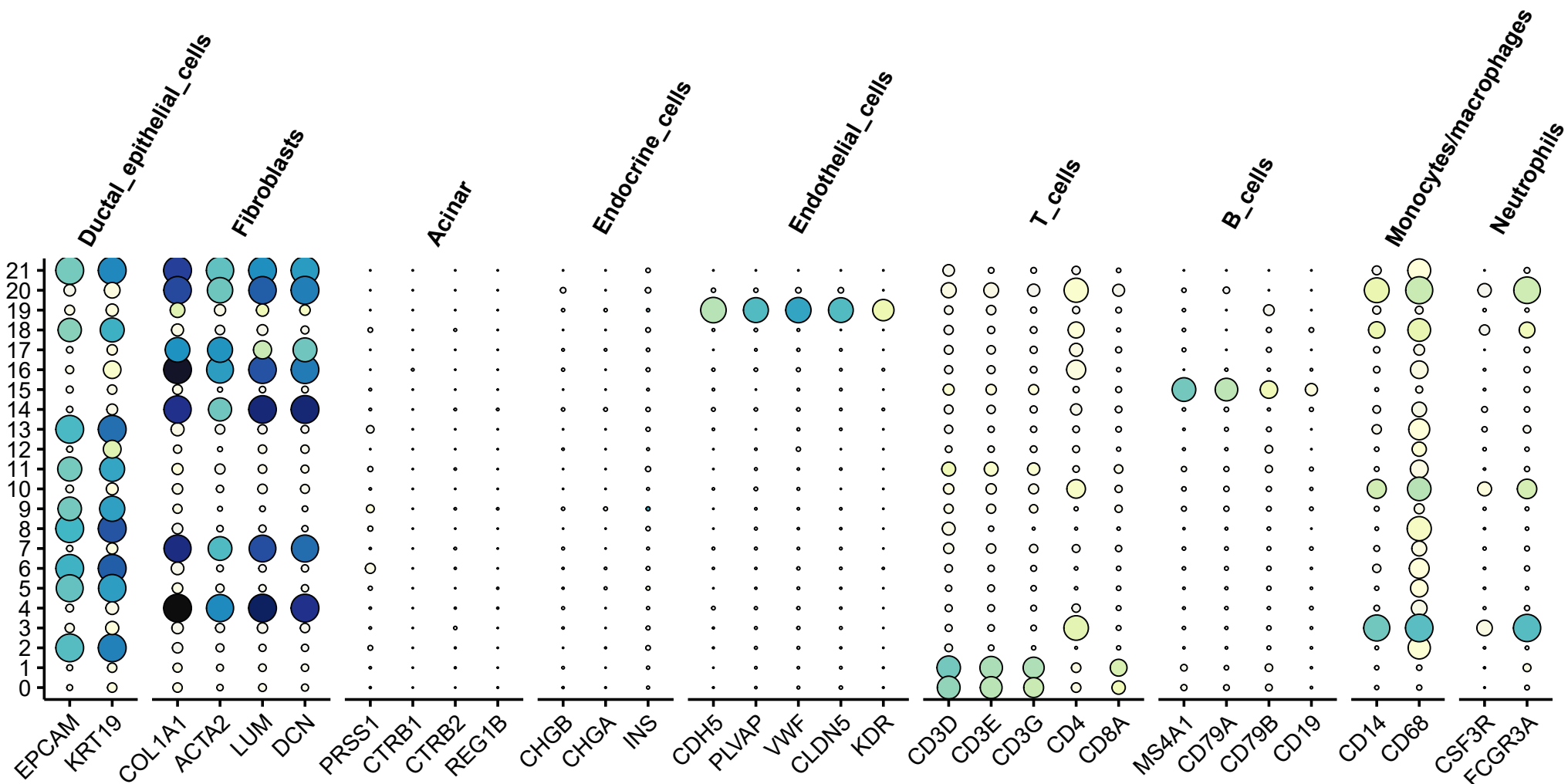

Percent Expressed

0 25 50 75 100

Avg. Expression

0

1

2

3

4

Supplement: Supplementary file 1 — Supporting Information 1 Figure S1: The quality control of scRNA‐seq data. Figure S2: UMAP plot of cell subset annotations based on Human Primary Cell Atlas Data. Figure S3: The expression of the typical marker gene in each cell cluster. Figure S4: The percentage of each cell subset in the total number of cells. [file HUMU-2025-4806397-s003.zip › Supporting Information 1/Figure S3.pdf]

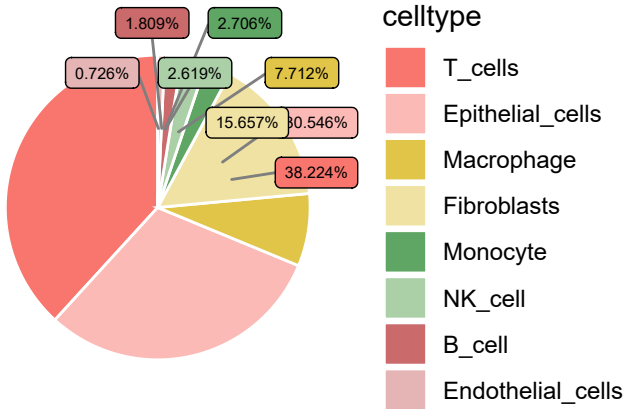

Supplement: Supplementary file 1 — Supporting Information 1 Figure S1: The quality control of scRNA‐seq data. Figure S2: UMAP plot of cell subset annotations based on Human Primary Cell Atlas Data. Figure S3: The expression of the typical marker gene in each cell cluster. Figure S4: The percentage of each cell subset in the total number of cells. [file HUMU-2025-4806397-s003.zip › Supporting Information 1/Figure S4.pdf]

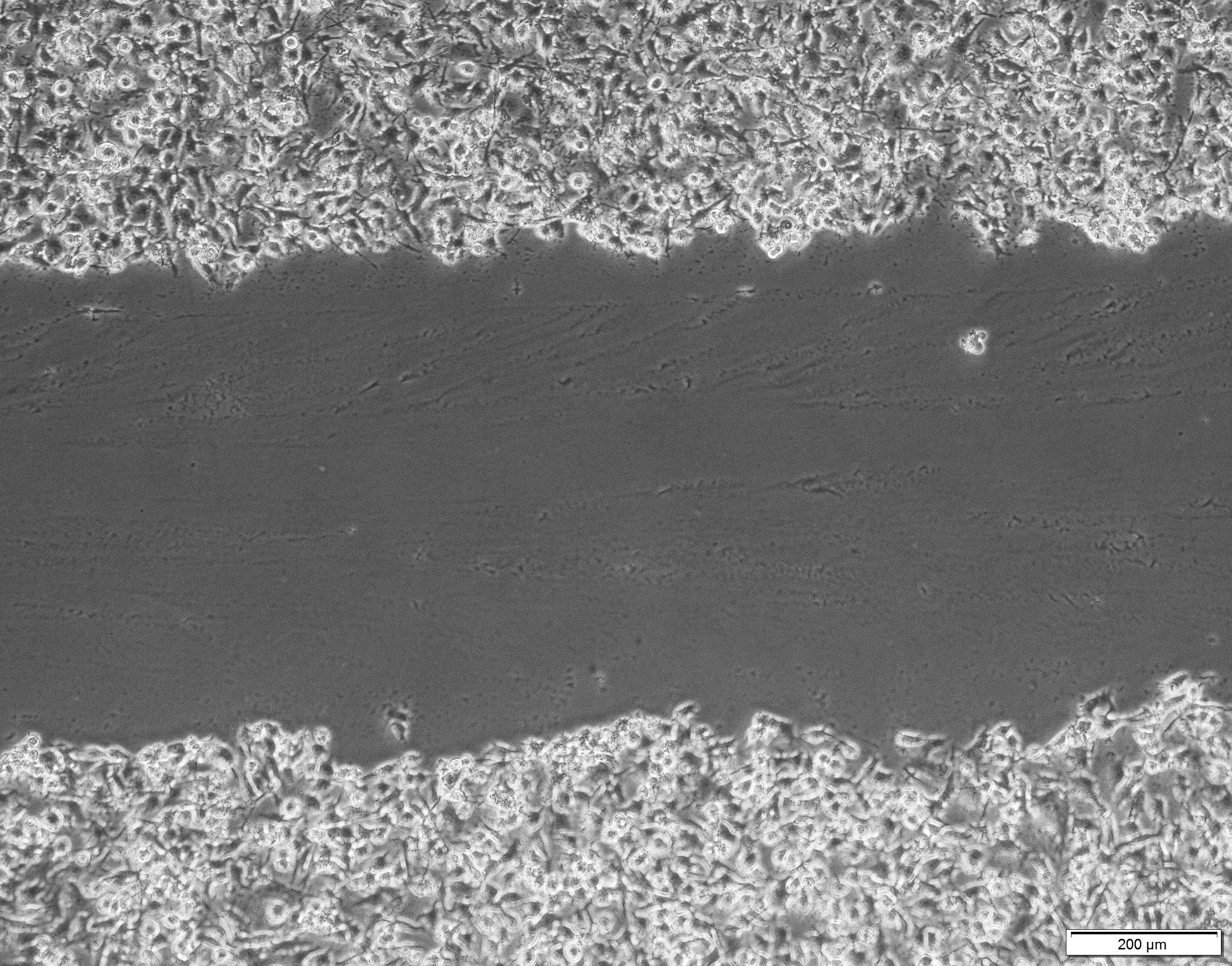

Supplement: Supplementary file 3 — Supporting Information 3 Supplementary raw data and images. The raw data and images generated in this study are uploaded to a zip file named “Raw data and images.” The raw data include data from PCR, CCK‐8, scratch, and Transwell invasion assays. The raw images also include images from the scratch and transwell invasion assays. [file HUMU-2025-4806397-s002.zip › Raw data and images/Raw images/Figure 14I/siNC Day-0 (PANC1).tif]

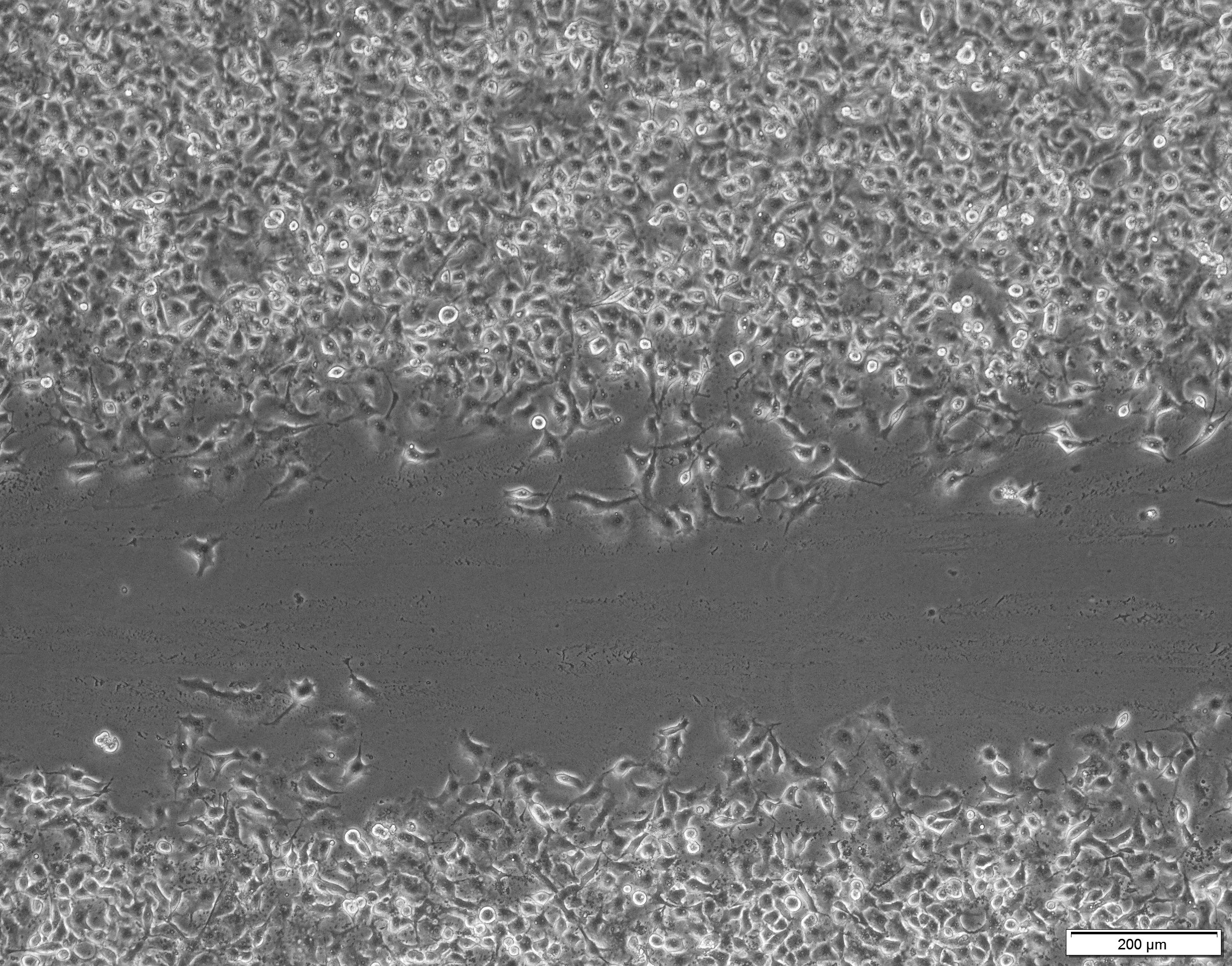

Supplement: Supplementary file 3 — Supporting Information 3 Supplementary raw data and images. The raw data and images generated in this study are uploaded to a zip file named “Raw data and images.” The raw data include data from PCR, CCK‐8, scratch, and Transwell invasion assays. The raw images also include images from the scratch and transwell invasion assays. [file HUMU-2025-4806397-s002.zip › Raw data and images/Raw images/Figure 14I/siNC Day-1 (PANC1).tif]

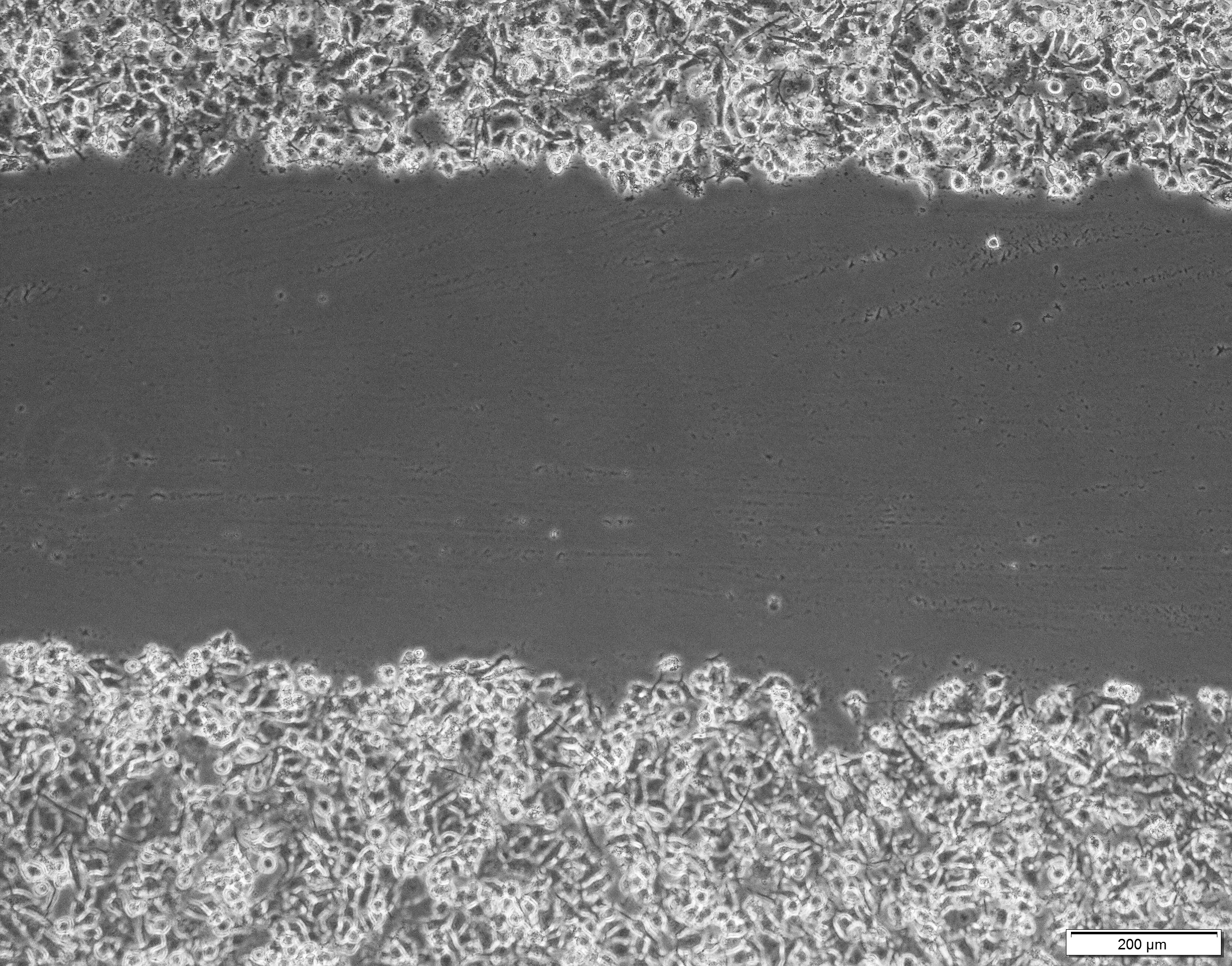

Supplement: Supplementary file 3 — Supporting Information 3 Supplementary raw data and images. The raw data and images generated in this study are uploaded to a zip file named “Raw data and images.” The raw data include data from PCR, CCK‐8, scratch, and Transwell invasion assays. The raw images also include images from the scratch and transwell invasion assays. [file HUMU-2025-4806397-s002.zip › Raw data and images/Raw images/Figure 14I/siRNA#1 Day-0 (PANC1).tif]

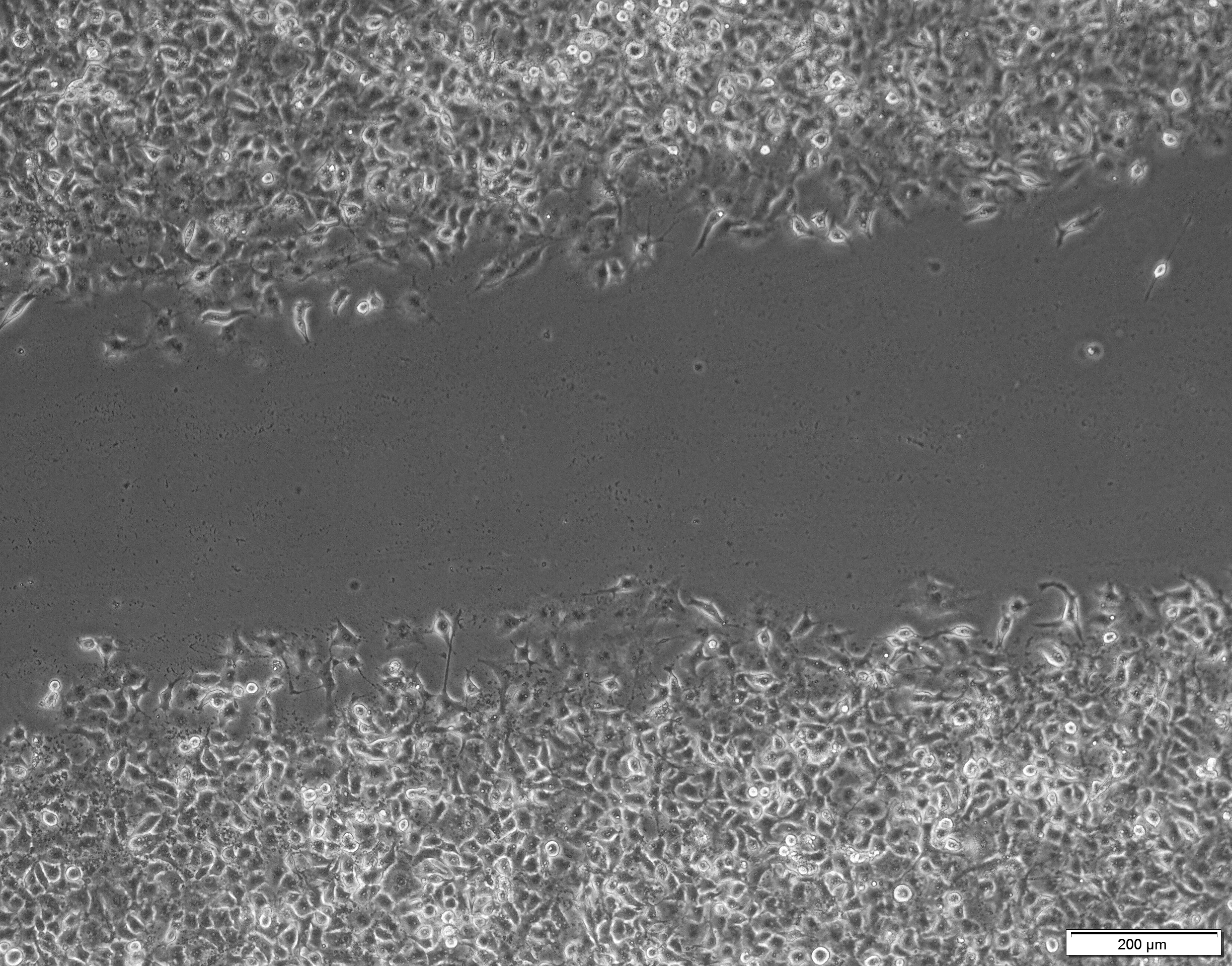

Supplement: Supplementary file 3 — Supporting Information 3 Supplementary raw data and images. The raw data and images generated in this study are uploaded to a zip file named “Raw data and images.” The raw data include data from PCR, CCK‐8, scratch, and Transwell invasion assays. The raw images also include images from the scratch and transwell invasion assays. [file HUMU-2025-4806397-s002.zip › Raw data and images/Raw images/Figure 14I/siRNA#1 Day-1 (PANC1).tif]

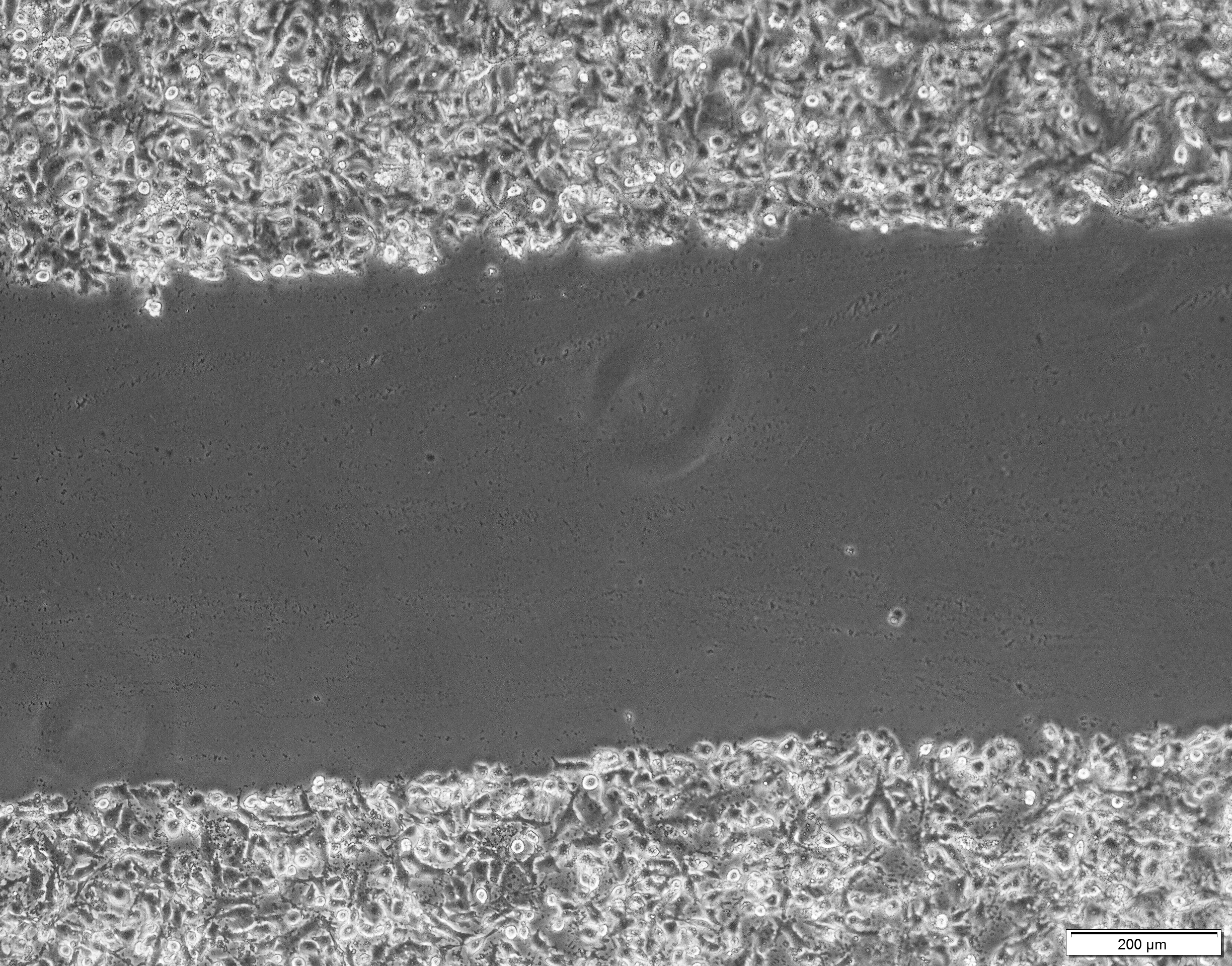

Supplement: Supplementary file 3 — Supporting Information 3 Supplementary raw data and images. The raw data and images generated in this study are uploaded to a zip file named “Raw data and images.” The raw data include data from PCR, CCK‐8, scratch, and Transwell invasion assays. The raw images also include images from the scratch and transwell invasion assays. [file HUMU-2025-4806397-s002.zip › Raw data and images/Raw images/Figure 14I/siRNA#2 Day-0 (PANC1).tif]

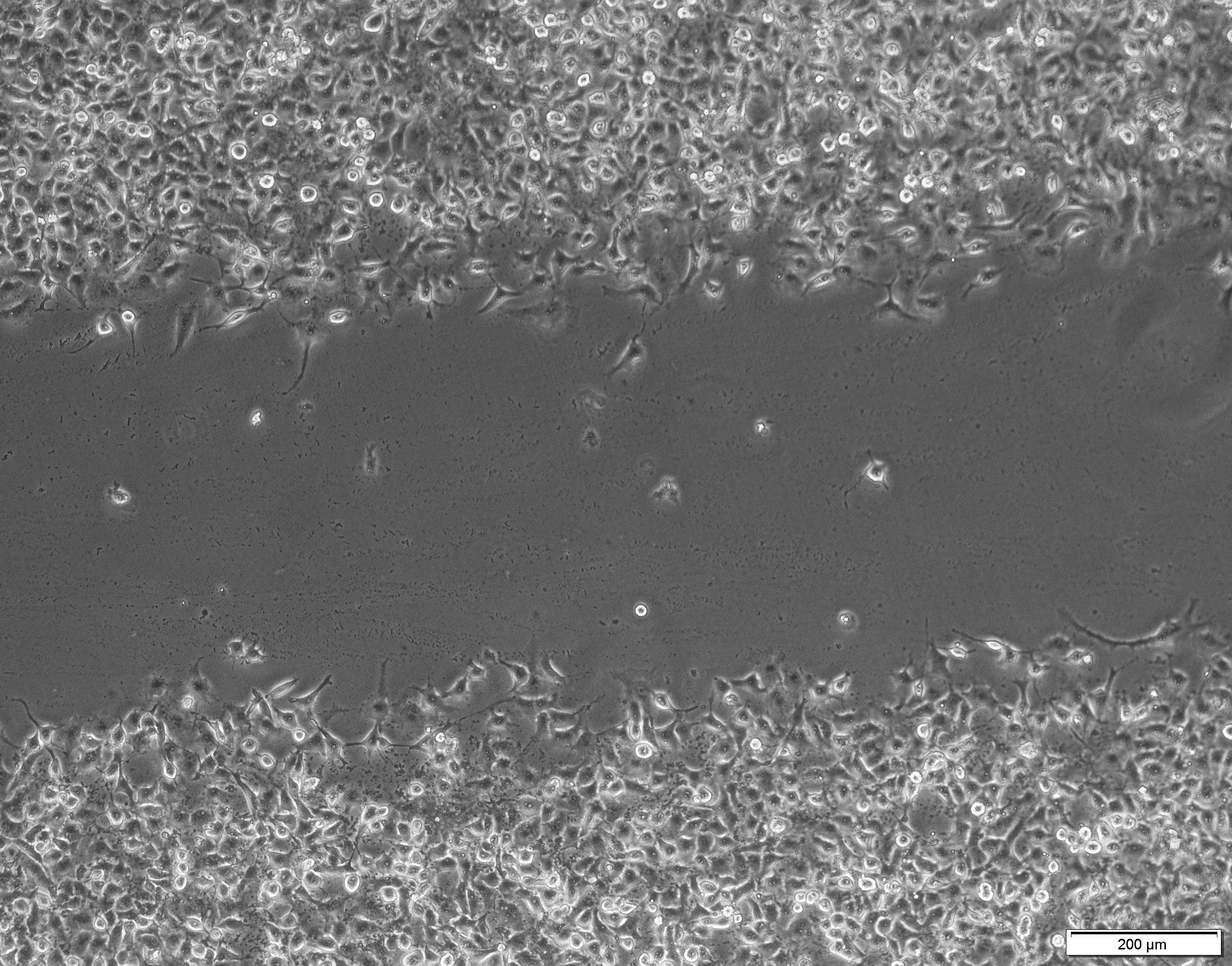

Supplement: Supplementary file 3 — Supporting Information 3 Supplementary raw data and images. The raw data and images generated in this study are uploaded to a zip file named “Raw data and images.” The raw data include data from PCR, CCK‐8, scratch, and Transwell invasion assays. The raw images also include images from the scratch and transwell invasion assays. [file HUMU-2025-4806397-s002.zip › Raw data and images/Raw images/Figure 14I/siRNA#2 Day-1 (PANC1).tif]

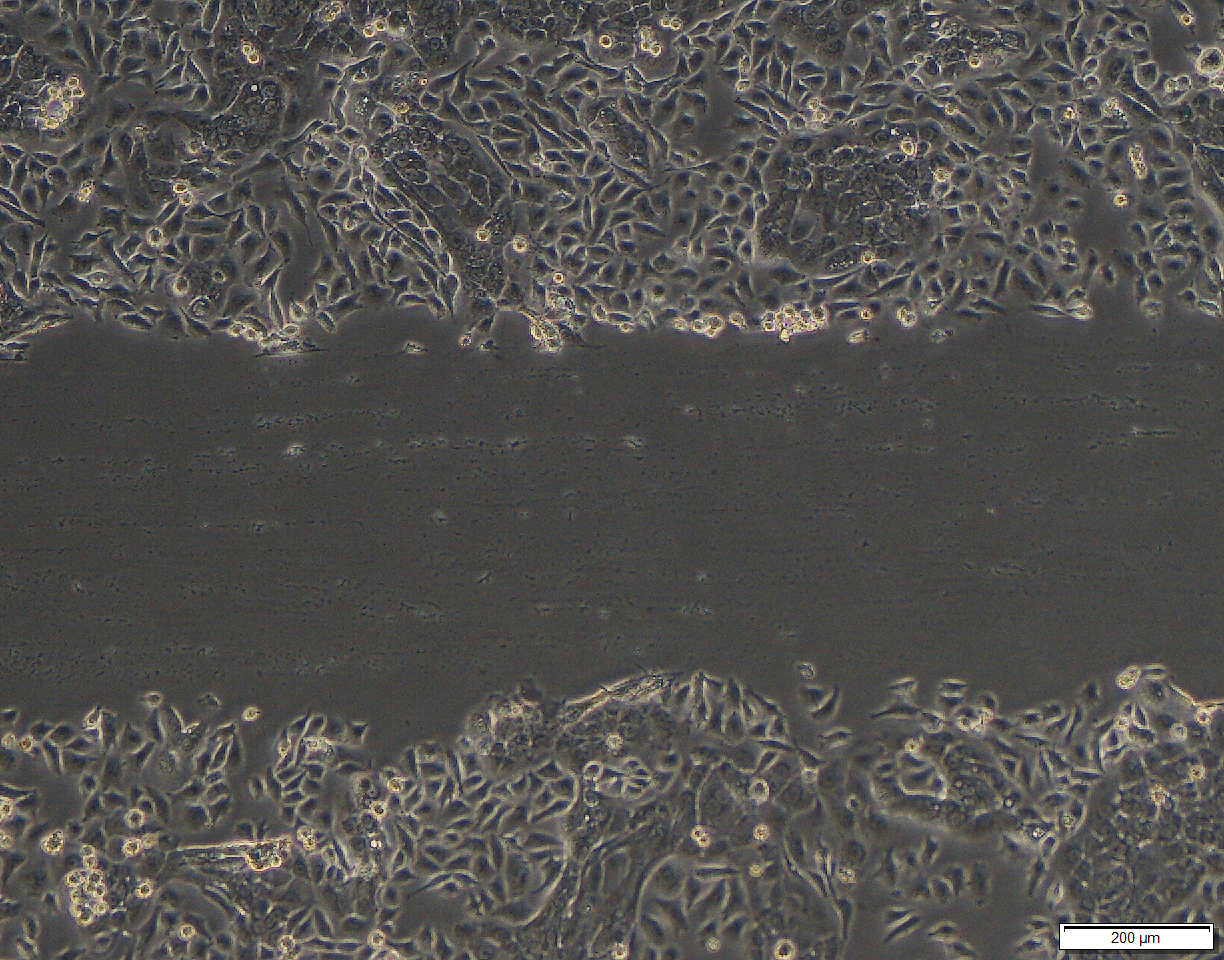

Supplement: Supplementary file 3 — Supporting Information 3 Supplementary raw data and images. The raw data and images generated in this study are uploaded to a zip file named “Raw data and images.” The raw data include data from PCR, CCK‐8, scratch, and Transwell invasion assays. The raw images also include images from the scratch and transwell invasion assays. [file HUMU-2025-4806397-s002.zip › Raw data and images/Raw images/Figure 14J/siNC Day-0 (BxPC3).tif]

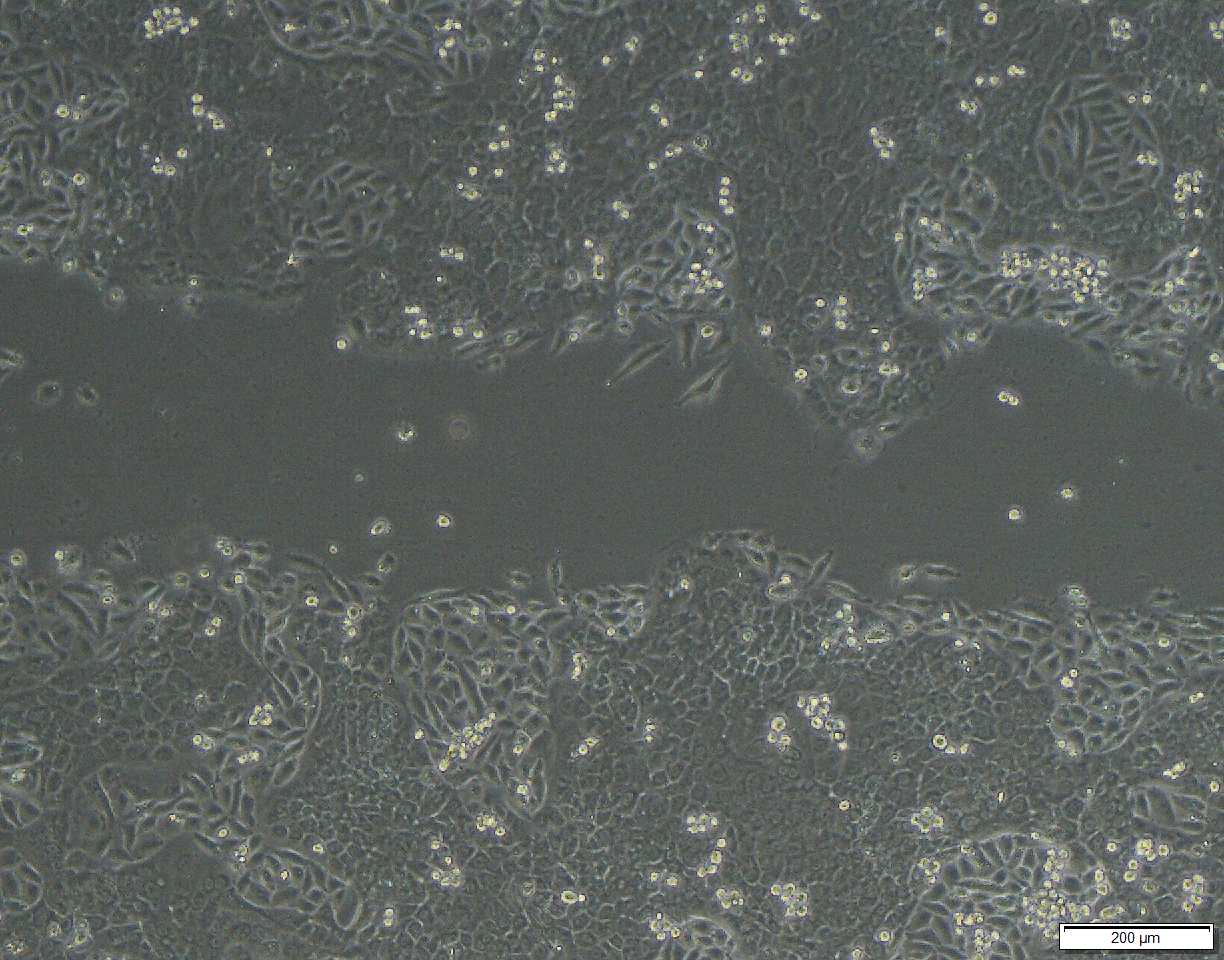

Supplement: Supplementary file 3 — Supporting Information 3 Supplementary raw data and images. The raw data and images generated in this study are uploaded to a zip file named “Raw data and images.” The raw data include data from PCR, CCK‐8, scratch, and Transwell invasion assays. The raw images also include images from the scratch and transwell invasion assays. [file HUMU-2025-4806397-s002.zip › Raw data and images/Raw images/Figure 14J/siNC Day-1 (BxPC3).tif]

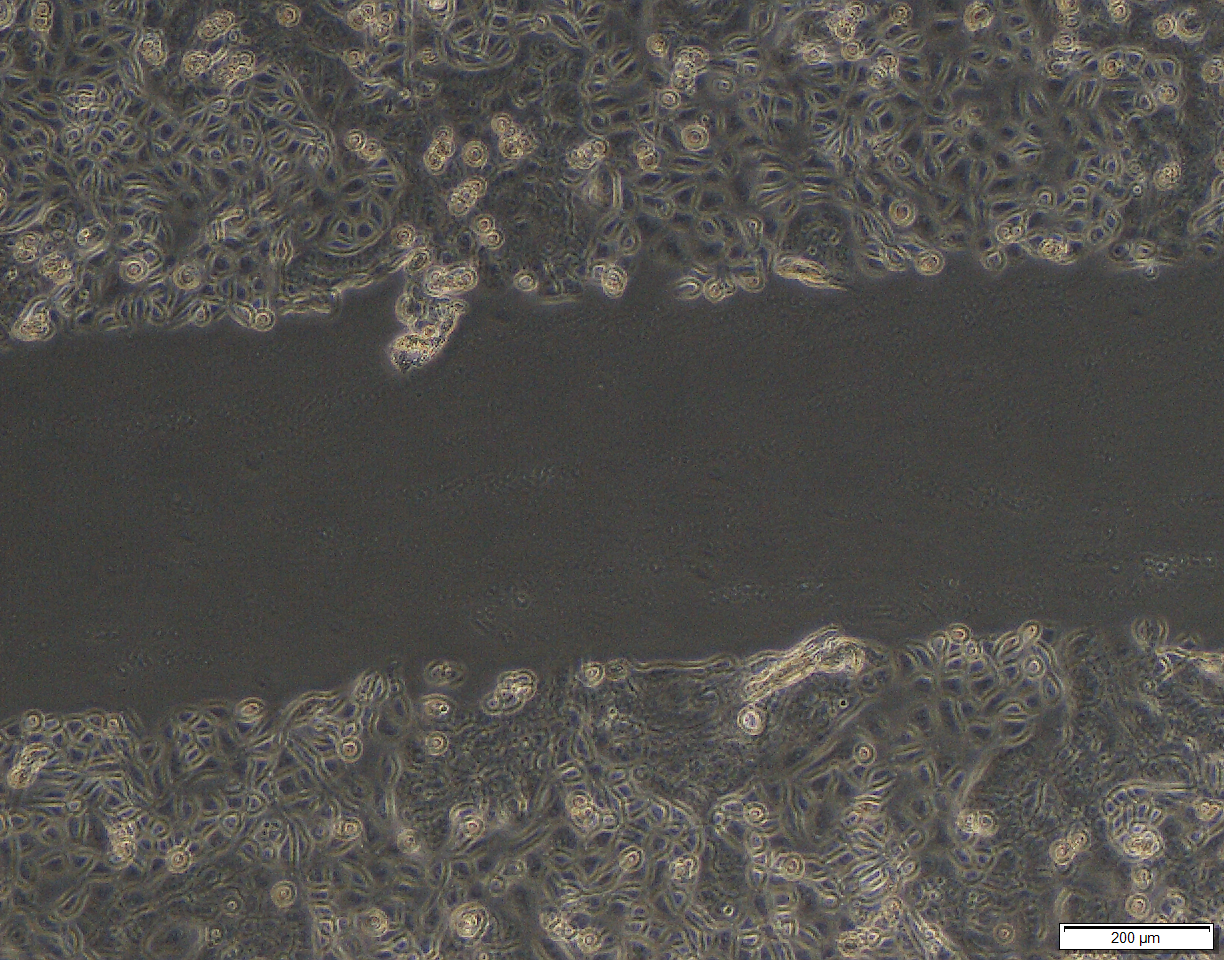

Supplement: Supplementary file 3 — Supporting Information 3 Supplementary raw data and images. The raw data and images generated in this study are uploaded to a zip file named “Raw data and images.” The raw data include data from PCR, CCK‐8, scratch, and Transwell invasion assays. The raw images also include images from the scratch and transwell invasion assays. [file HUMU-2025-4806397-s002.zip › Raw data and images/Raw images/Figure 14J/siRNA#1 Day-0 (BxPC3).tif]

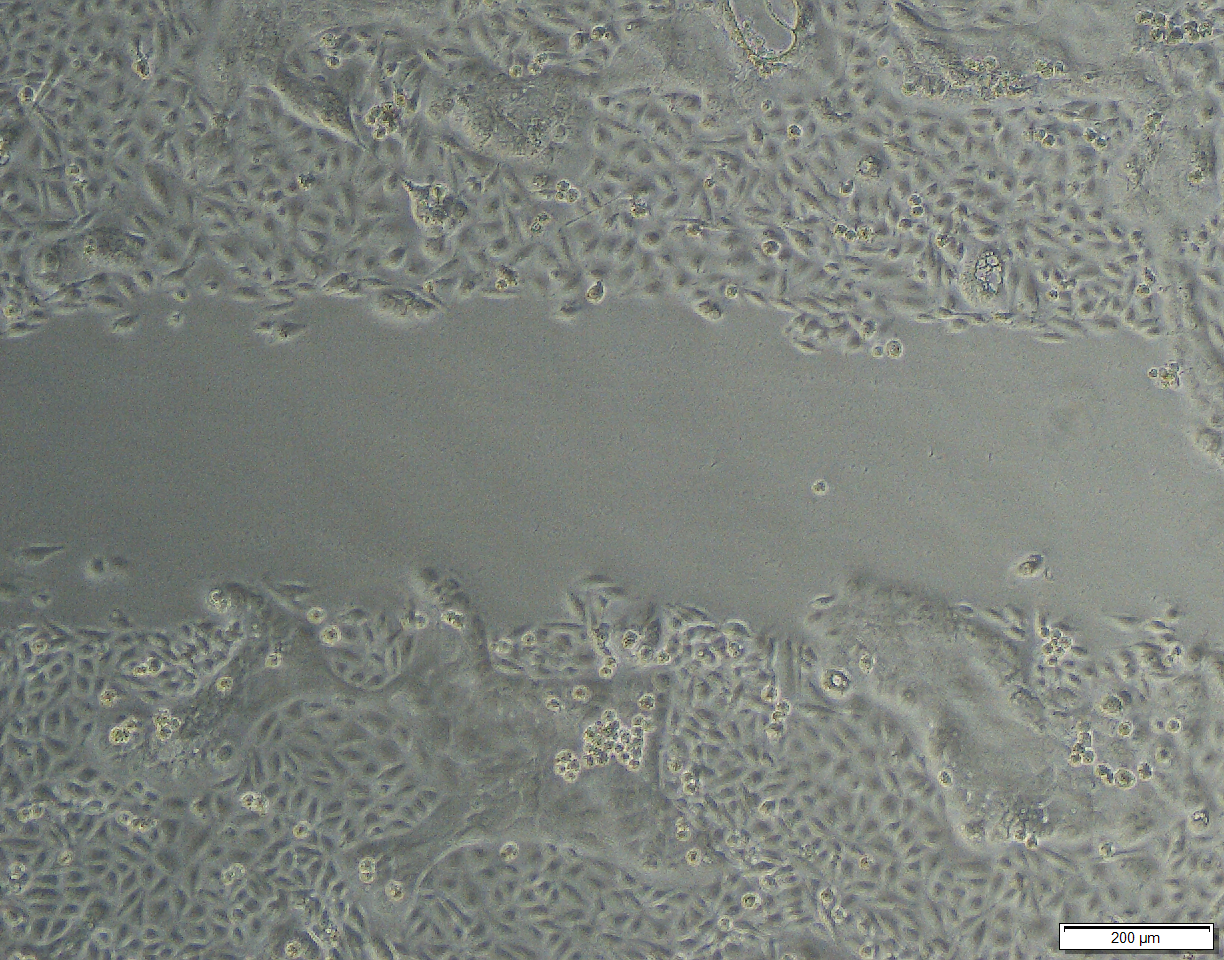

Supplement: Supplementary file 3 — Supporting Information 3 Supplementary raw data and images. The raw data and images generated in this study are uploaded to a zip file named “Raw data and images.” The raw data include data from PCR, CCK‐8, scratch, and Transwell invasion assays. The raw images also include images from the scratch and transwell invasion assays. [file HUMU-2025-4806397-s002.zip › Raw data and images/Raw images/Figure 14J/siRNA#1 Day-1 (BxPC3).tif]

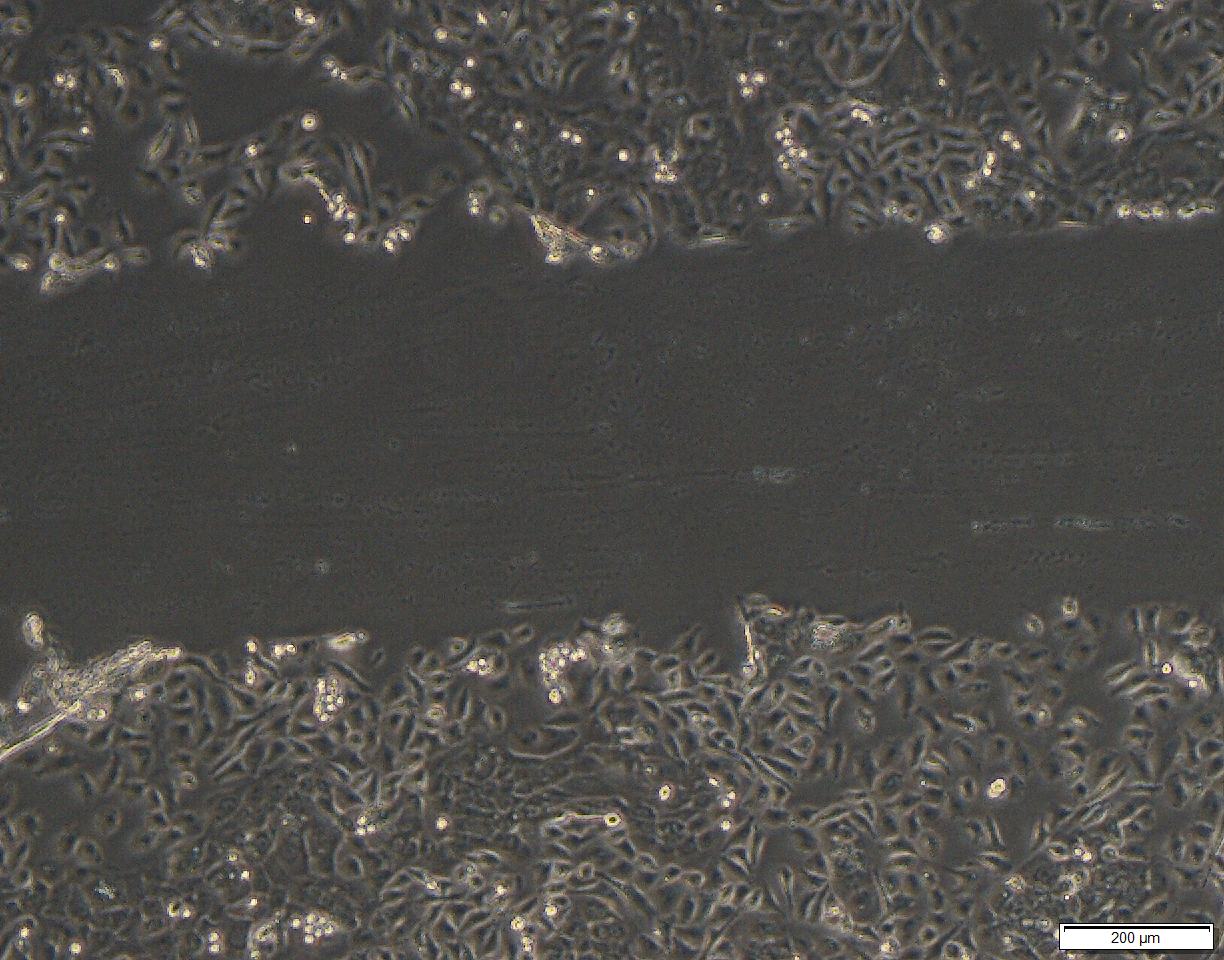

Supplement: Supplementary file 3 — Supporting Information 3 Supplementary raw data and images. The raw data and images generated in this study are uploaded to a zip file named “Raw data and images.” The raw data include data from PCR, CCK‐8, scratch, and Transwell invasion assays. The raw images also include images from the scratch and transwell invasion assays. [file HUMU-2025-4806397-s002.zip › Raw data and images/Raw images/Figure 14J/siRNA#2 Day-0 (BxPC3).tif]

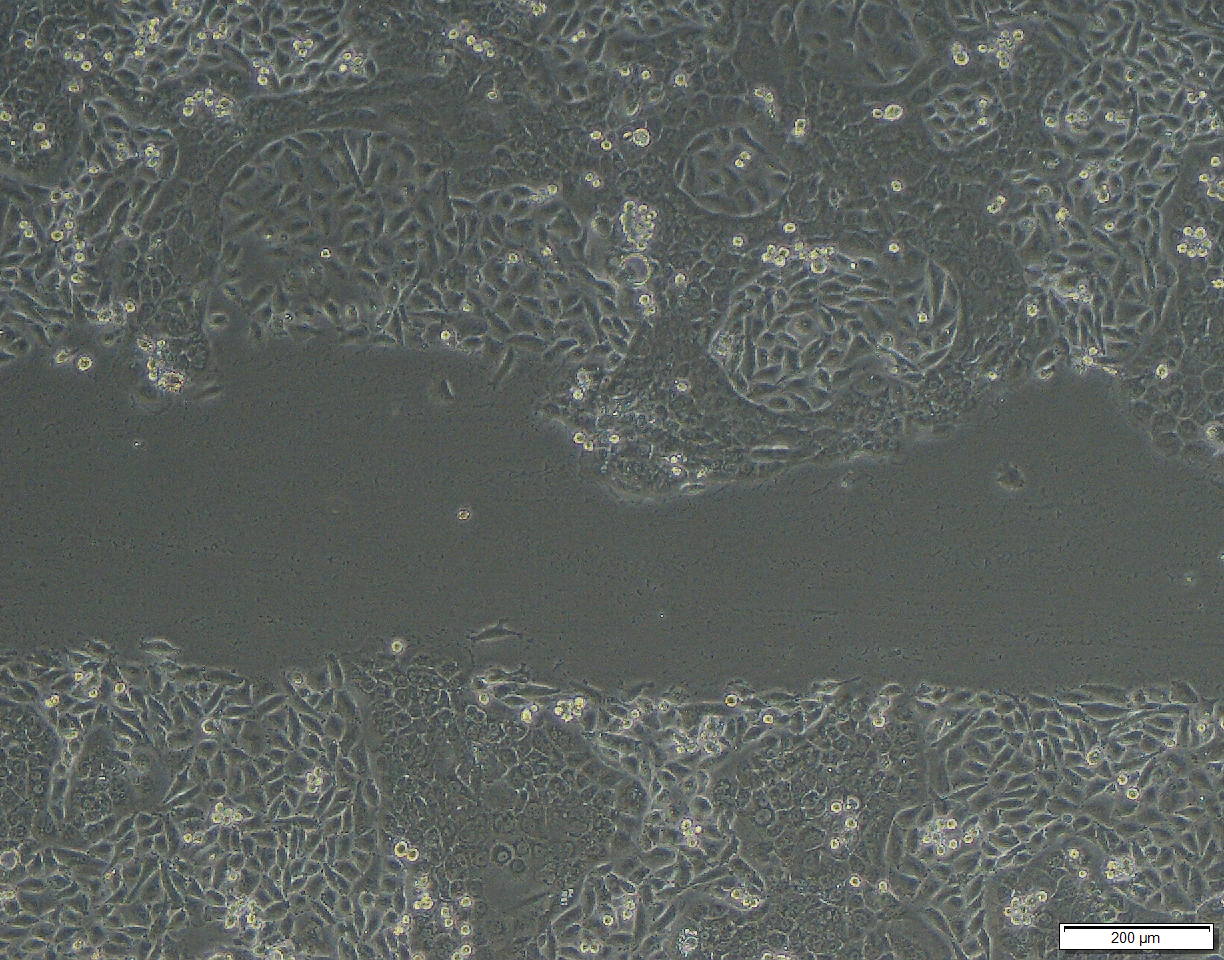

Supplement: Supplementary file 3 — Supporting Information 3 Supplementary raw data and images. The raw data and images generated in this study are uploaded to a zip file named “Raw data and images.” The raw data include data from PCR, CCK‐8, scratch, and Transwell invasion assays. The raw images also include images from the scratch and transwell invasion assays. [file HUMU-2025-4806397-s002.zip › Raw data and images/Raw images/Figure 14J/siRNA#2 Day-1 (BxPC3).tif]

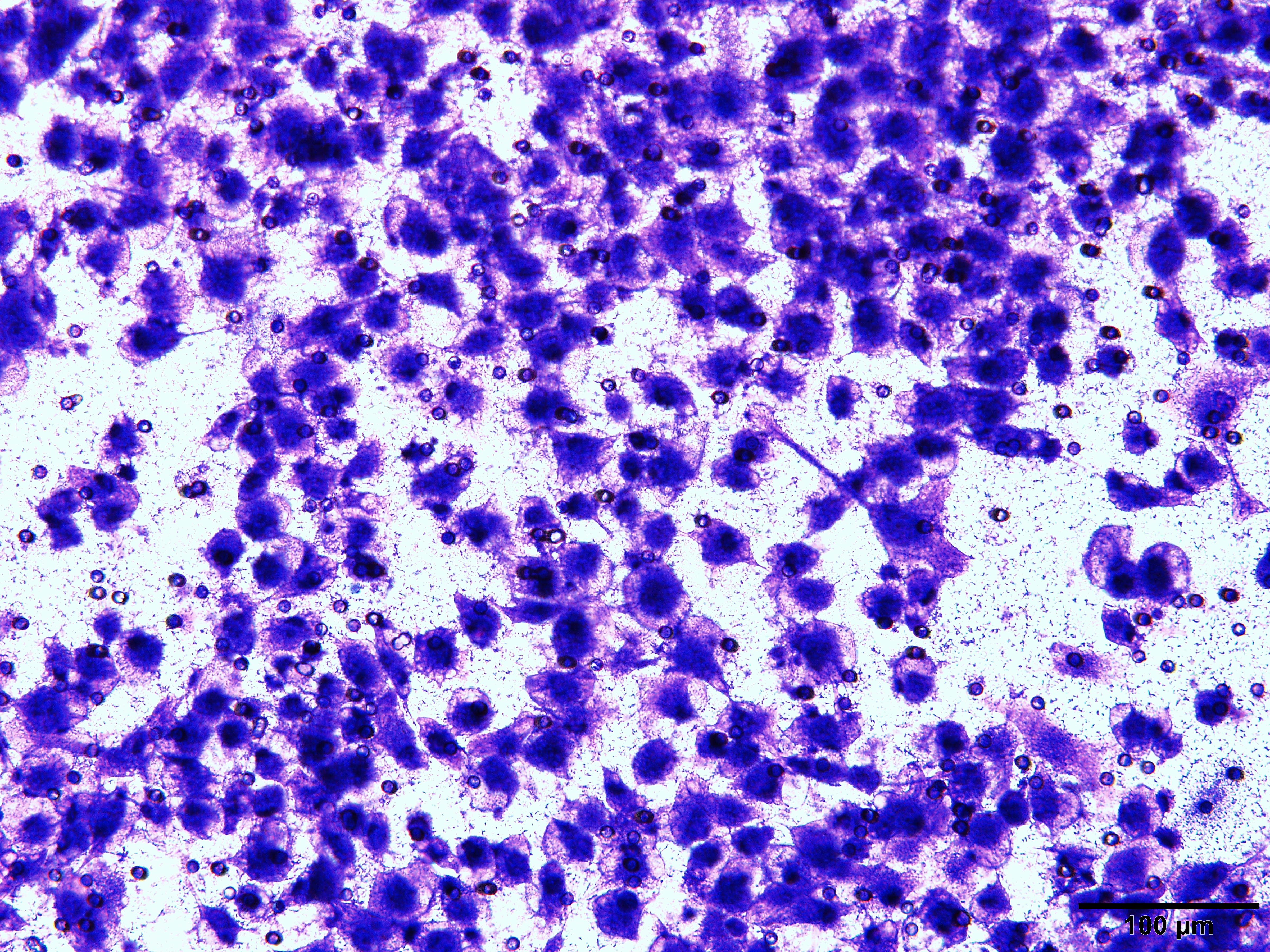

Supplement: Supplementary file 3 — Supporting Information 3 Supplementary raw data and images. The raw data and images generated in this study are uploaded to a zip file named “Raw data and images.” The raw data include data from PCR, CCK‐8, scratch, and Transwell invasion assays. The raw images also include images from the scratch and transwell invasion assays. [file HUMU-2025-4806397-s002.zip › Raw data and images/Raw images/Figure 14K/siNC (PANC1).tif]

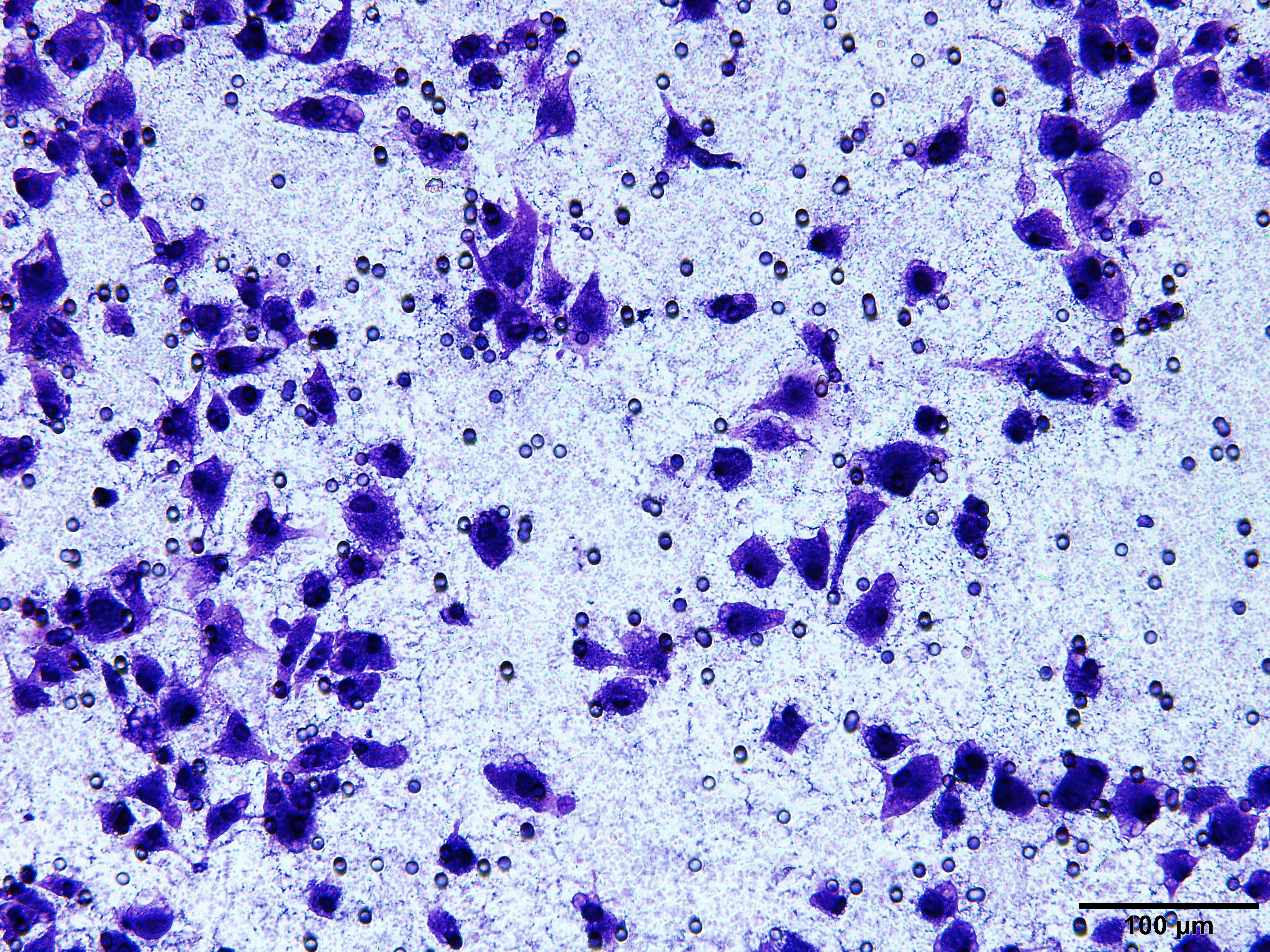

Supplement: Supplementary file 3 — Supporting Information 3 Supplementary raw data and images. The raw data and images generated in this study are uploaded to a zip file named “Raw data and images.” The raw data include data from PCR, CCK‐8, scratch, and Transwell invasion assays. The raw images also include images from the scratch and transwell invasion assays. [file HUMU-2025-4806397-s002.zip › Raw data and images/Raw images/Figure 14K/siRNA#1 (PANC1).tif]

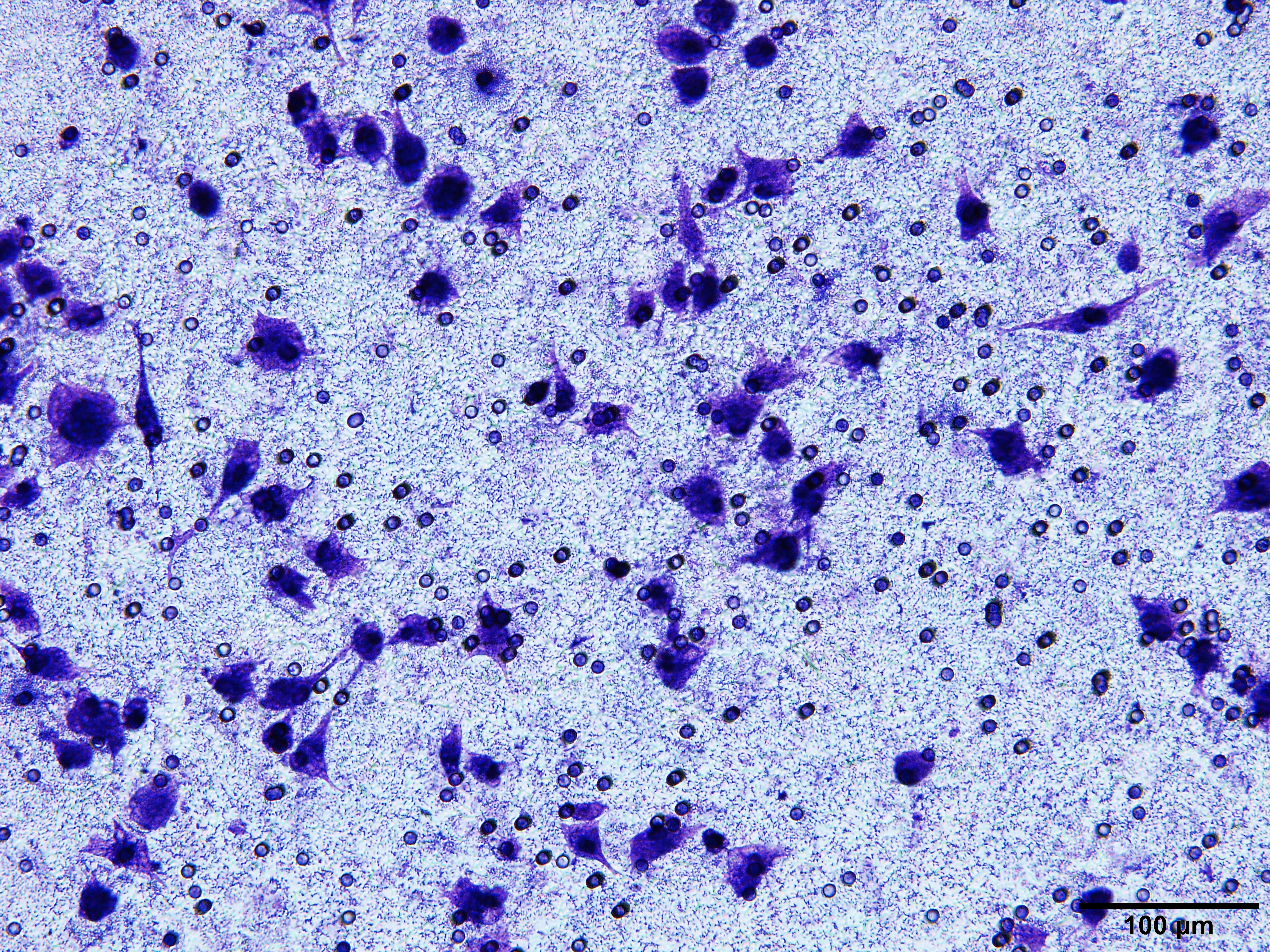

Supplement: Supplementary file 3 — Supporting Information 3 Supplementary raw data and images. The raw data and images generated in this study are uploaded to a zip file named “Raw data and images.” The raw data include data from PCR, CCK‐8, scratch, and Transwell invasion assays. The raw images also include images from the scratch and transwell invasion assays. [file HUMU-2025-4806397-s002.zip › Raw data and images/Raw images/Figure 14K/siRNA#2 (PANC1).tif]

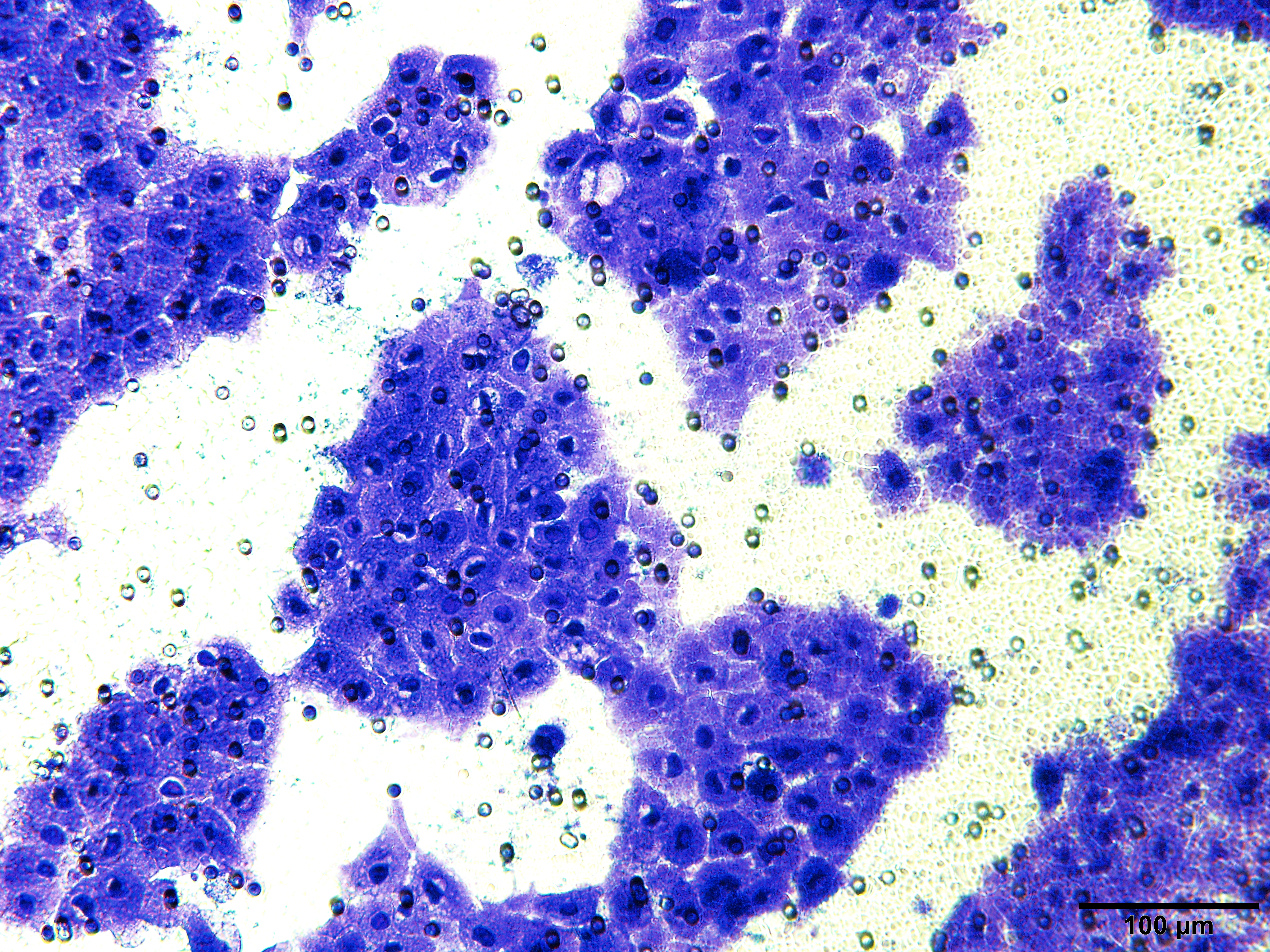

Supplement: Supplementary file 3 — Supporting Information 3 Supplementary raw data and images. The raw data and images generated in this study are uploaded to a zip file named “Raw data and images.” The raw data include data from PCR, CCK‐8, scratch, and Transwell invasion assays. The raw images also include images from the scratch and transwell invasion assays. [file HUMU-2025-4806397-s002.zip › Raw data and images/Raw images/Figure 14L/siNC (BxPC3).tif]

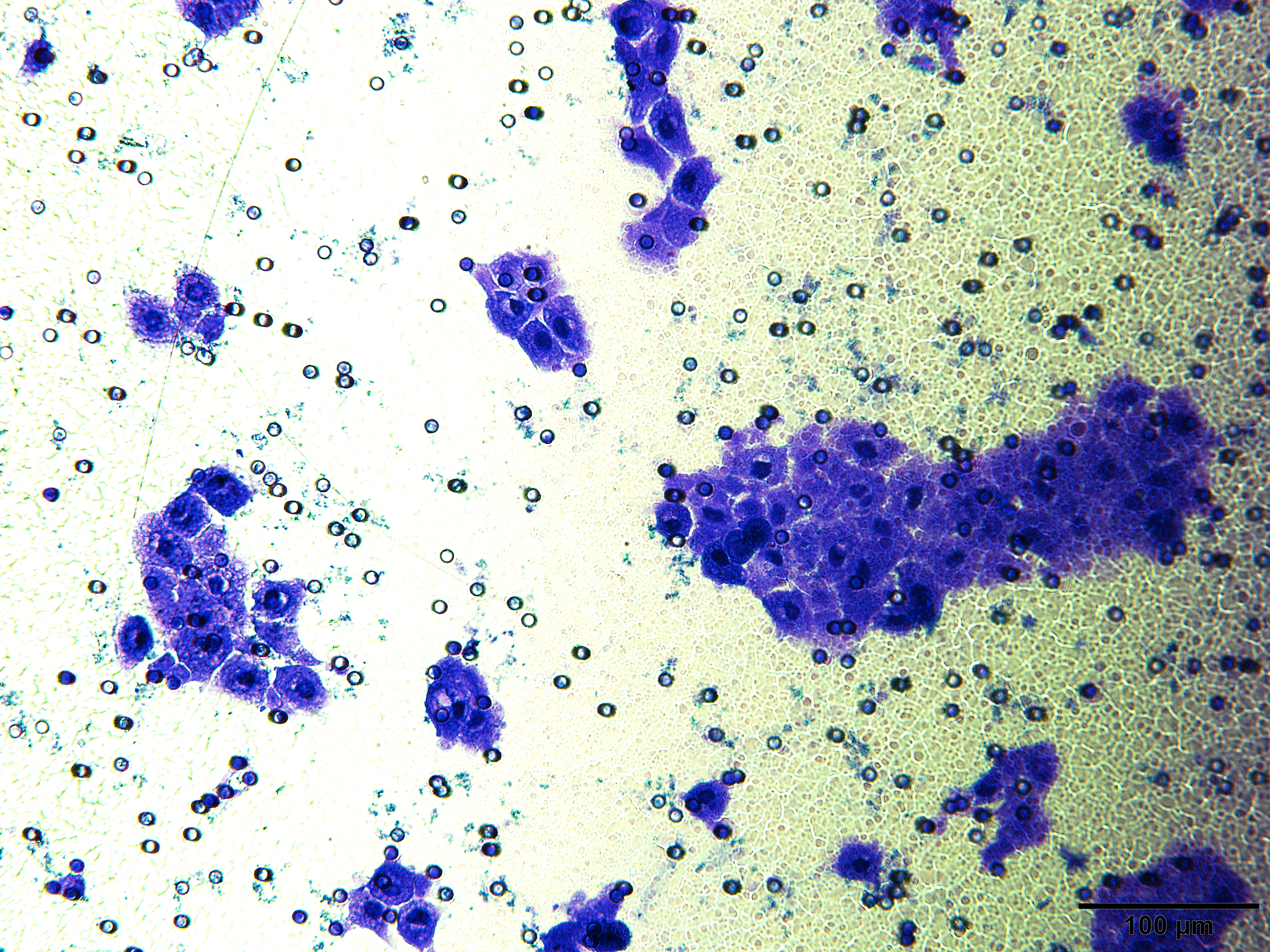

Supplement: Supplementary file 3 — Supporting Information 3 Supplementary raw data and images. The raw data and images generated in this study are uploaded to a zip file named “Raw data and images.” The raw data include data from PCR, CCK‐8, scratch, and Transwell invasion assays. The raw images also include images from the scratch and transwell invasion assays. [file HUMU-2025-4806397-s002.zip › Raw data and images/Raw images/Figure 14L/siRNA#1 (BxPC3).tif]

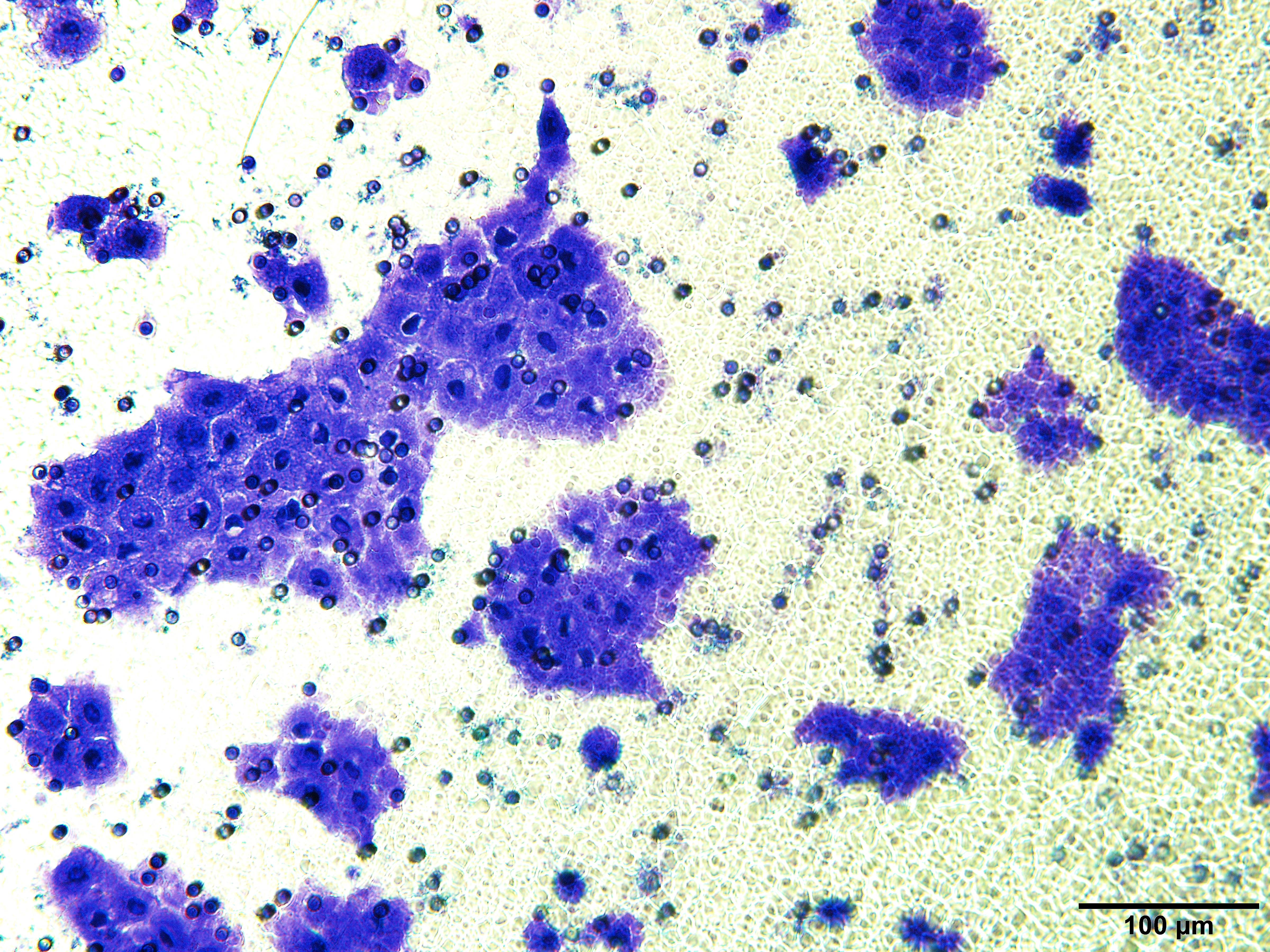

Supplement: Supplementary file 3 — Supporting Information 3 Supplementary raw data and images. The raw data and images generated in this study are uploaded to a zip file named “Raw data and images.” The raw data include data from PCR, CCK‐8, scratch, and Transwell invasion assays. The raw images also include images from the scratch and transwell invasion assays. [file HUMU-2025-4806397-s002.zip › Raw data and images/Raw images/Figure 14L/siRNA#2 (BxPC3).tif]
